# Supplementary figures and images for: Mutations that prevent phosphorylation of the BMP4 prodomain impair proteolytic maturation of homodimers leading to lethality in mice
Source: eLife. 2025 May 29;14:RP105018. doi: 10.7554/eLife.105018 (PMC12122004; doi:10.7554/eLife.105018)

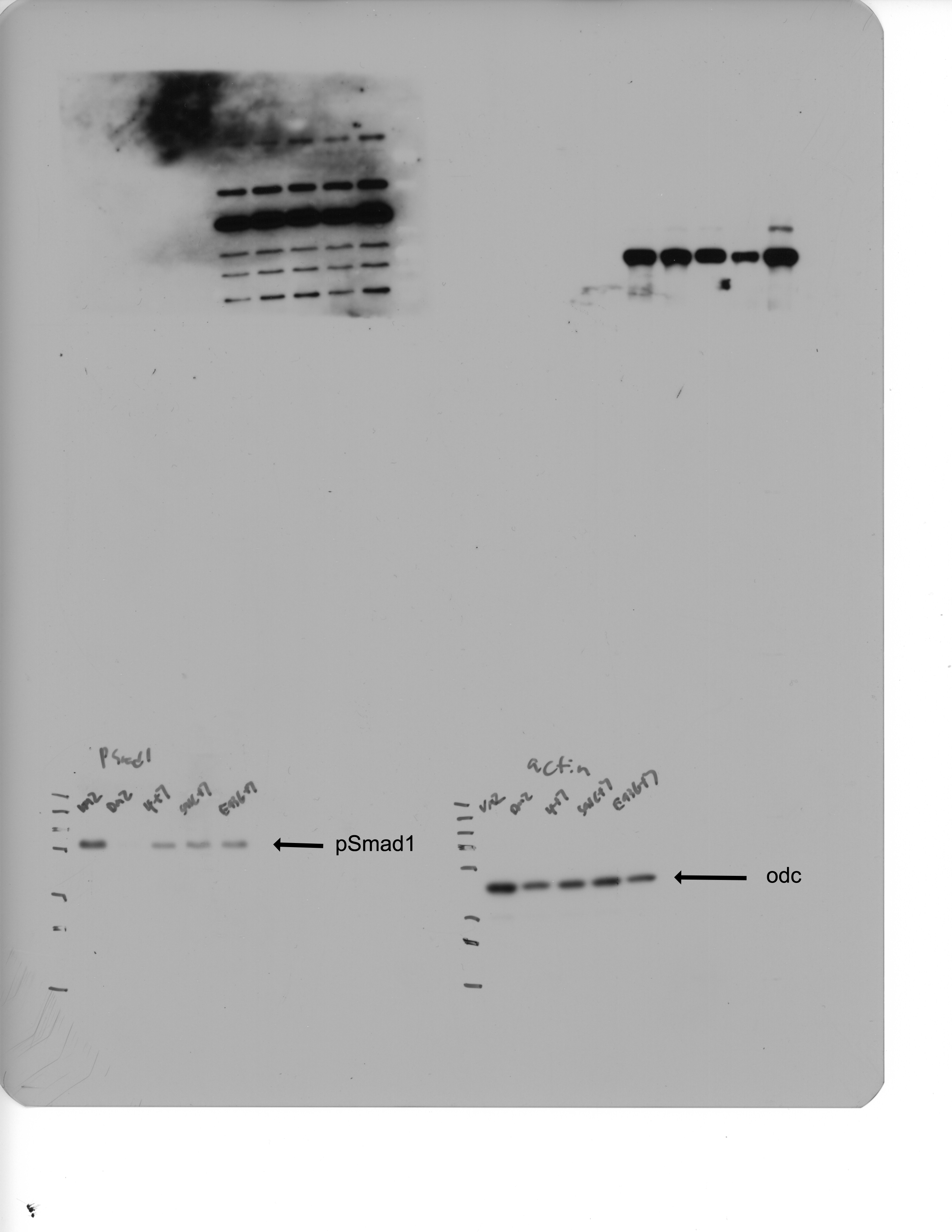

Supplement: Figure 1—source data 1. [file elife-105018-fig1-data1.zip › Figure 1-source data 1/Fig. 1D psmad actinHeterodimers -bands marked.tif]

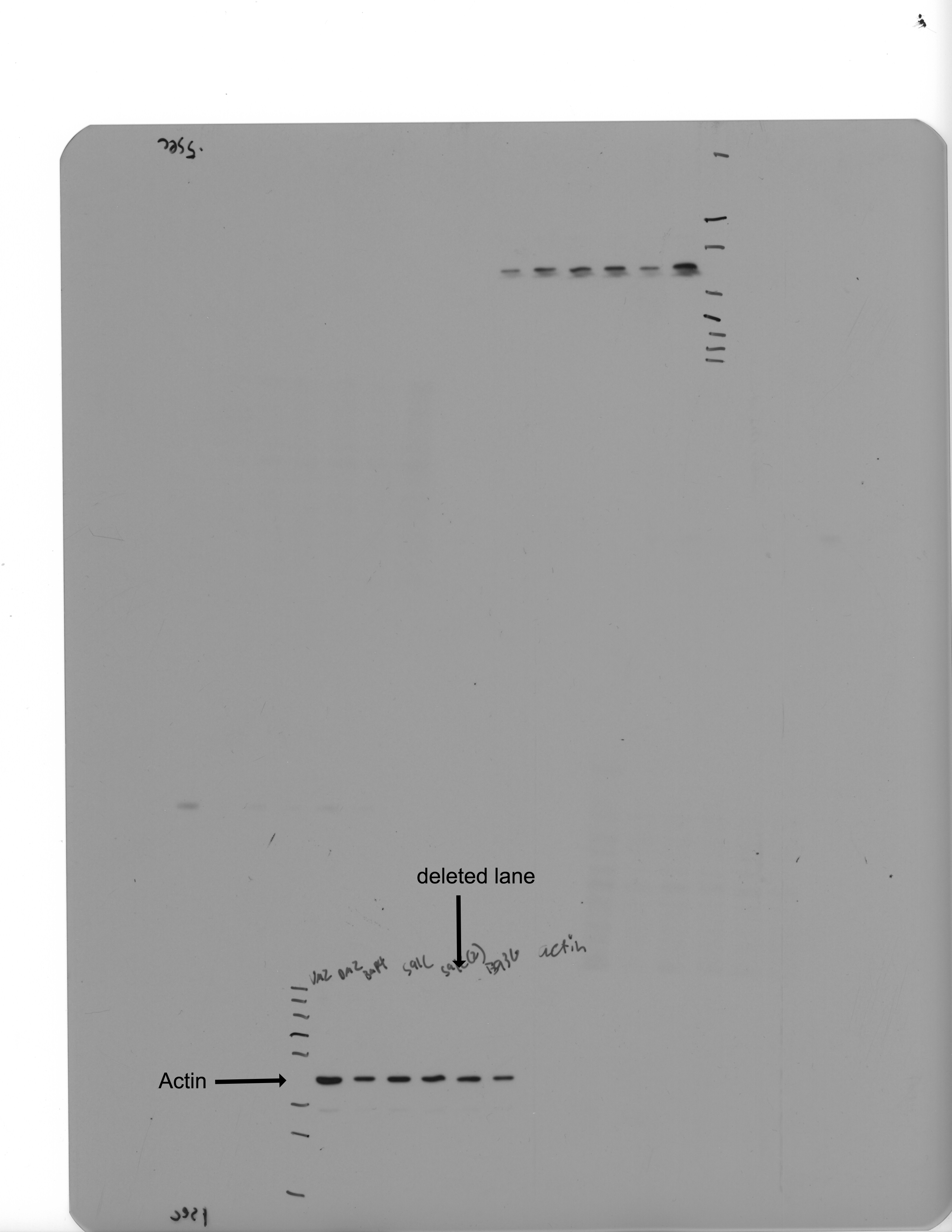

Supplement: Figure 1—source data 1. [file elife-105018-fig1-data1.zip › Figure 1-source data 1/Fig. 1D original homodimer blot actin (labeled).tif]

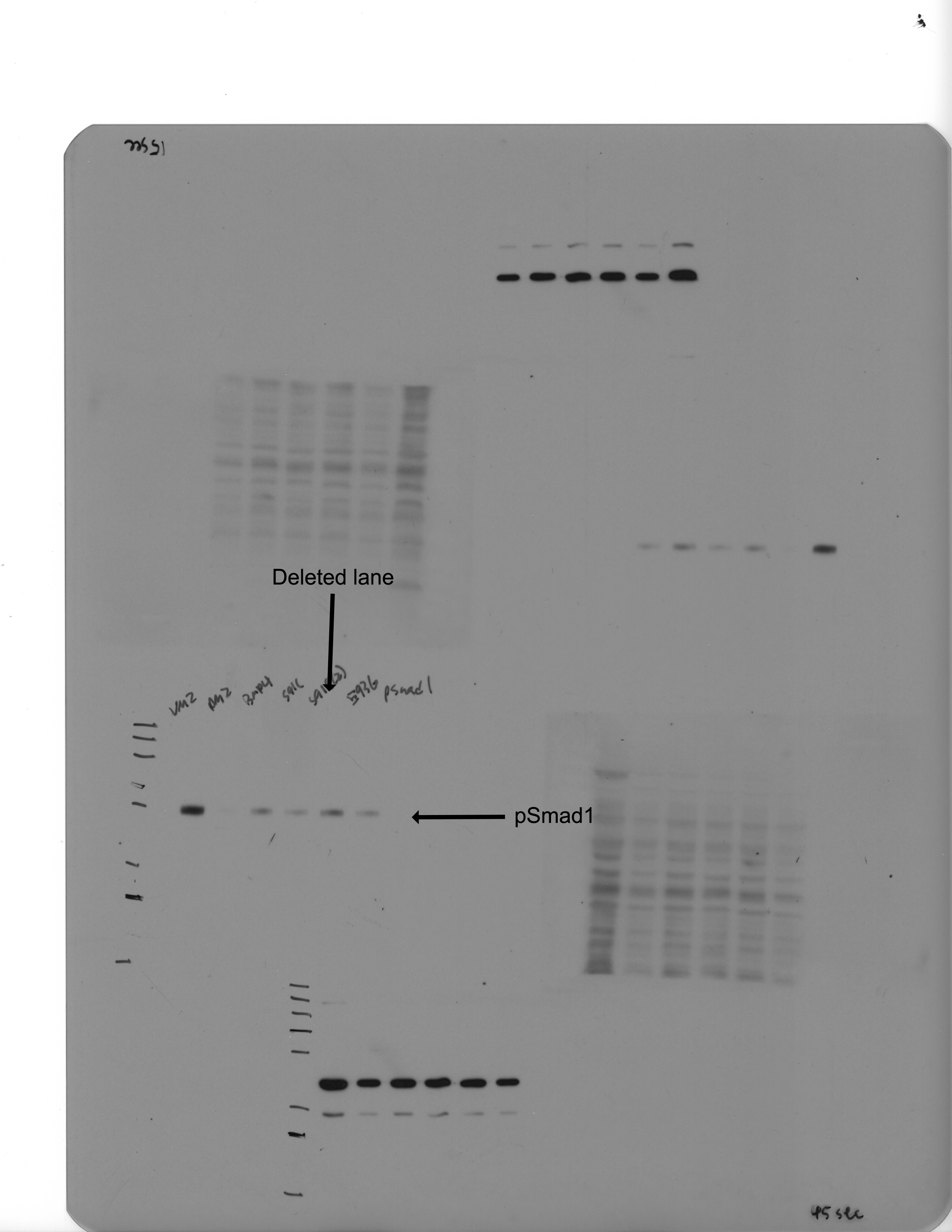

Supplement: Figure 1—source data 1. [file elife-105018-fig1-data1.zip › Figure 1-source data 1/Fig. 1D original homodimer blot pSmad-band marked.tif]

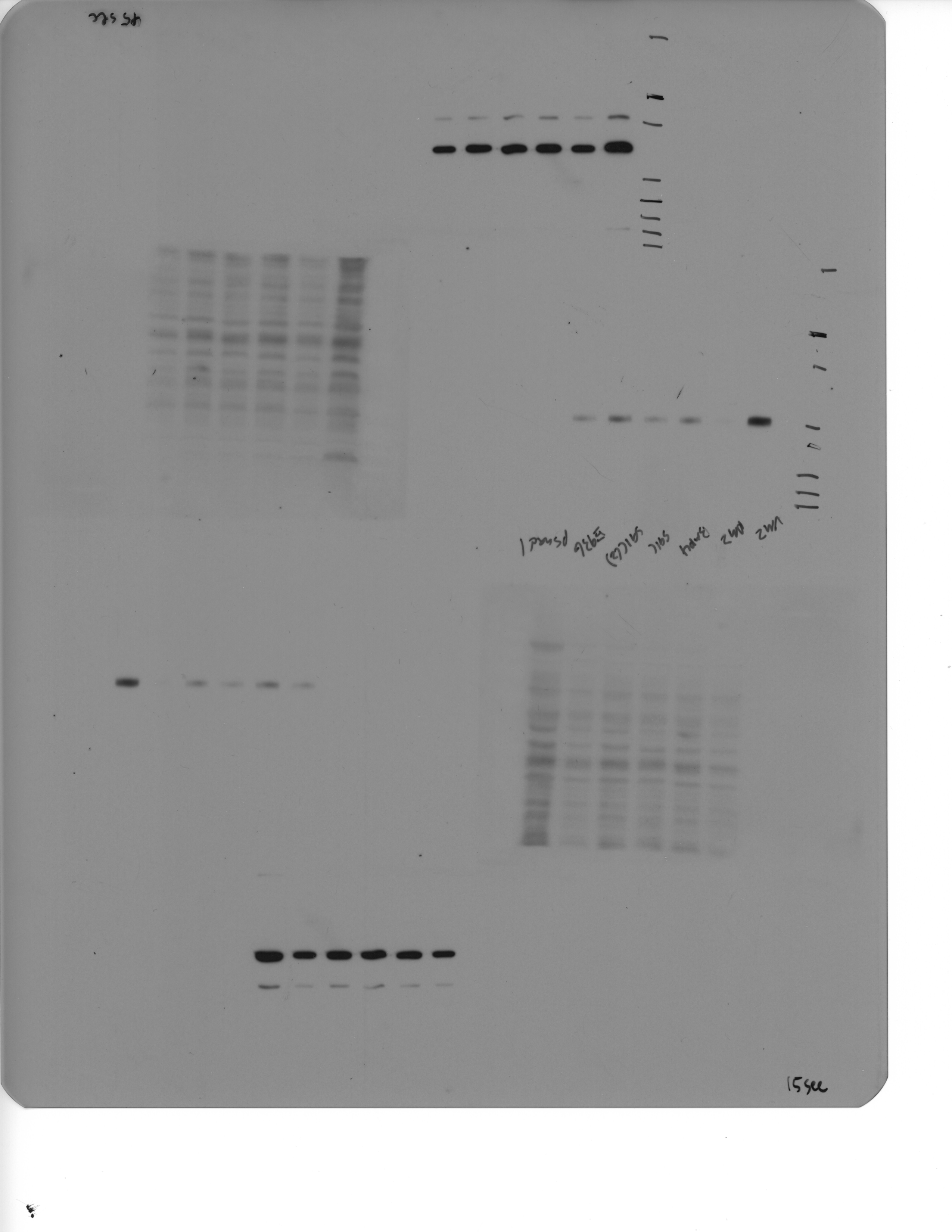

Supplement: Figure 1—source data 2. [file elife-105018-fig1-data2.zip › Figure 1-source data 2/Fig. 1D original homodimer blot pSmad.tif]

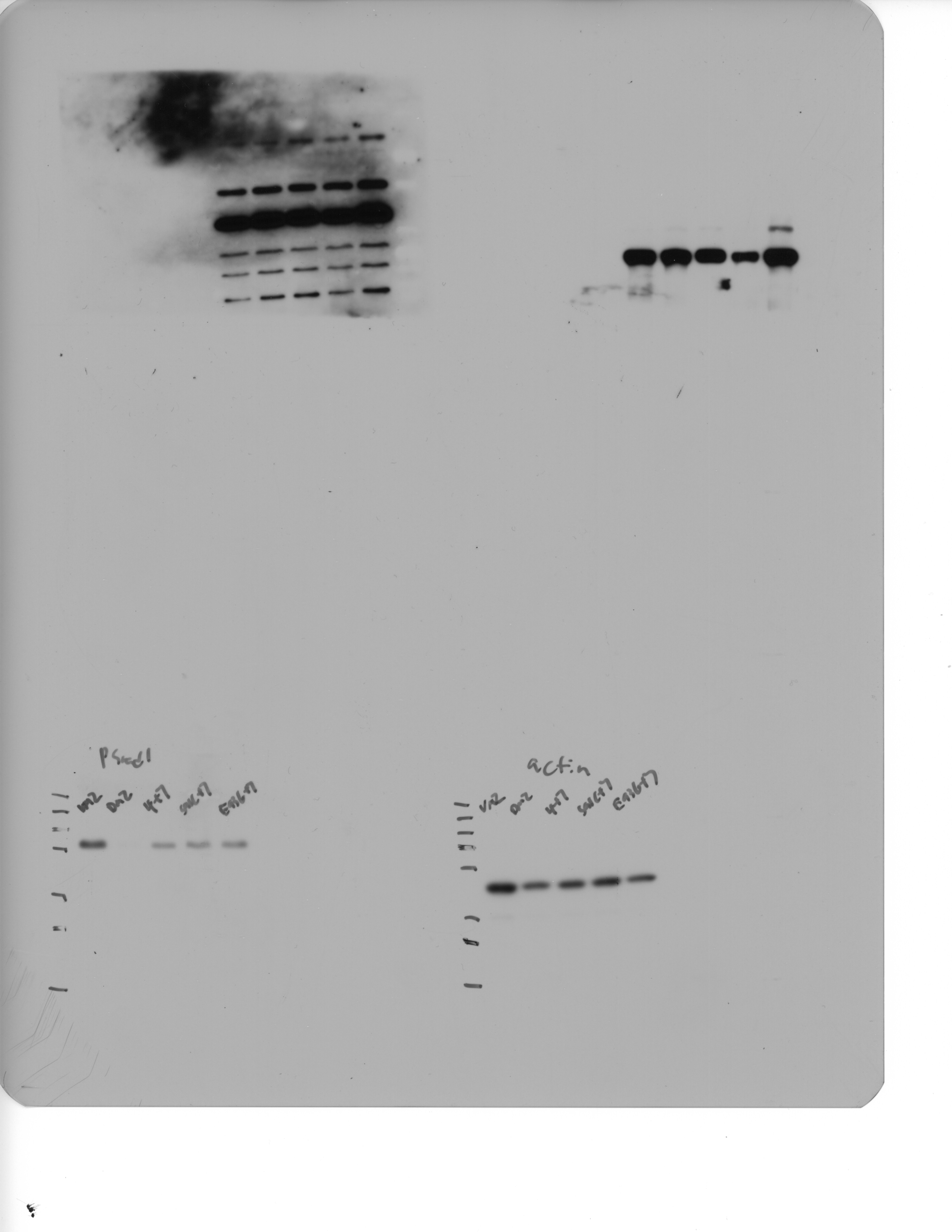

Supplement: Figure 1—source data 2. [file elife-105018-fig1-data2.zip › Figure 1-source data 2/Fig. 1D psmad actinHeterodimers copy.jpg]

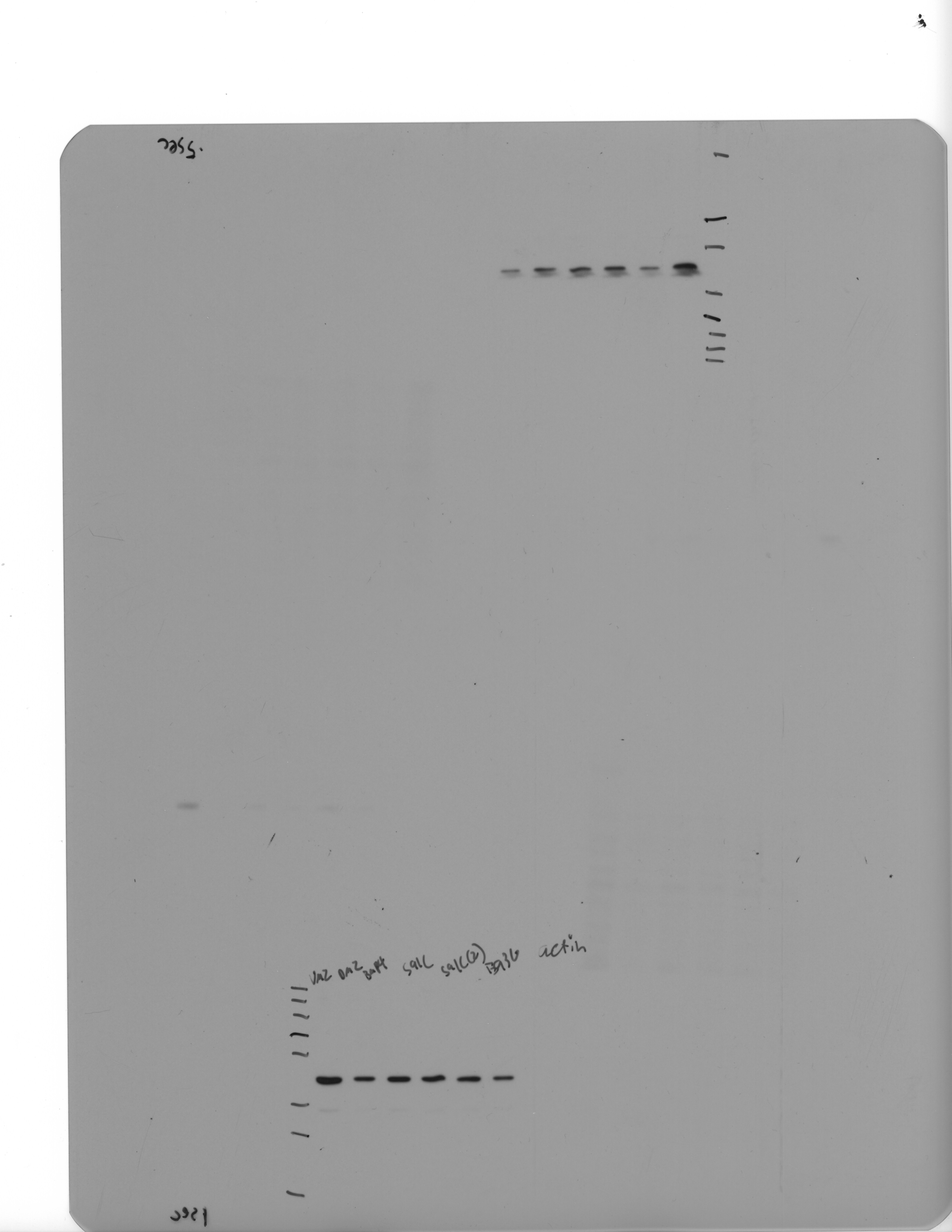

Supplement: Figure 1—source data 2. [file elife-105018-fig1-data2.zip › Figure 1-source data 2/Fig. 1D original homodimer blot actin.tif]

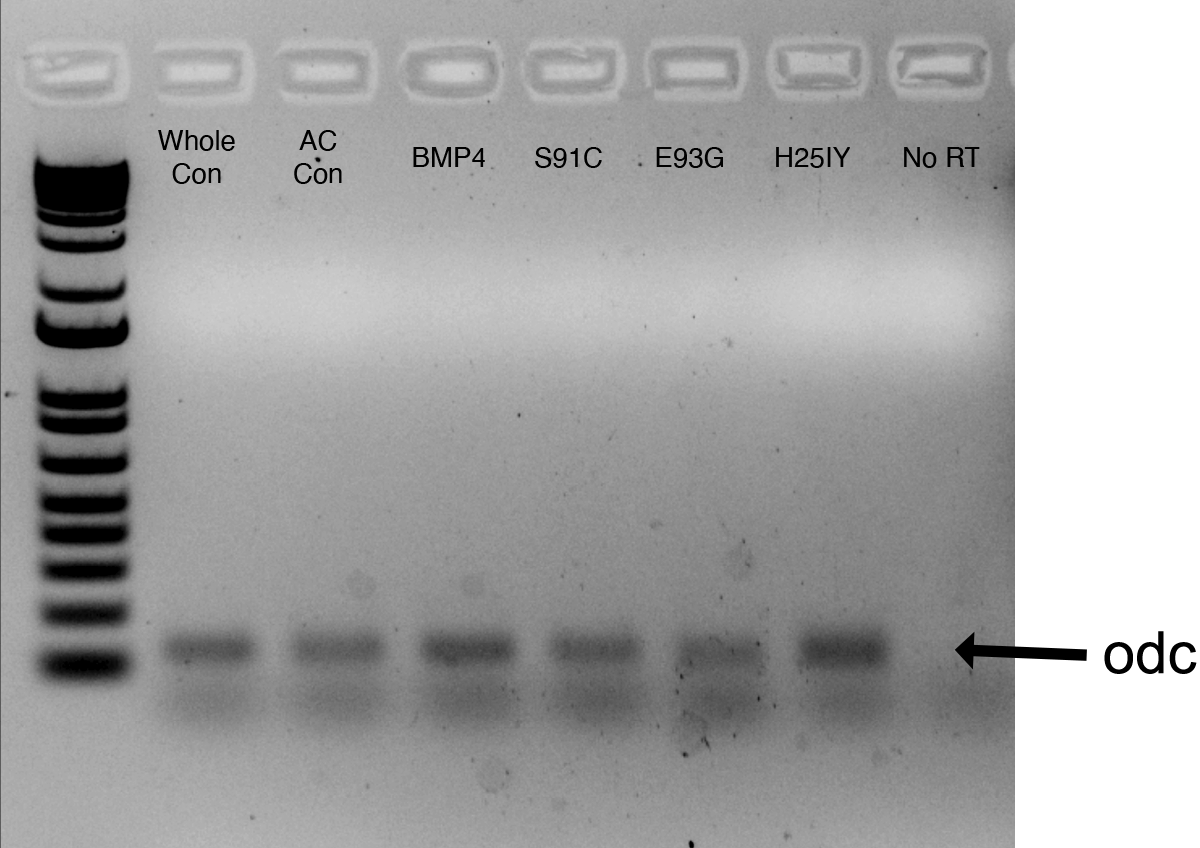

Supplement: Figure 1—source data 3. [file elife-105018-fig1-data3.zip › Figure 1-source data 3/ Fig. 1G 2021-12-03_homodimer ODC labeled .tif]

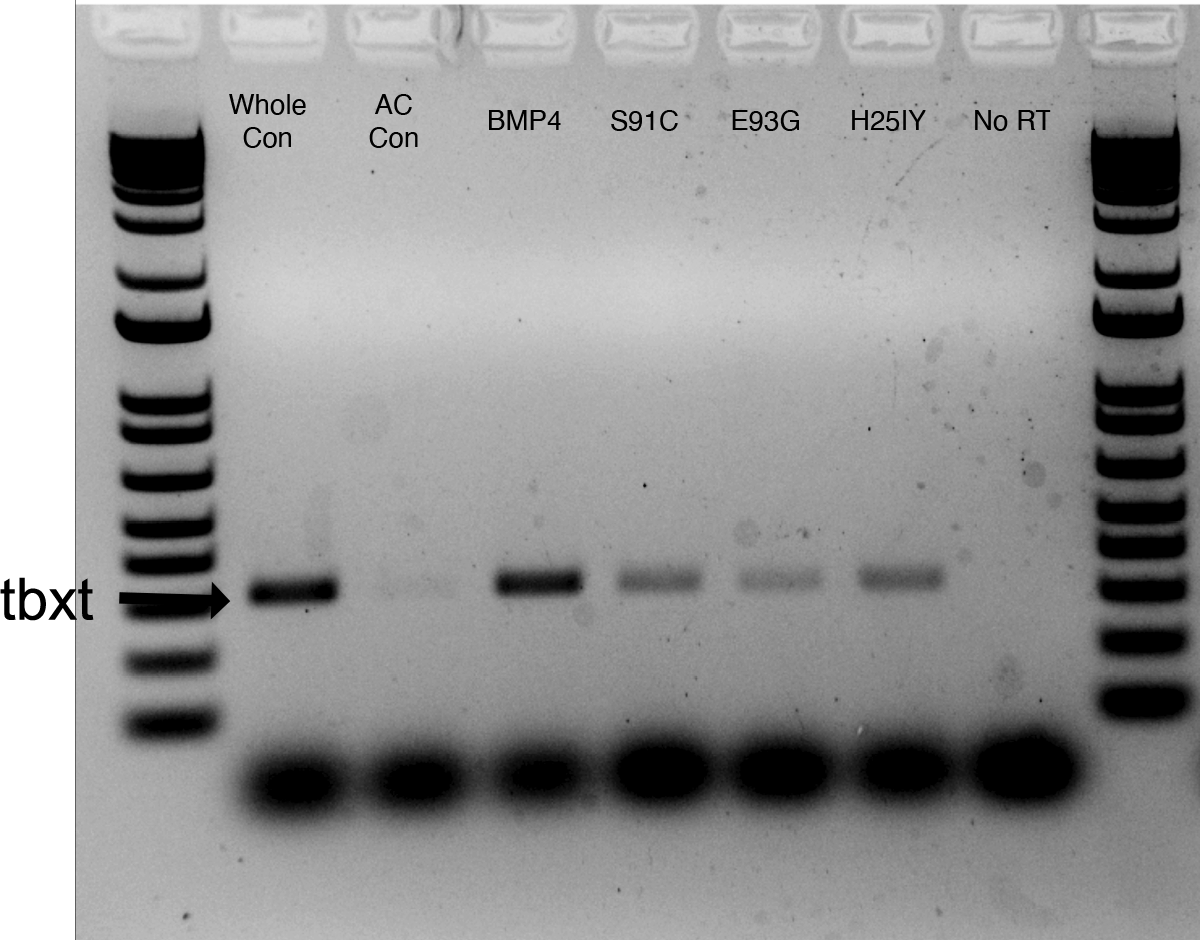

Supplement: Figure 1—source data 3. [file elife-105018-fig1-data3.zip › Figure 1-source data 3/Fig. 1G. 2021-12-02_ homo tbxt labeled.tif]

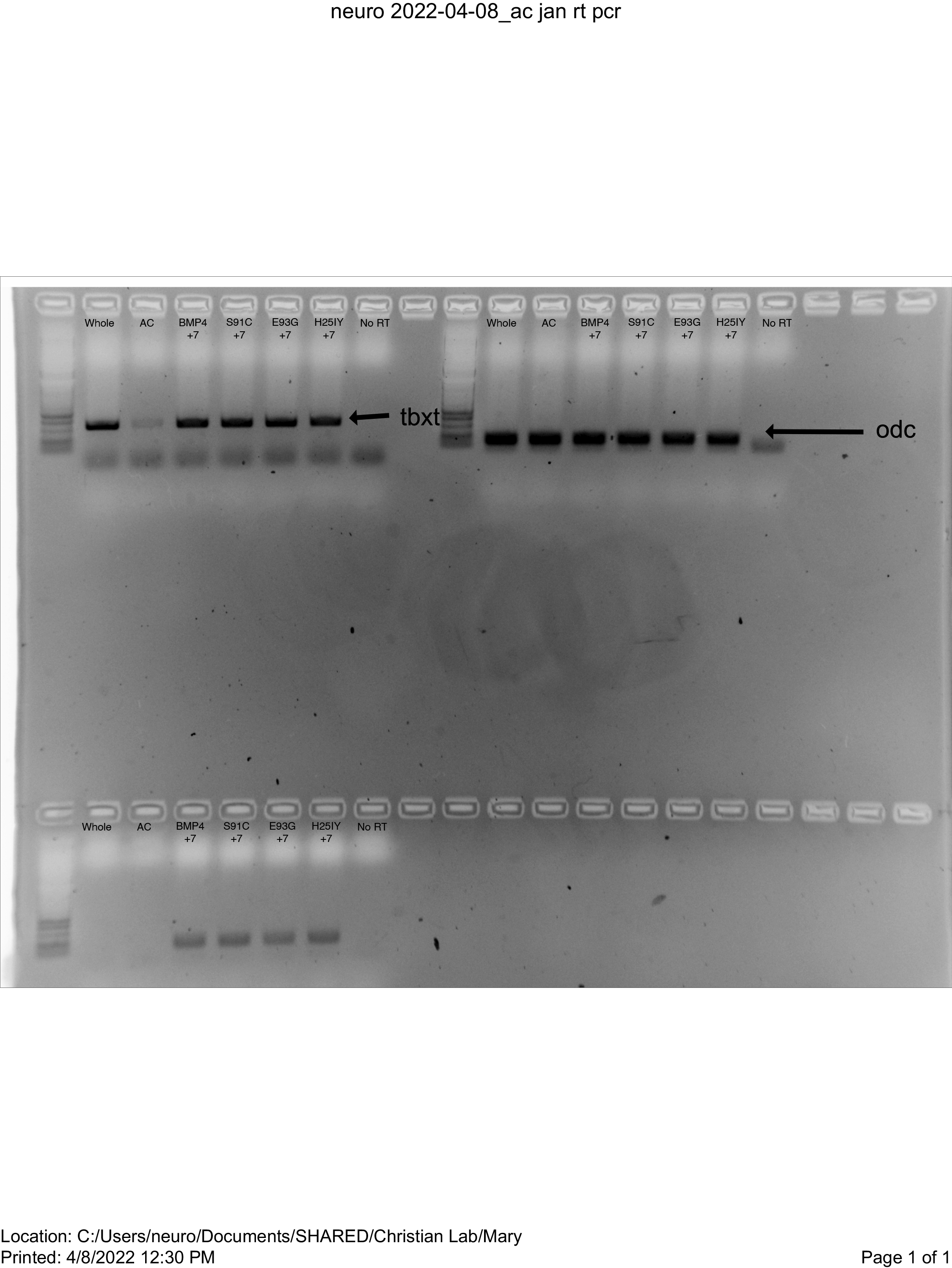

Supplement: Figure 1—source data 3. [file elife-105018-fig1-data3.zip › Figure 1-source data 3/Fig. 1G 2022-04-08_heterodimer PCR-bands marked.tif]

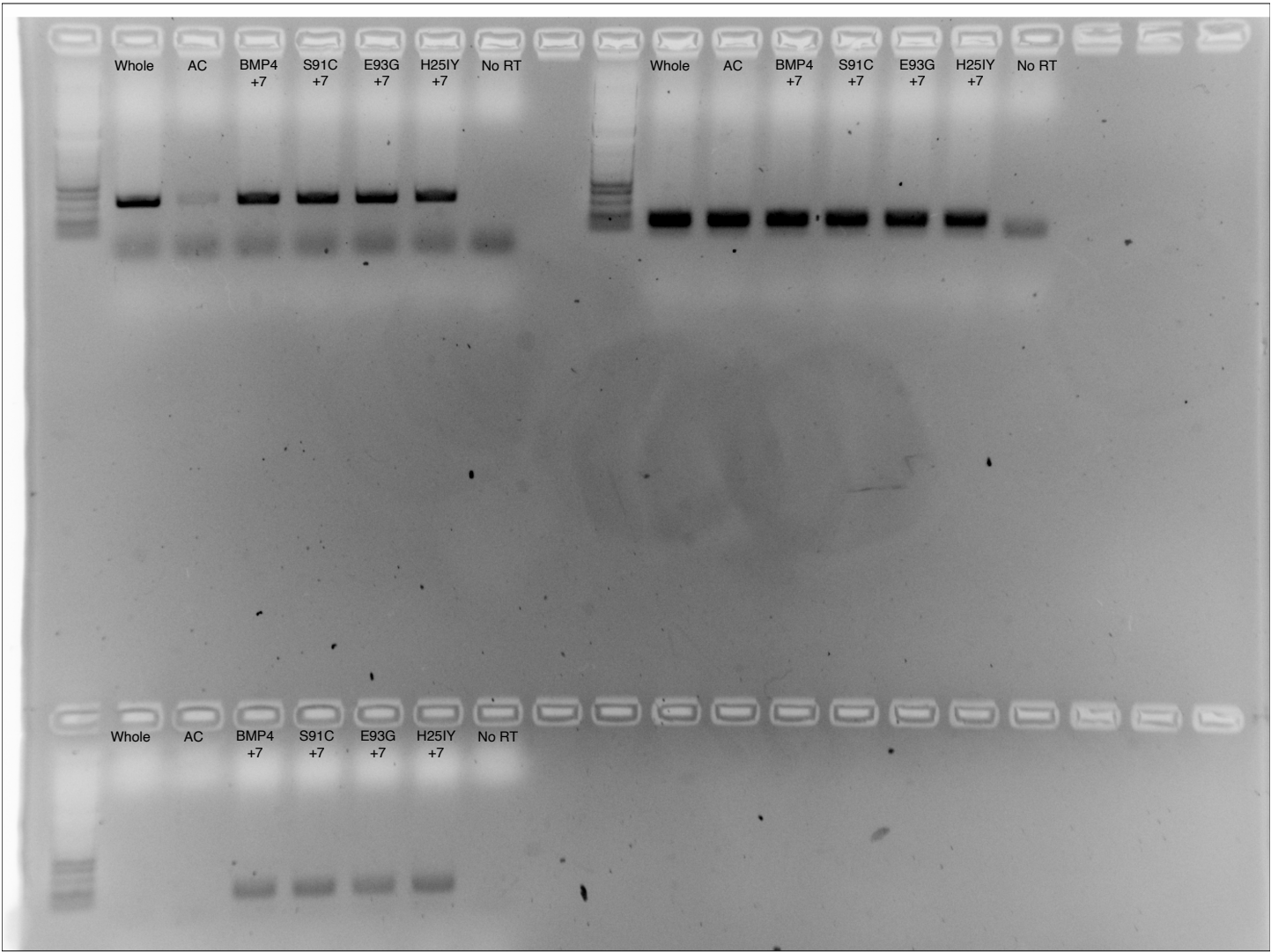

Supplement: Figure 1—source data 4. [file elife-105018-fig1-data4.zip › Figure 1. source data 4/Fig. 1G 2022-04-08_heterodimer PCR.pdf]

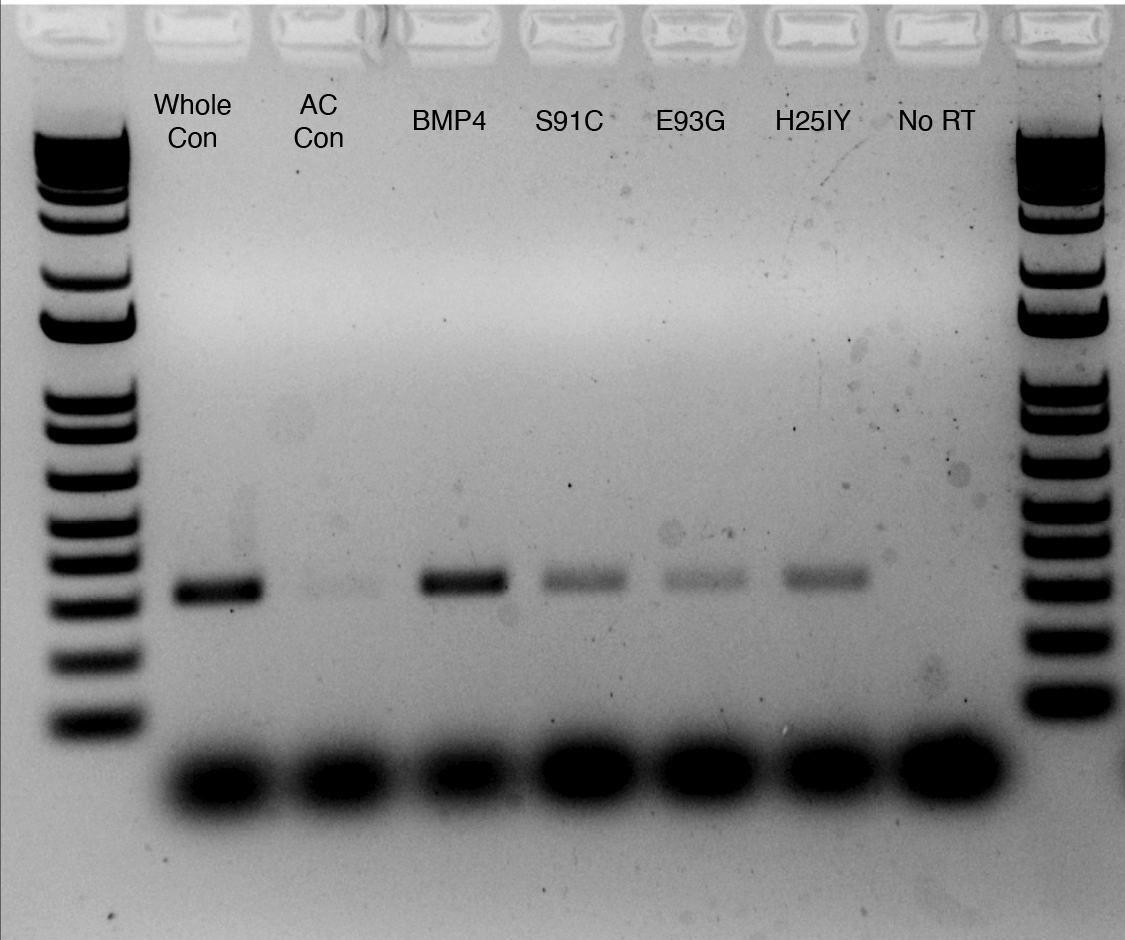

Supplement: Figure 1—source data 4. [file elife-105018-fig1-data4.zip › Figure 1. source data 4/Fig. 1G. 2021-12-02_ homo tbxt.tif]

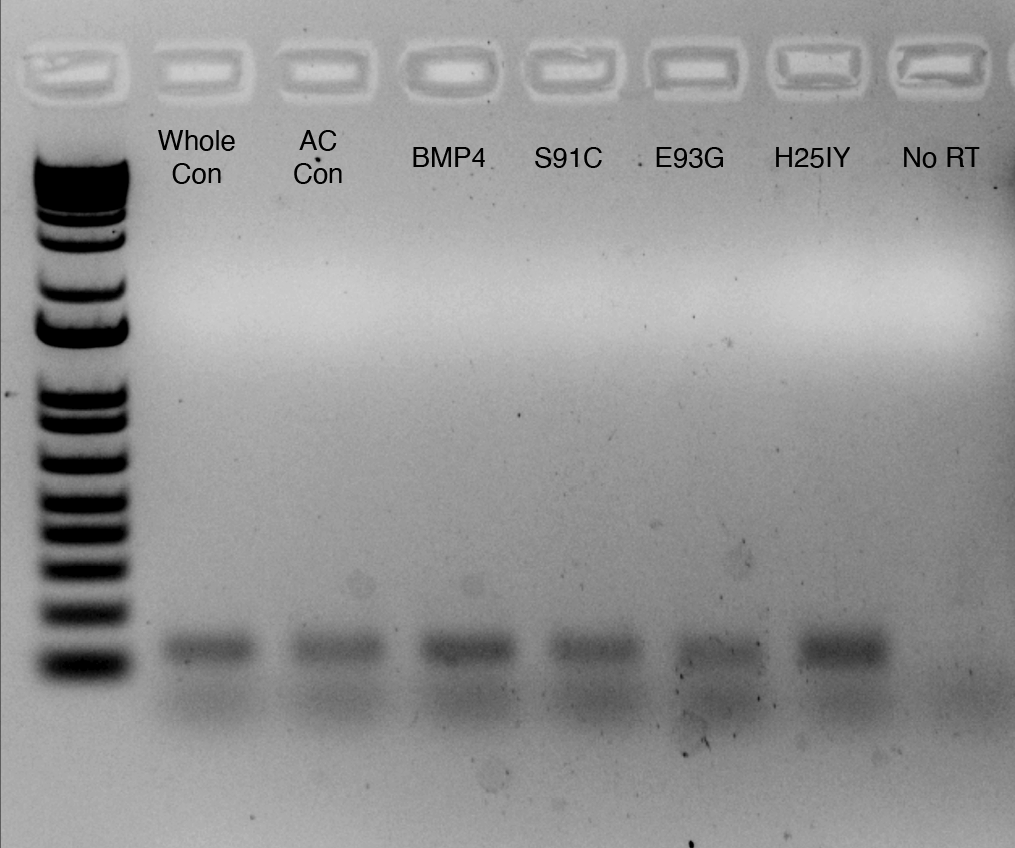

Supplement: Figure 1—source data 4. [file elife-105018-fig1-data4.zip › Figure 1. source data 4/ Fig. 1G 2021-12-03_homodimer ODC .tif]

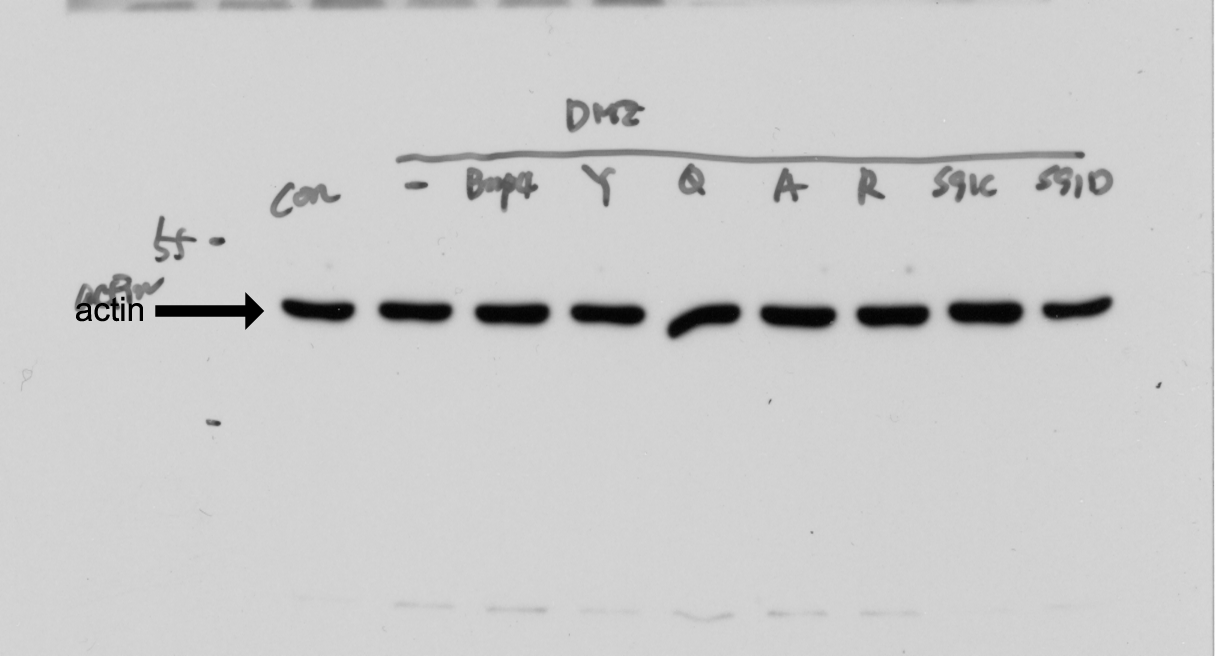

Supplement: Figure 1—figure supplement 1—source data 1. [file elife-105018-fig1-figsupp1-data1.zip › Figure 1-figure supplement 1-source data 1/Fig. 1-figure supplement 1A actiin marked.tif]

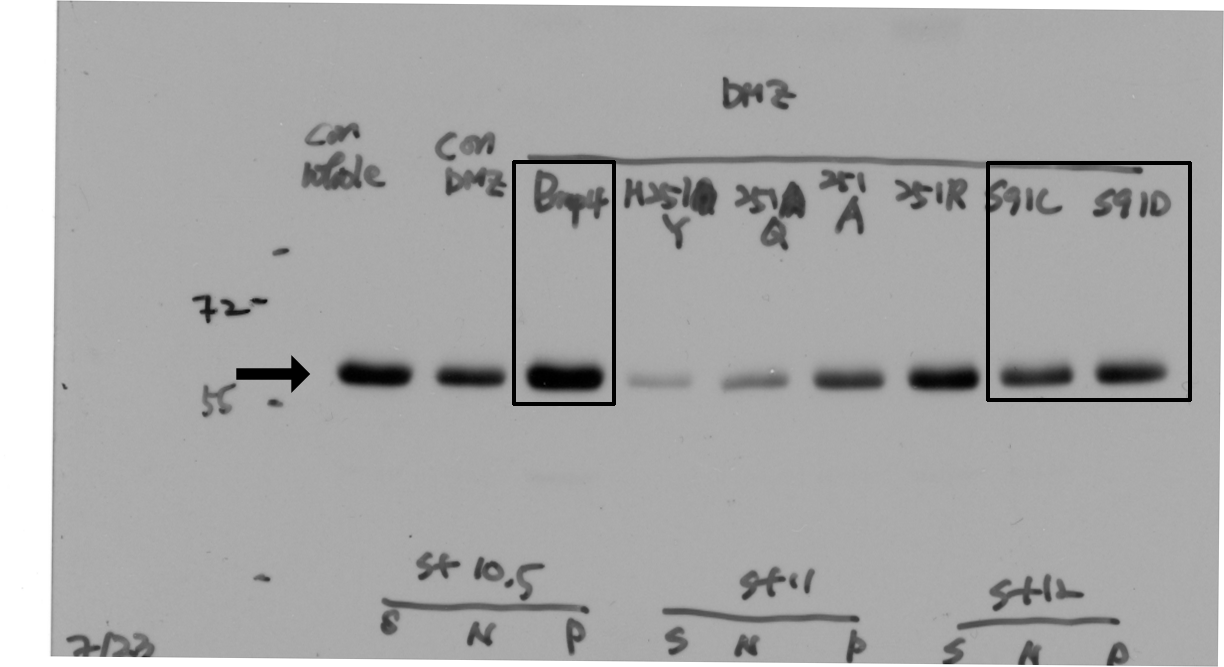

Supplement: Figure 1—figure supplement 1—source data 1. [file elife-105018-fig1-figsupp1-data1.zip › Figure 1-figure supplement 1-source data 1/Fig. 1 Figure Supplement 1A pSmad1 marked.tif]

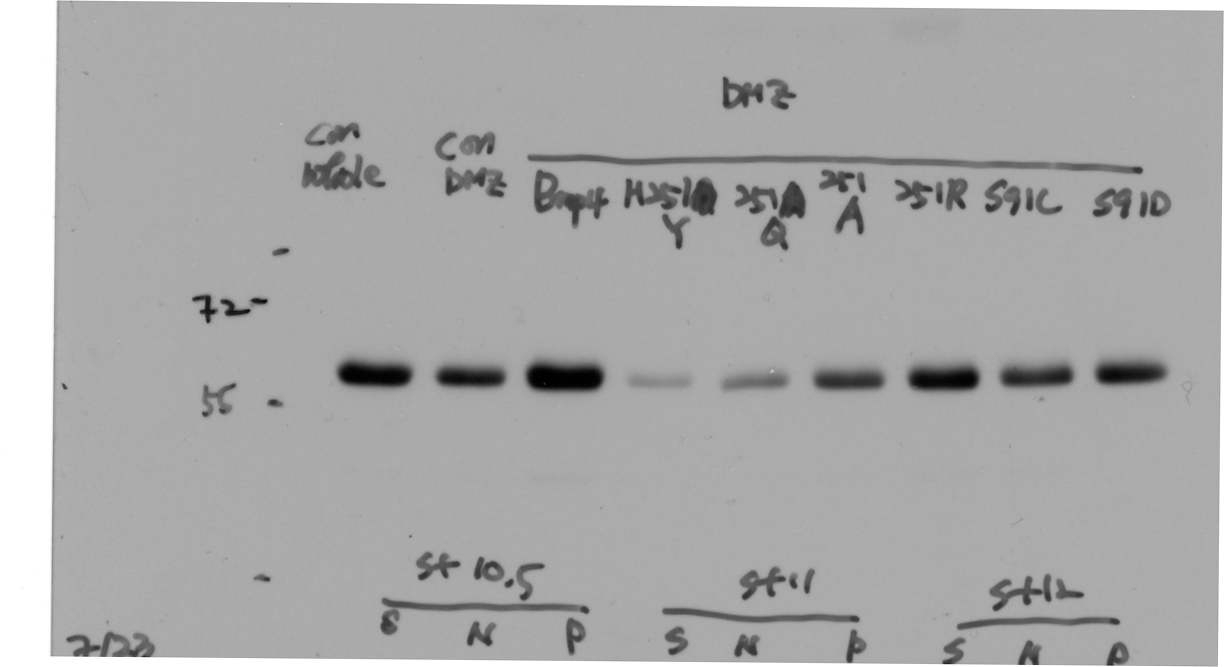

Supplement: Figure 1—figure supplement 1—source data 2. [file elife-105018-fig1-figsupp1-data2.zip › Figure 1-figure supplement 1- Source data/Fig. 1-figure supplement 1A unmarked.tif]

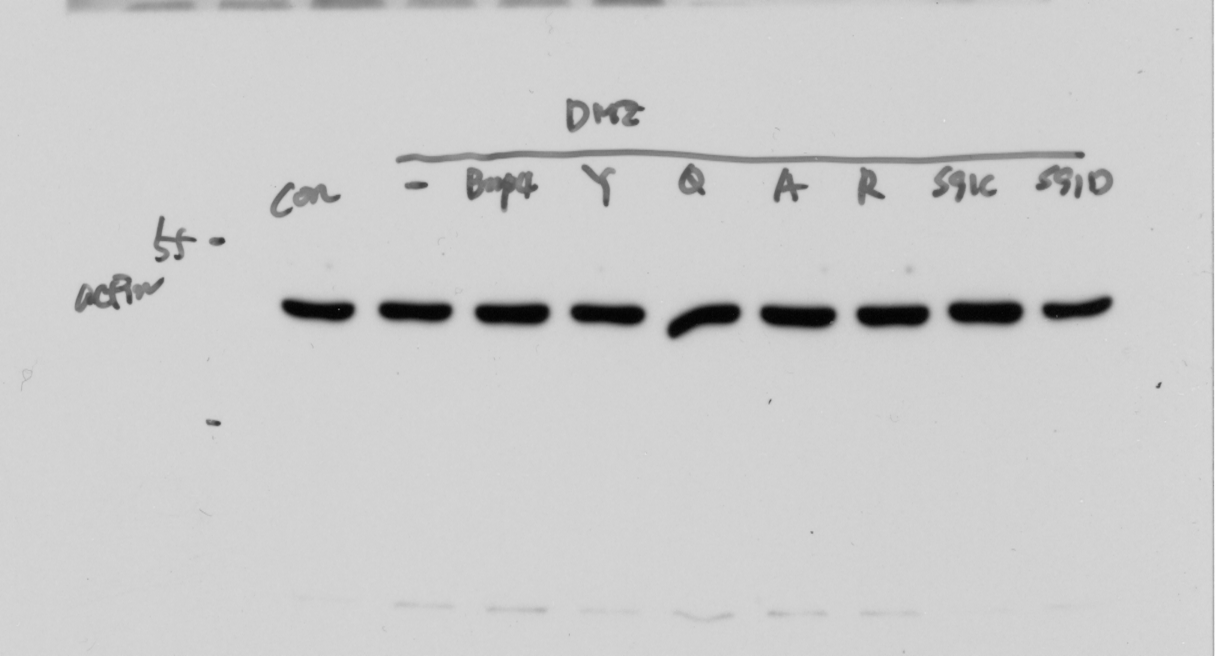

Supplement: Figure 1—figure supplement 1—source data 2. [file elife-105018-fig1-figsupp1-data2.zip › Figure 1-figure supplement 1- Source data/Fig. 1-figure supplement 1A actin unmarked.tif]

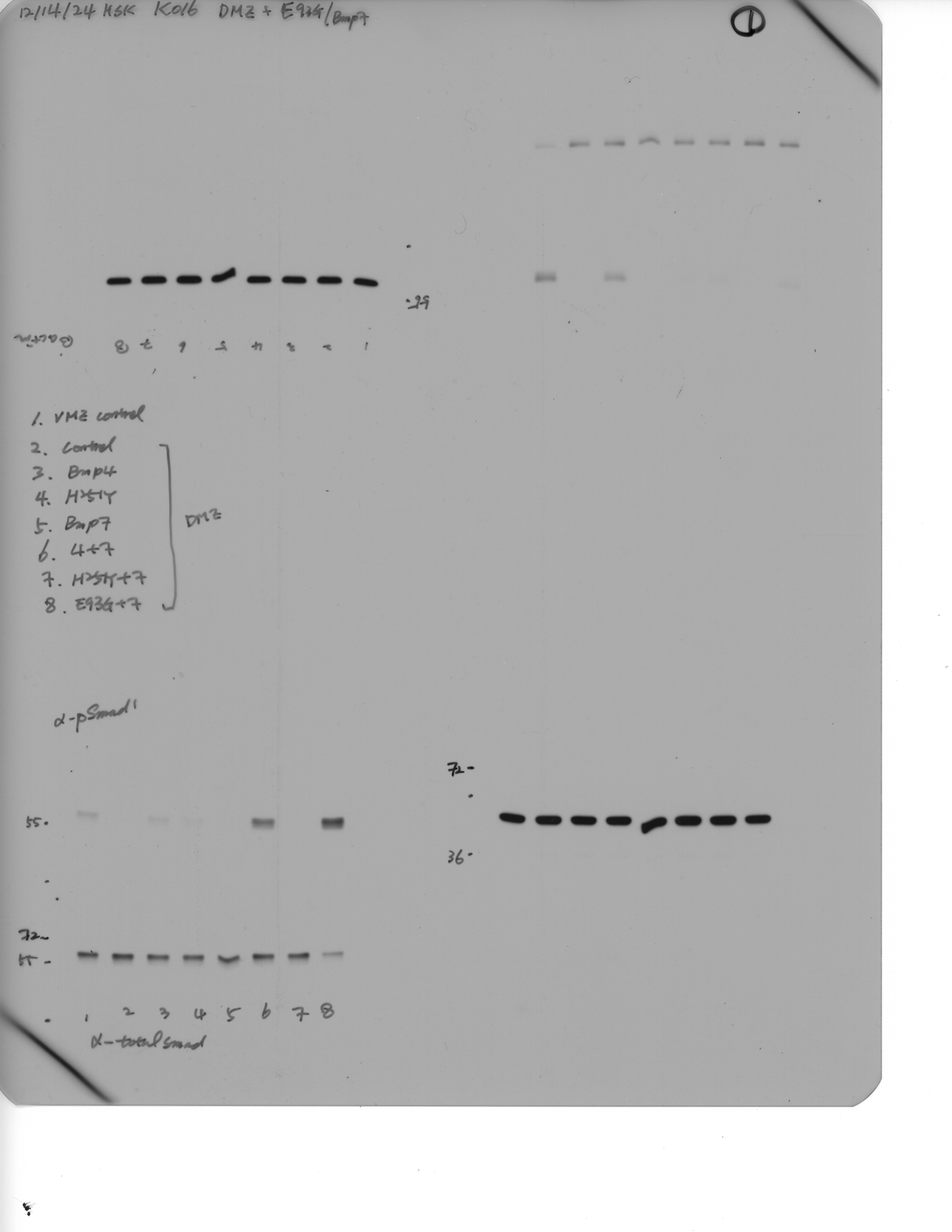

Supplement: Figure 1—figure supplement 2—source data 2. [file elife-105018-fig1-figsupp2-data2.zip › Figure 1-figure supplement 2-source data 2/K016_001 copy.tif]

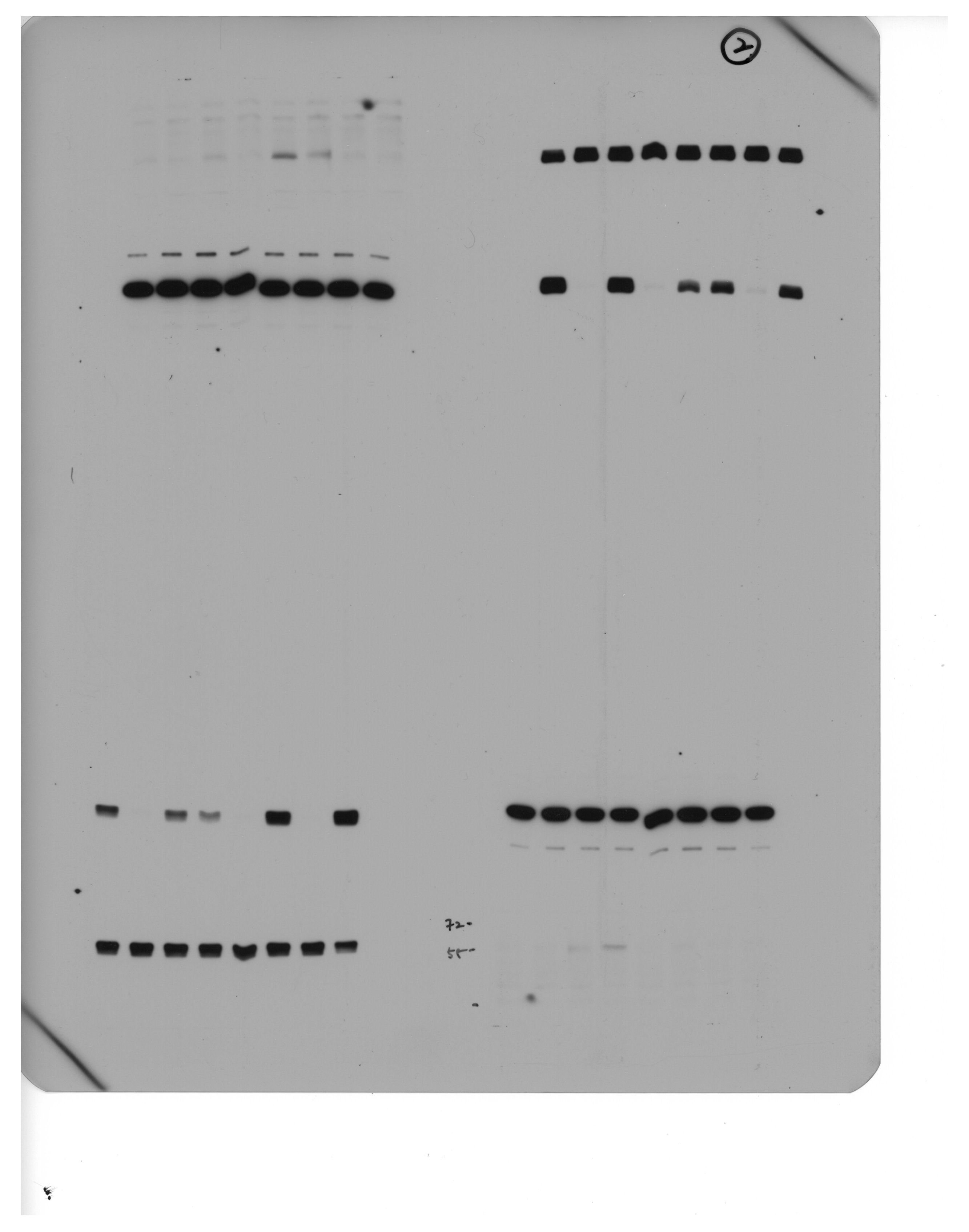

Supplement: Figure 1—figure supplement 2—source data 2. [file elife-105018-fig1-figsupp2-data2.zip › Figure 1-figure supplement 2-source data 2/K016_002 copy.tif]

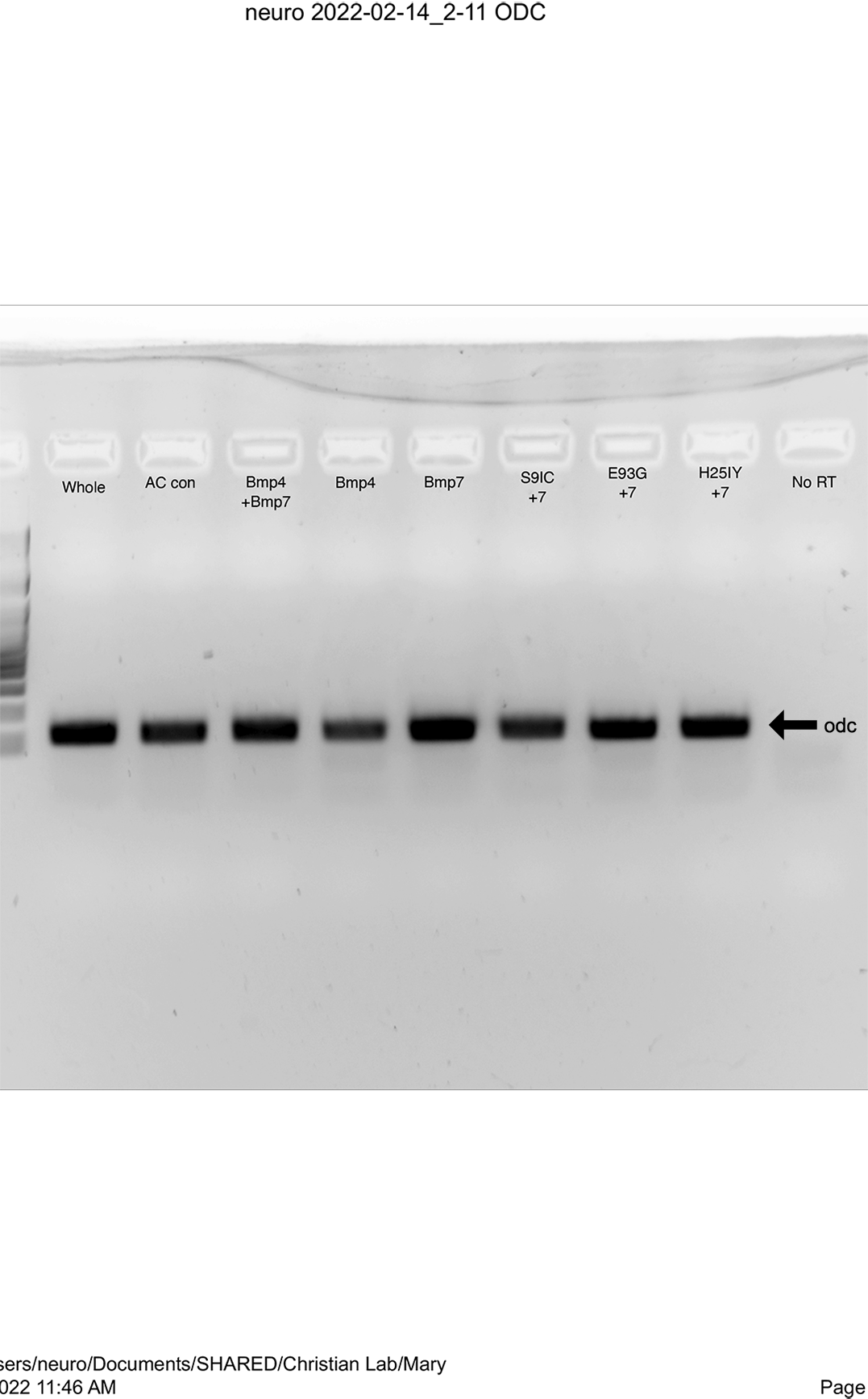

Supplement: Figure 1—figure supplement 2—source data 3. [file elife-105018-fig1-figsupp2-data3.zip › Figure 1-figure supplement 2 Source data 3/ Figure 1-figure supplement 2. ODC.tif]

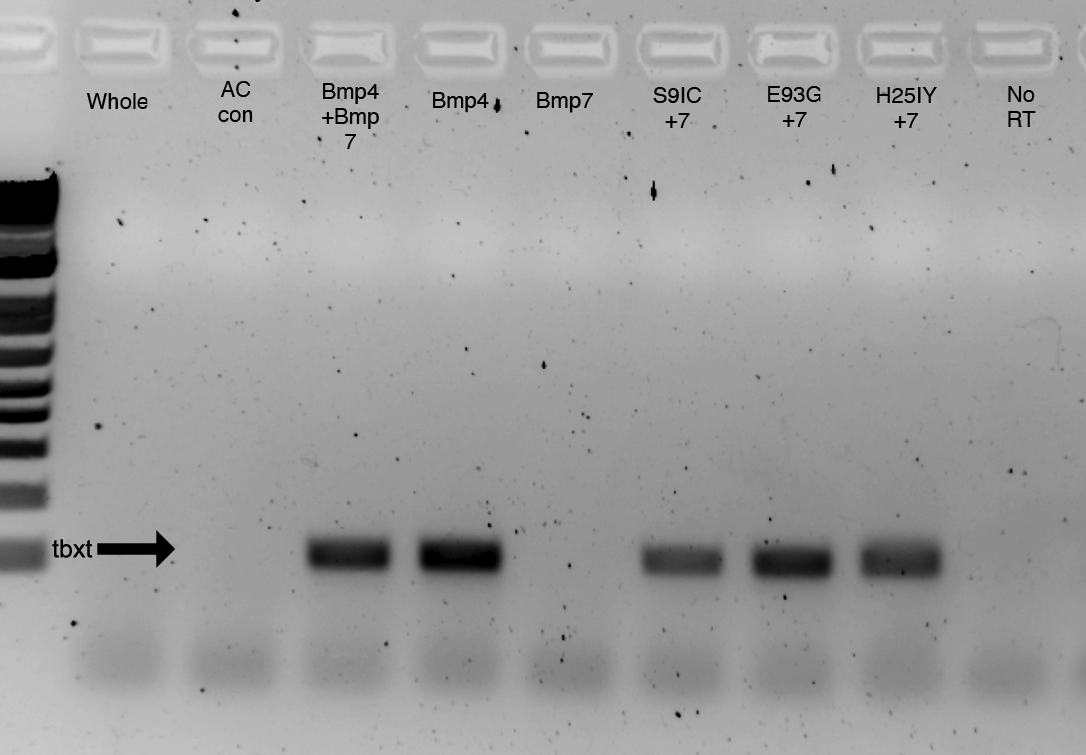

Supplement: Figure 1—figure supplement 2—source data 3. [file elife-105018-fig1-figsupp2-data3.zip › Figure 1-figure supplement 2 Source data 3/ Figure 1-figure supplement 2B_tbxt. .tif]

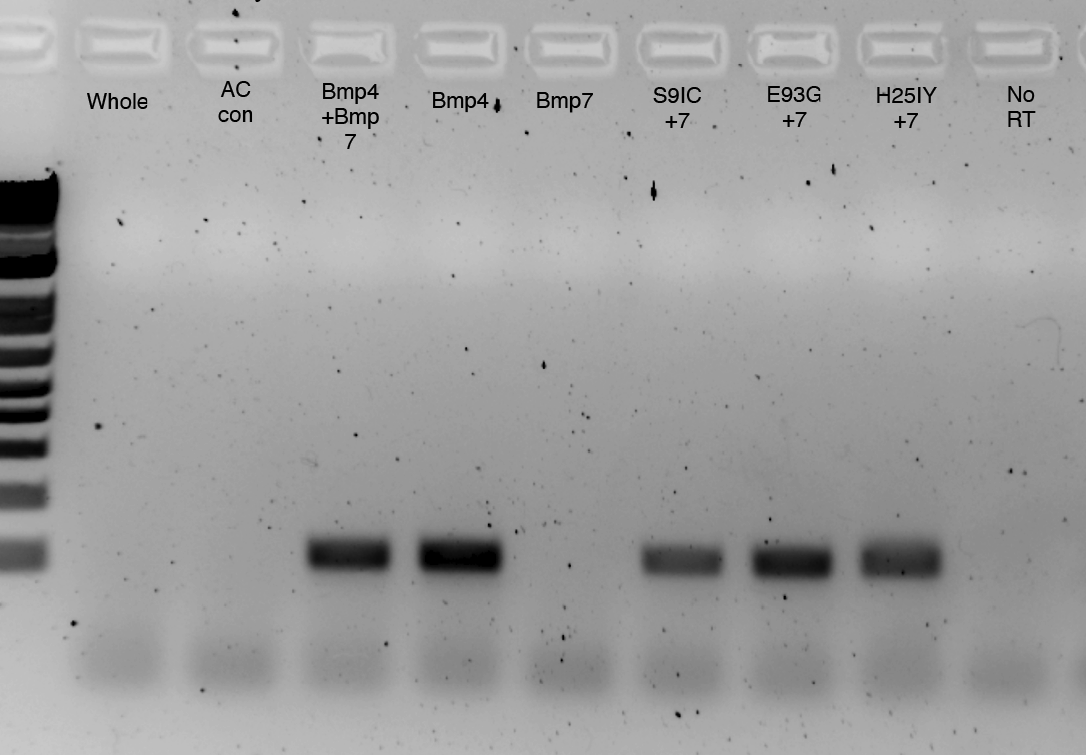

Supplement: Figure 1—figure supplement 2—source data 4. [file elife-105018-fig1-figsupp2-data4.zip › Figure 1-figure supplement 2-source data 4/neuro 2022-02-15_ 2-4 AND 2-11 BMP4 copy.tif]

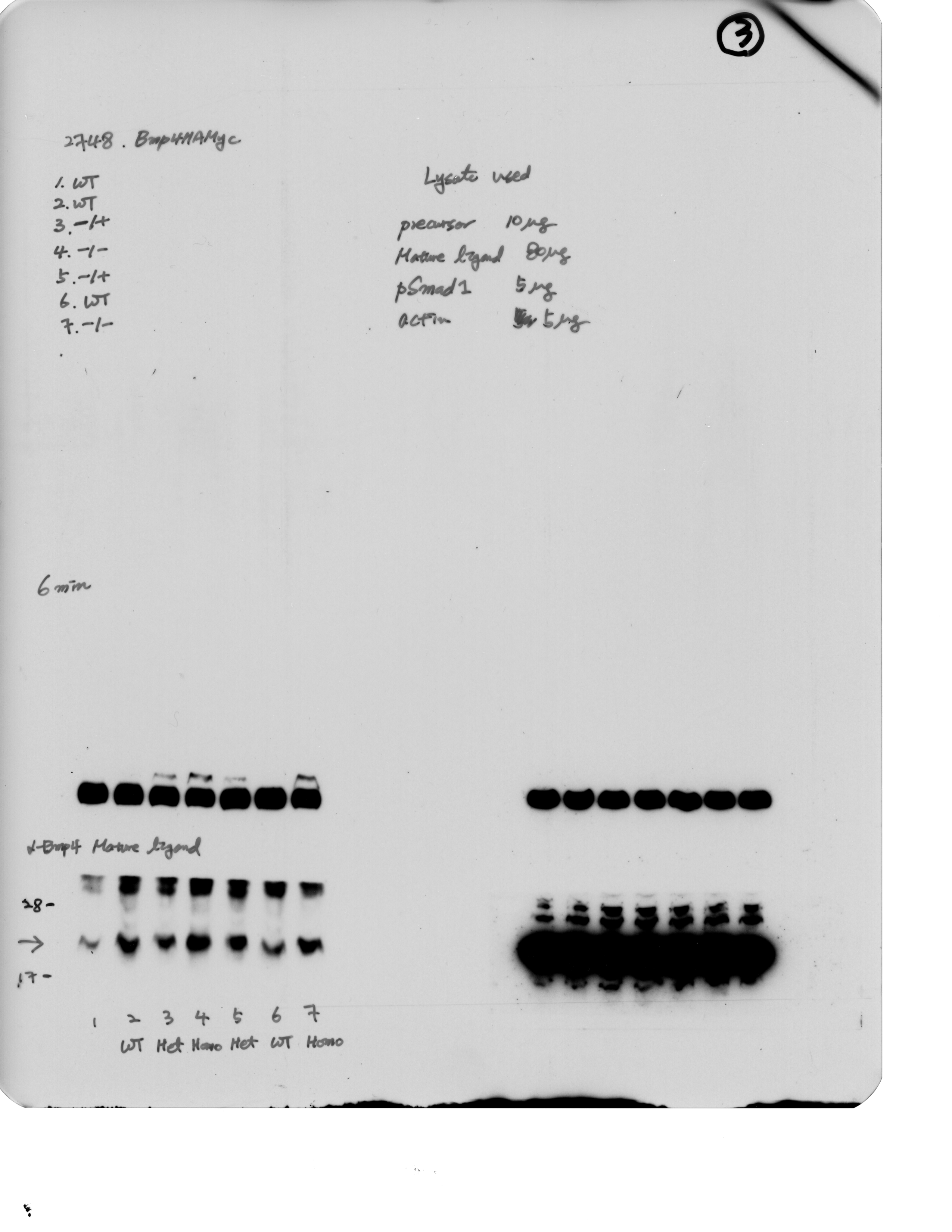

Supplement: Figure 4—source data 1. [file elife-105018-fig4-data1.zip › Figure 4-supplementary figure 1A Source data/Figure 4-supplementary figure 1ABMP4ligand unmarked.tif]

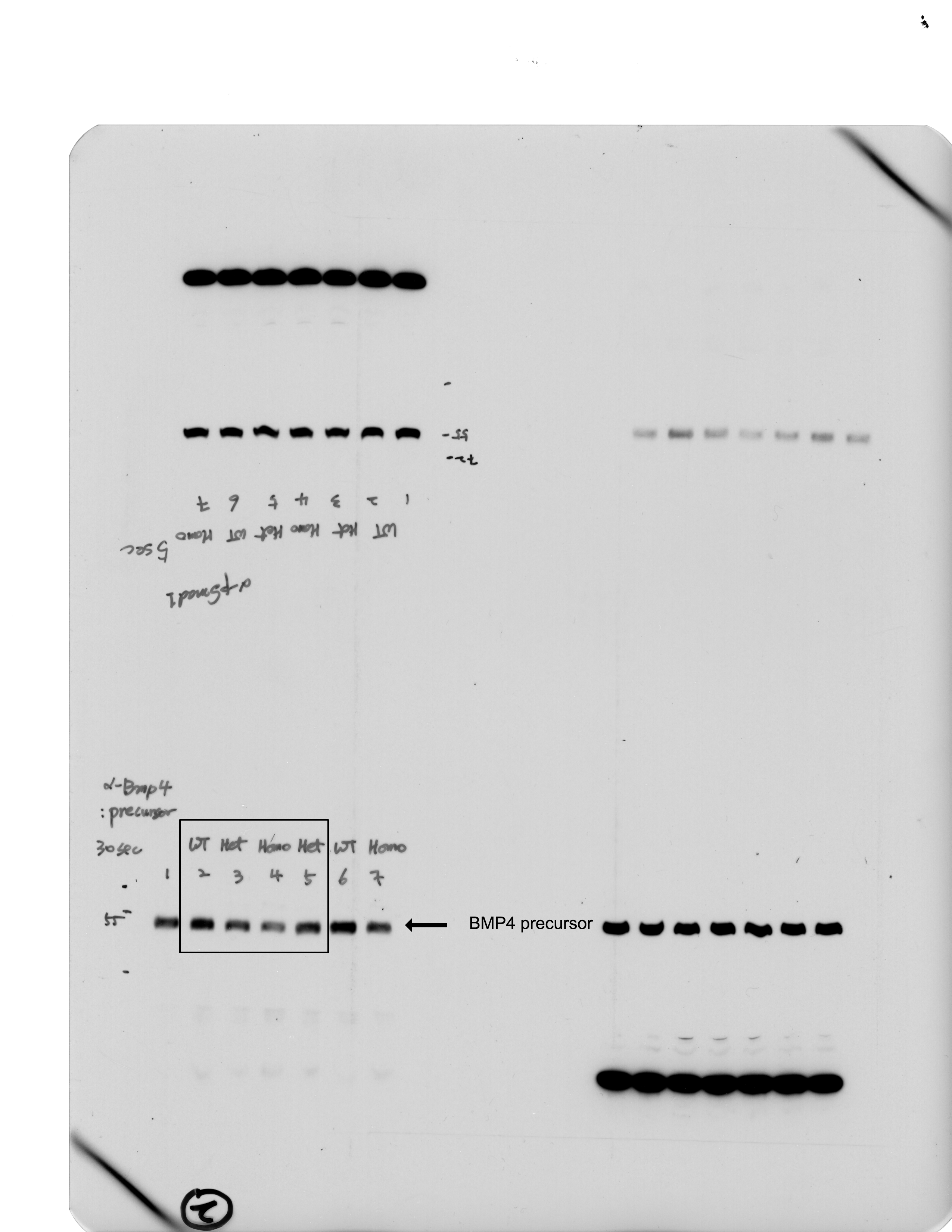

Supplement: Figure 4—source data 1. [file elife-105018-fig4-data1.zip › Figure 4-supplementary figure 1A Source data/Figure 4-supplementary figure 1Aprecursor marked.tif]

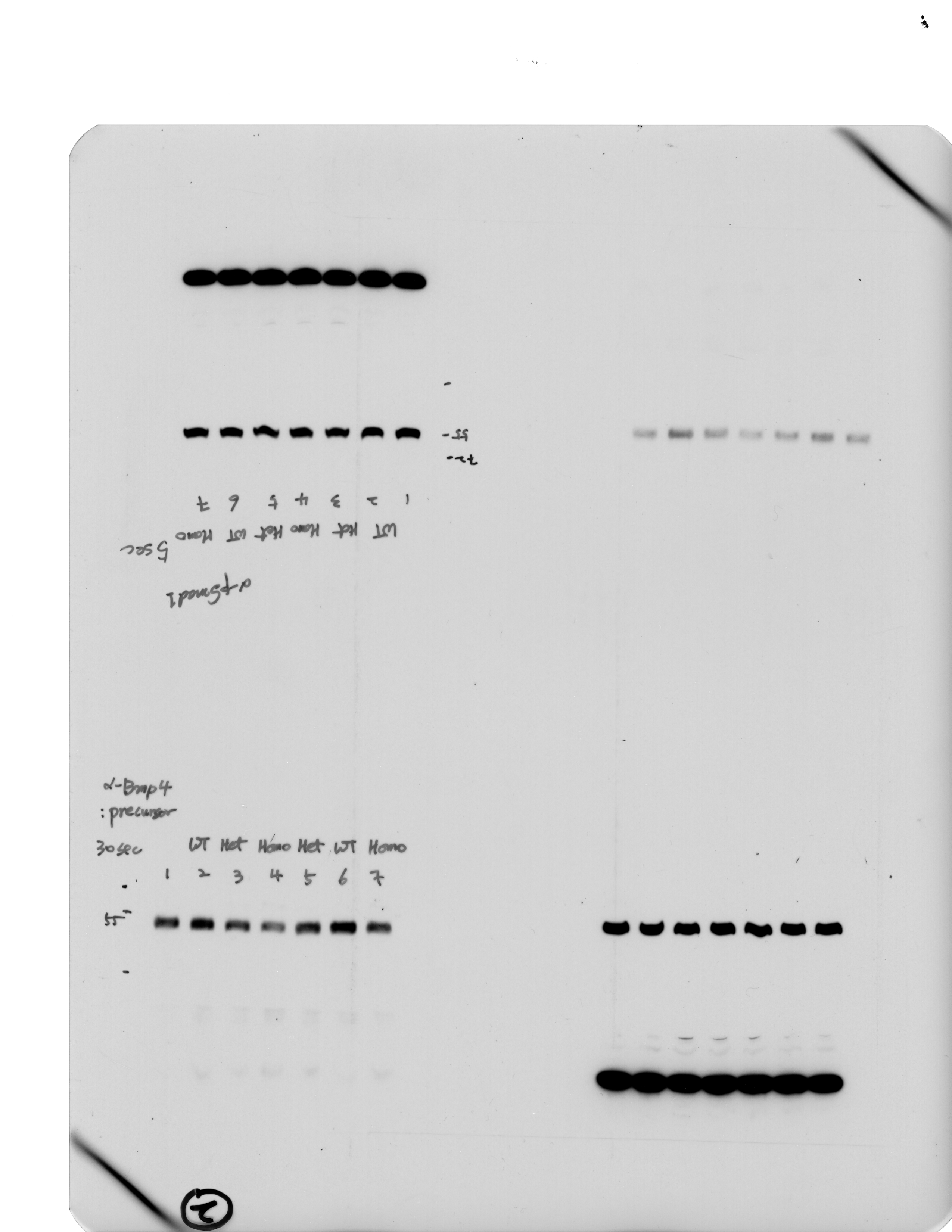

Supplement: Figure 4—source data 1. [file elife-105018-fig4-data1.zip › Figure 4-supplementary figure 1A Source data/Figure 4-supplementary figure 1Aprecursor unmarked.tif]

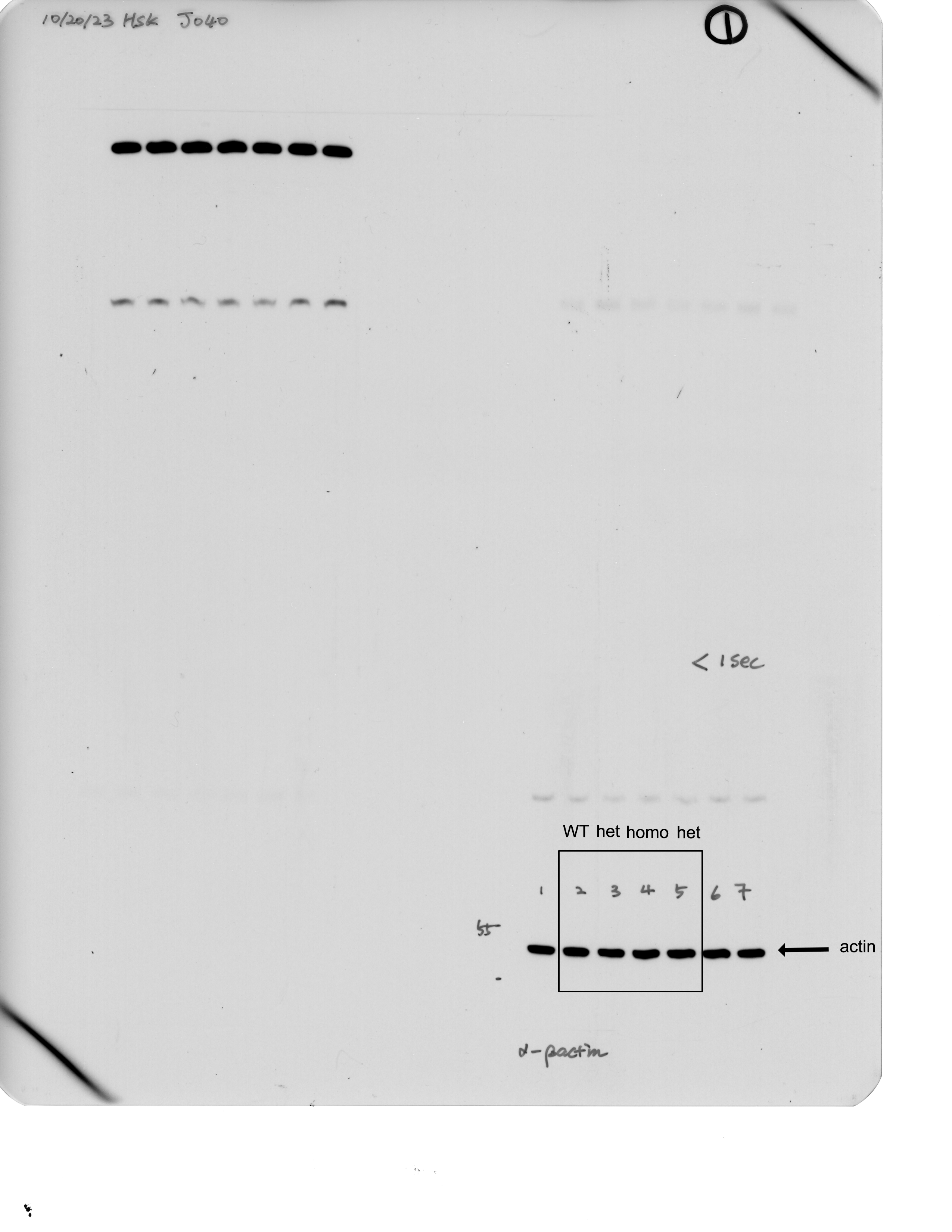

Supplement: Figure 4—source data 1. [file elife-105018-fig4-data1.zip › Figure 4-supplementary figure 1A Source data/Figure 4-supplementary figure 1Aactin marked.tif]

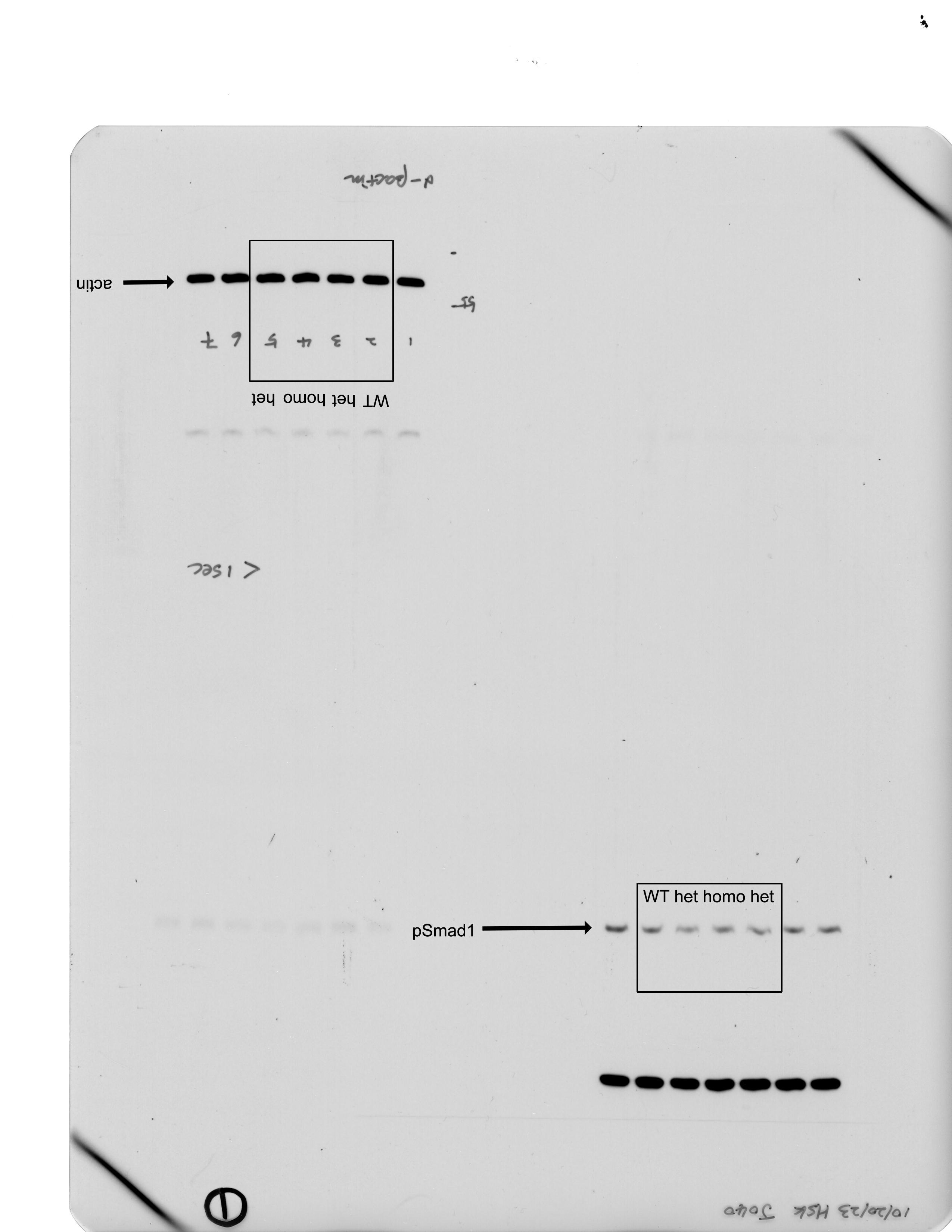

Supplement: Figure 4—source data 1. [file elife-105018-fig4-data1.zip › Figure 4-supplementary figure 1A Source data/Figure 4-supplementary figure 1Aa pSmad1 marked.tif]

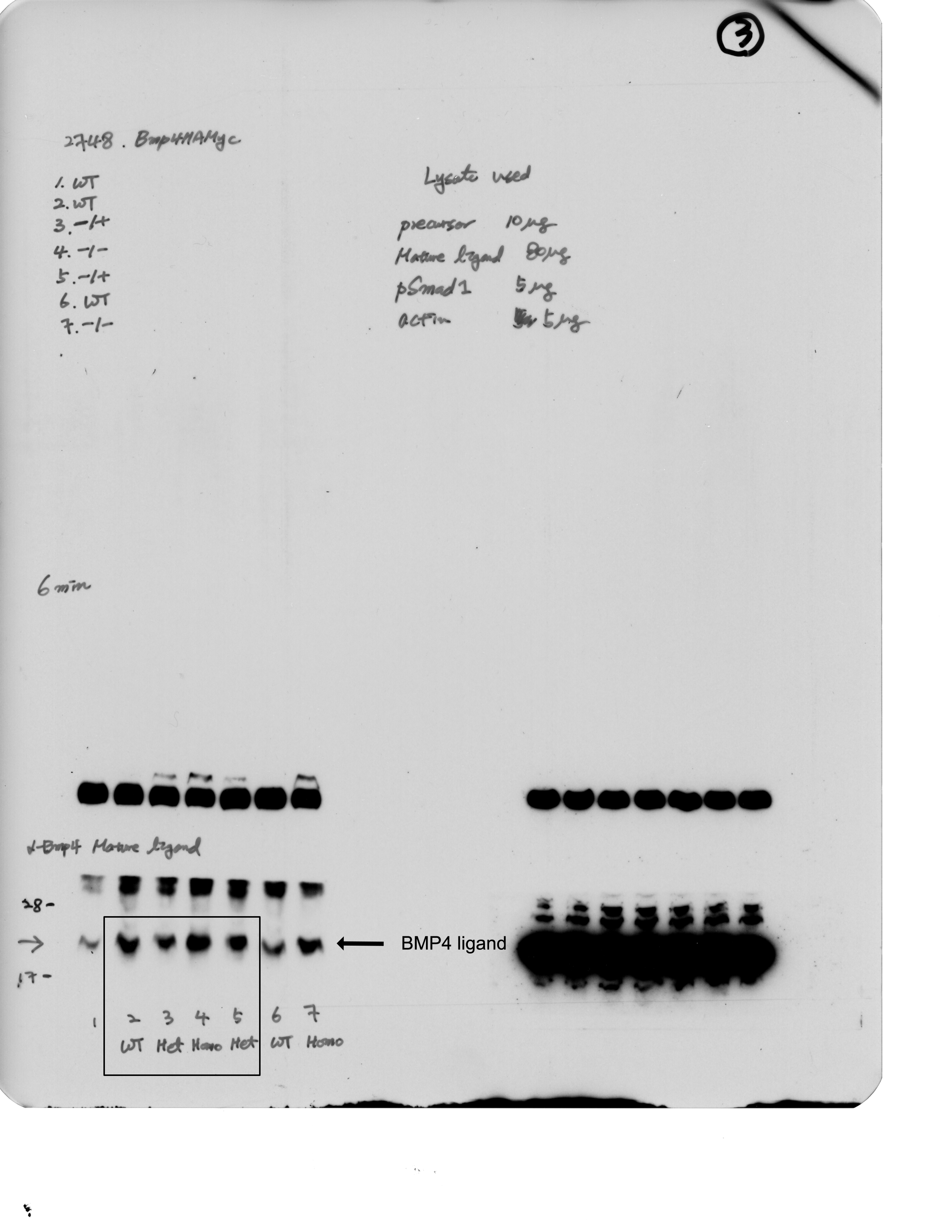

Supplement: Figure 4—source data 1. [file elife-105018-fig4-data1.zip › Figure 4-supplementary figure 1A Source data/Figure 4-supplementary figure 1ABMP4ligand marked.tif]

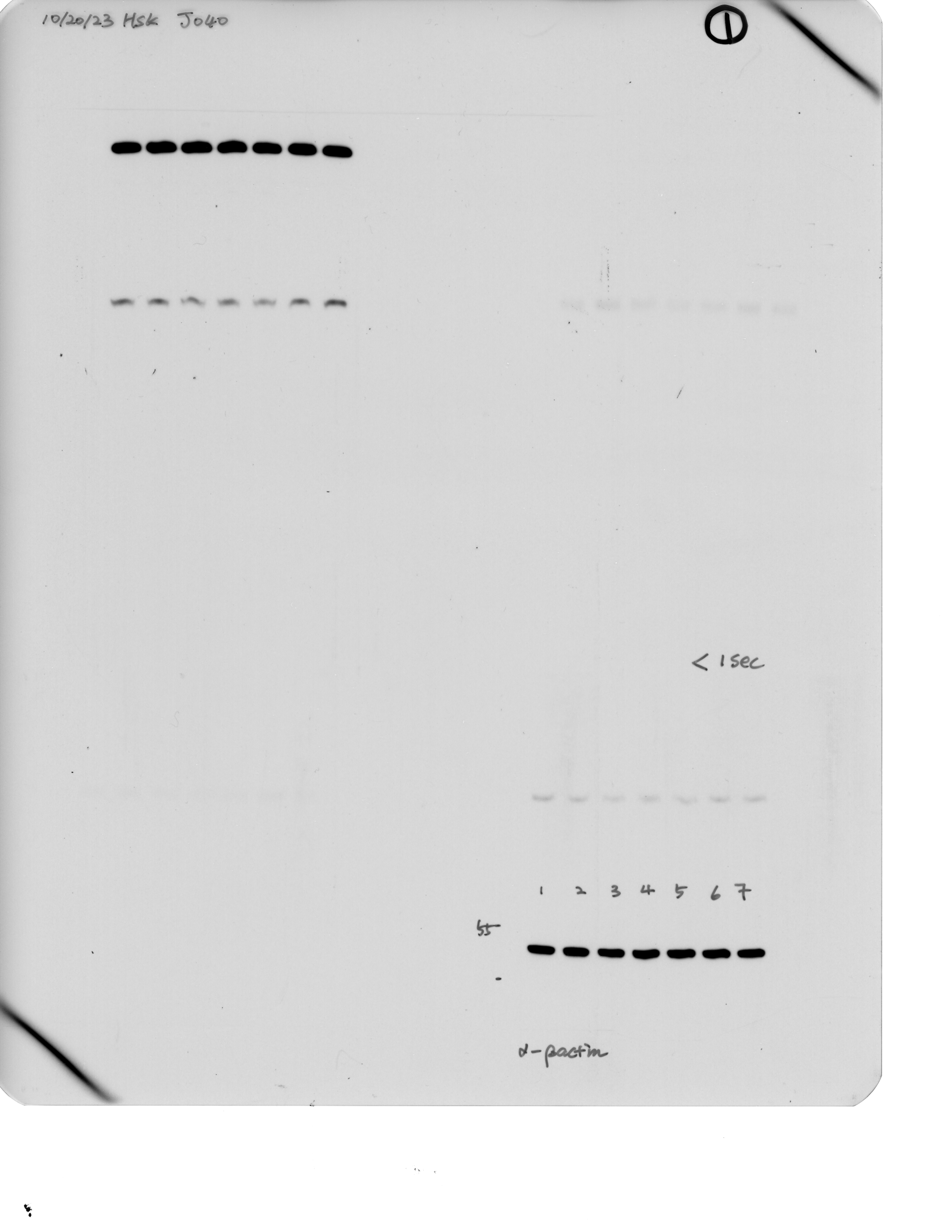

Supplement: Figure 4—source data 1. [file elife-105018-fig4-data1.zip › Figure 4-supplementary figure 1A Source data/Figure 4-supplementary figure 1Aactin unmarked.tif]

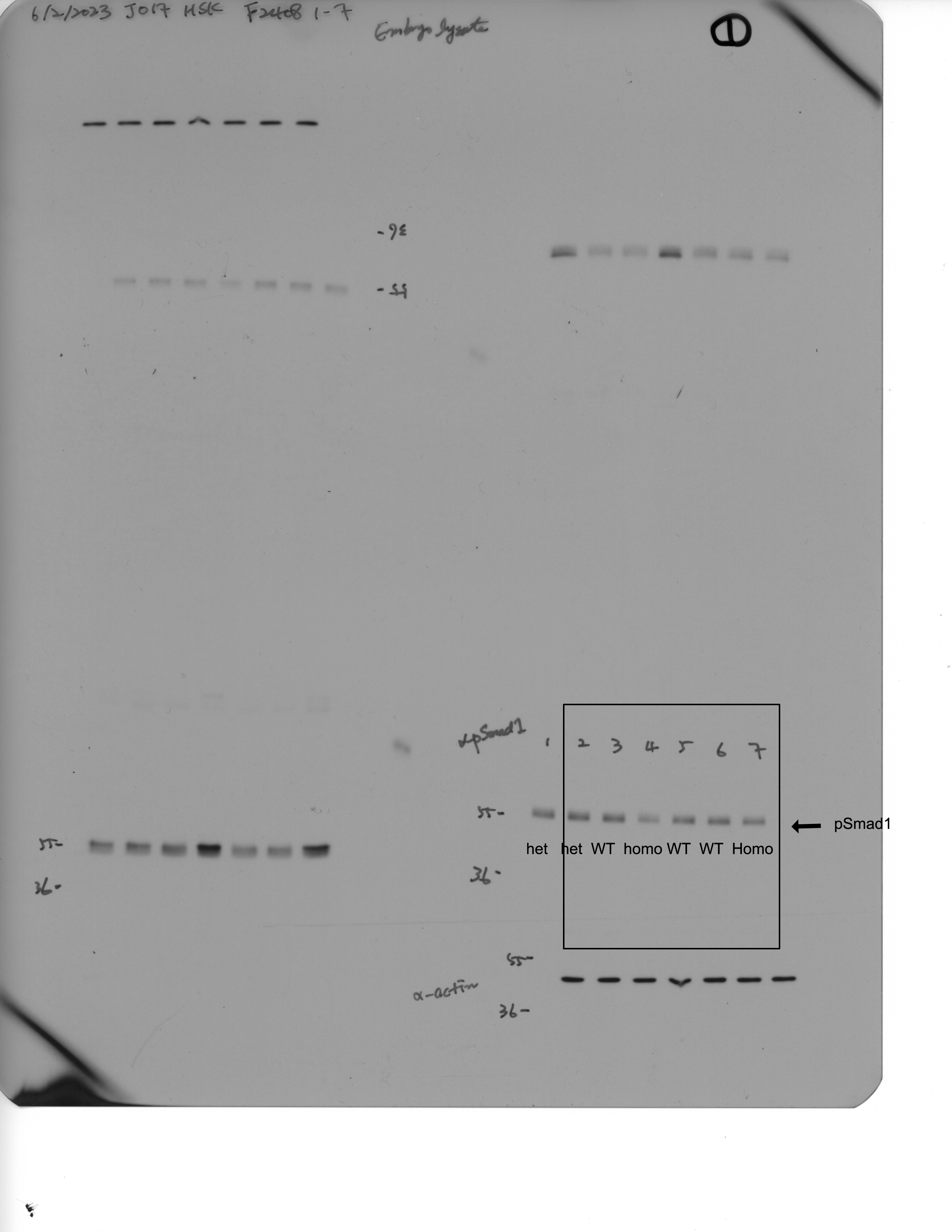

Supplement: Figure 6—source data 1. [file elife-105018-fig6-data1.zip › Figure 4-source data 1/J017_001 pSmad1 marked.tif]

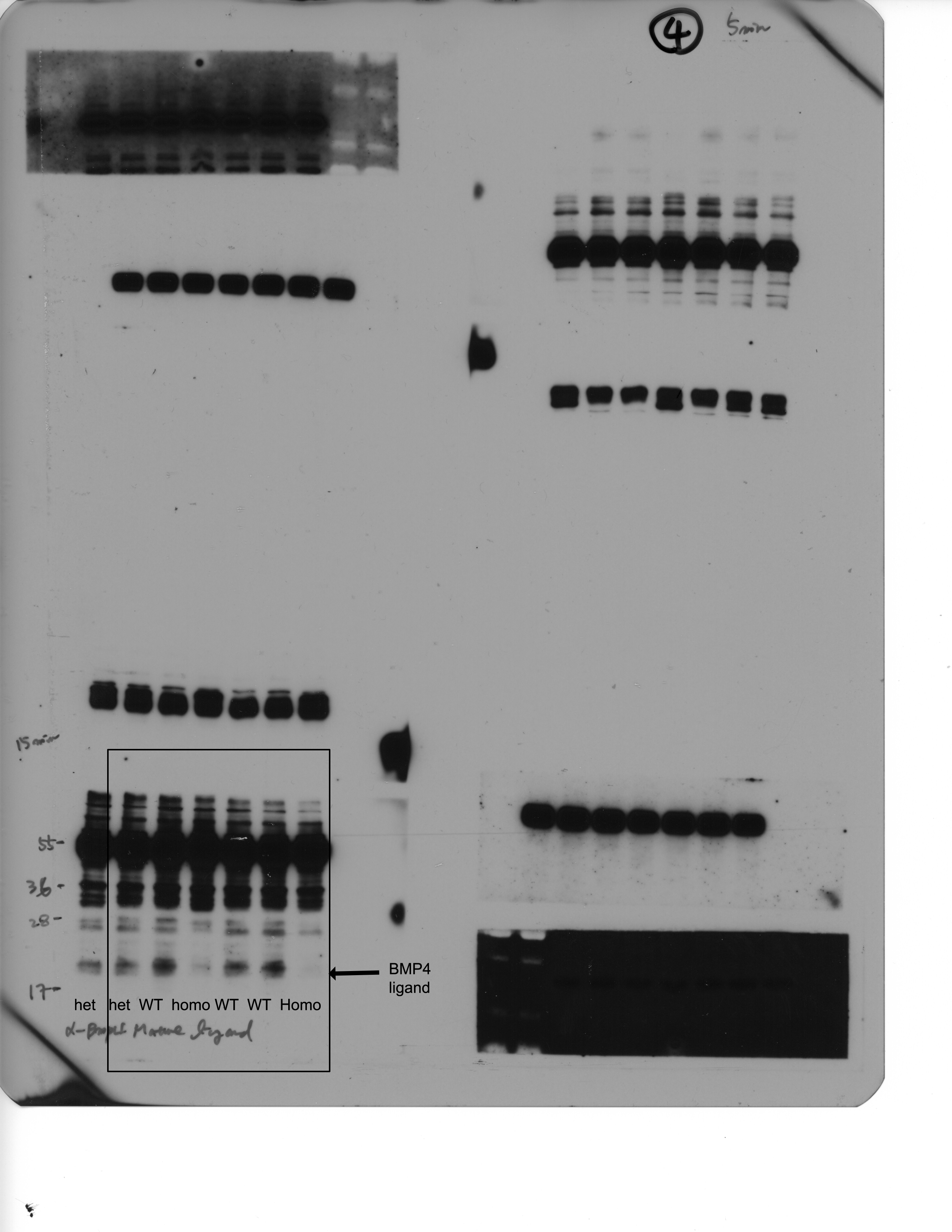

Supplement: Figure 6—source data 1. [file elife-105018-fig6-data1.zip › Figure 4-source data 1/J017_004 BMP4 ligand marked.tif]

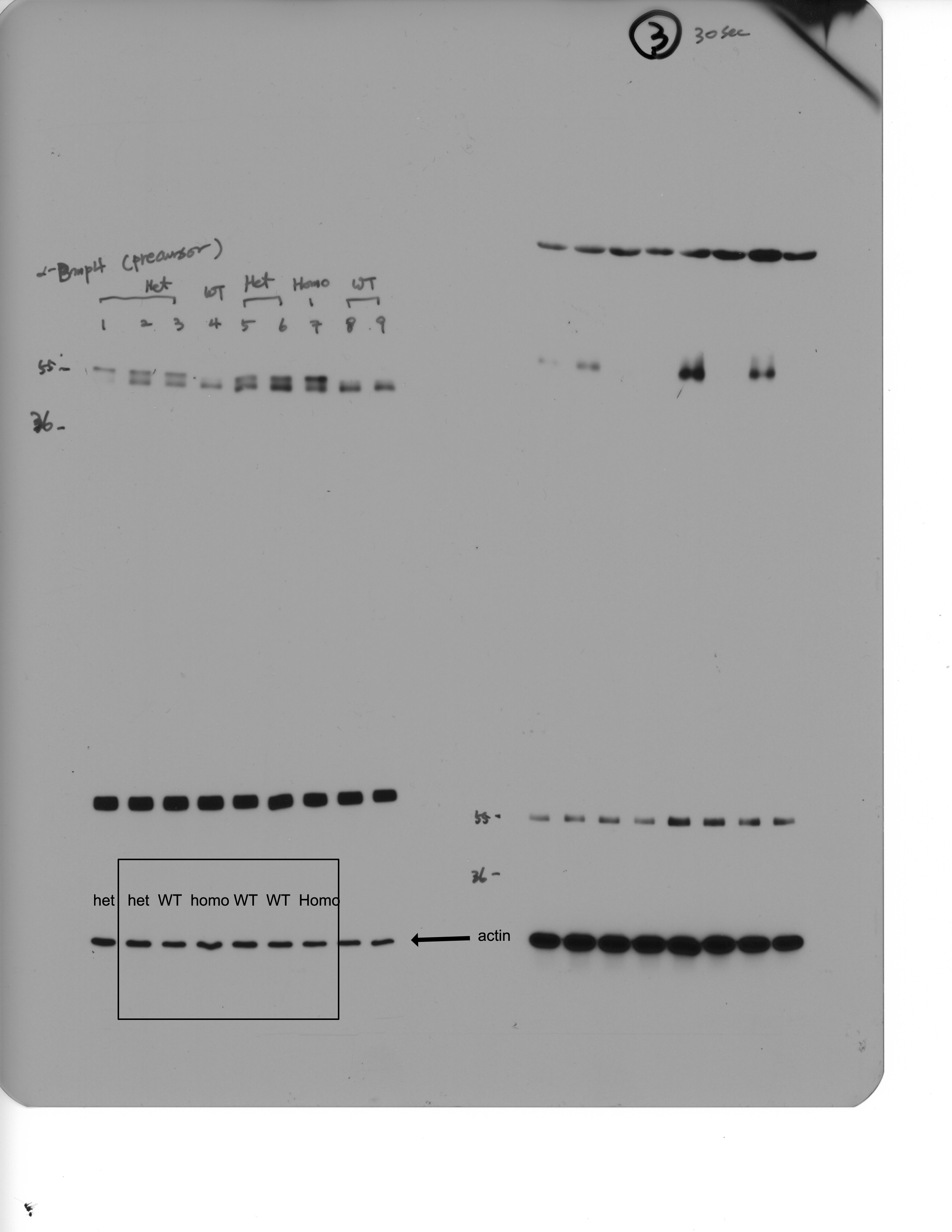

Supplement: Figure 6—source data 1. [file elife-105018-fig6-data1.zip › Figure 4-source data 1/J018_003 actin marked.tif]

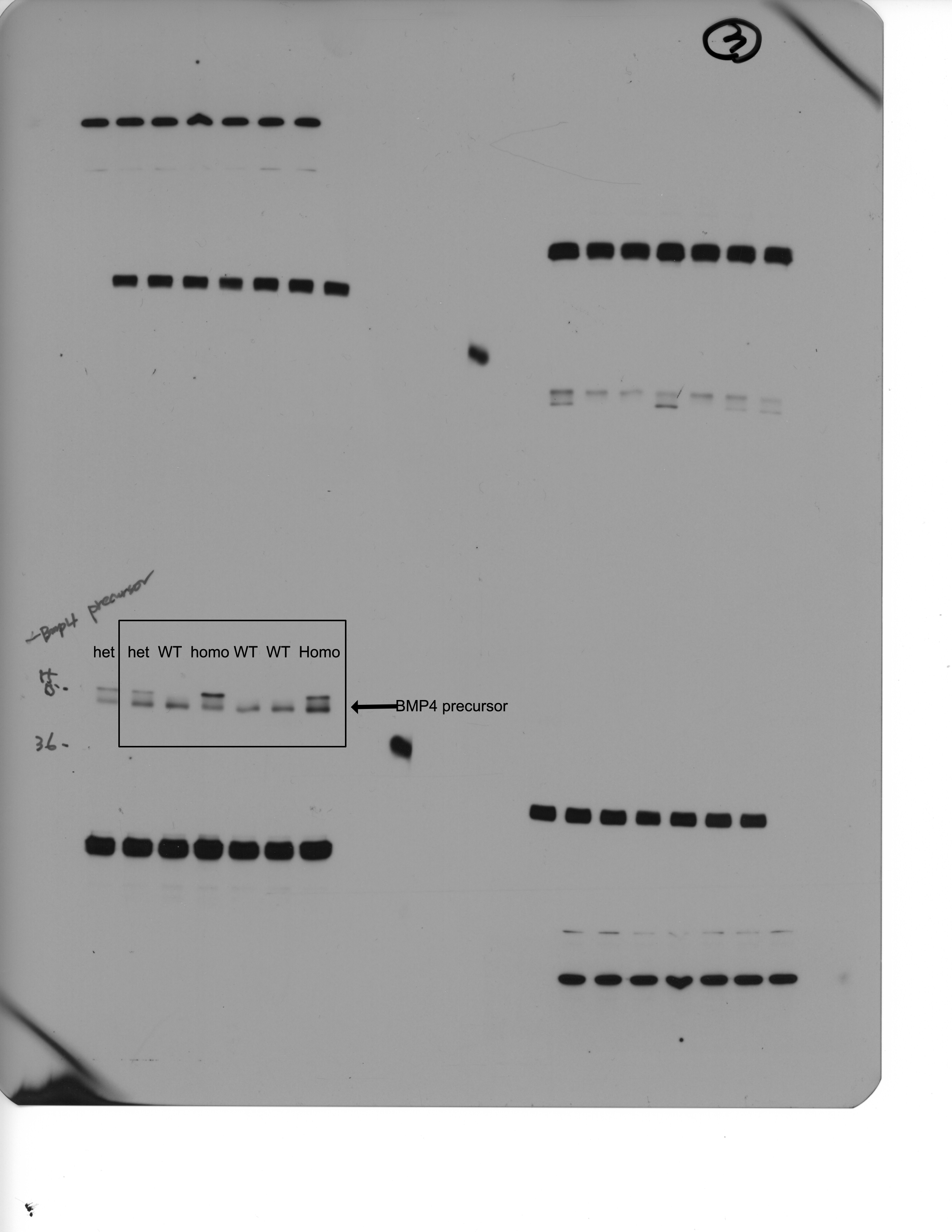

Supplement: Figure 6—source data 1. [file elife-105018-fig6-data1.zip › Figure 4-source data 1/J017_003 BMP4 precursor marked.tif]

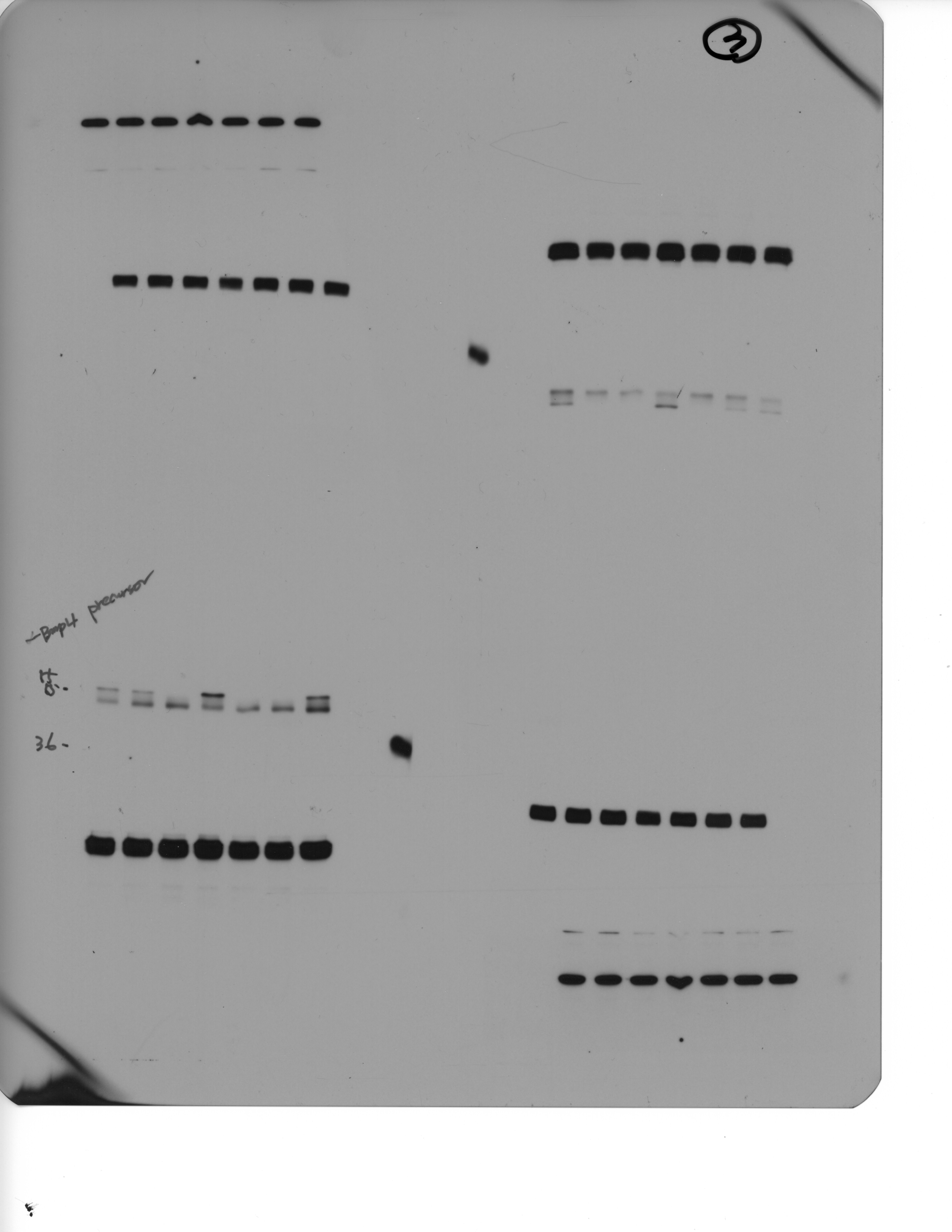

Supplement: Figure 6—source data 2. [file elife-105018-fig6-data2.zip › Figure 4-source data 2/J017_003 BMP4 precursor unmarked.tif]

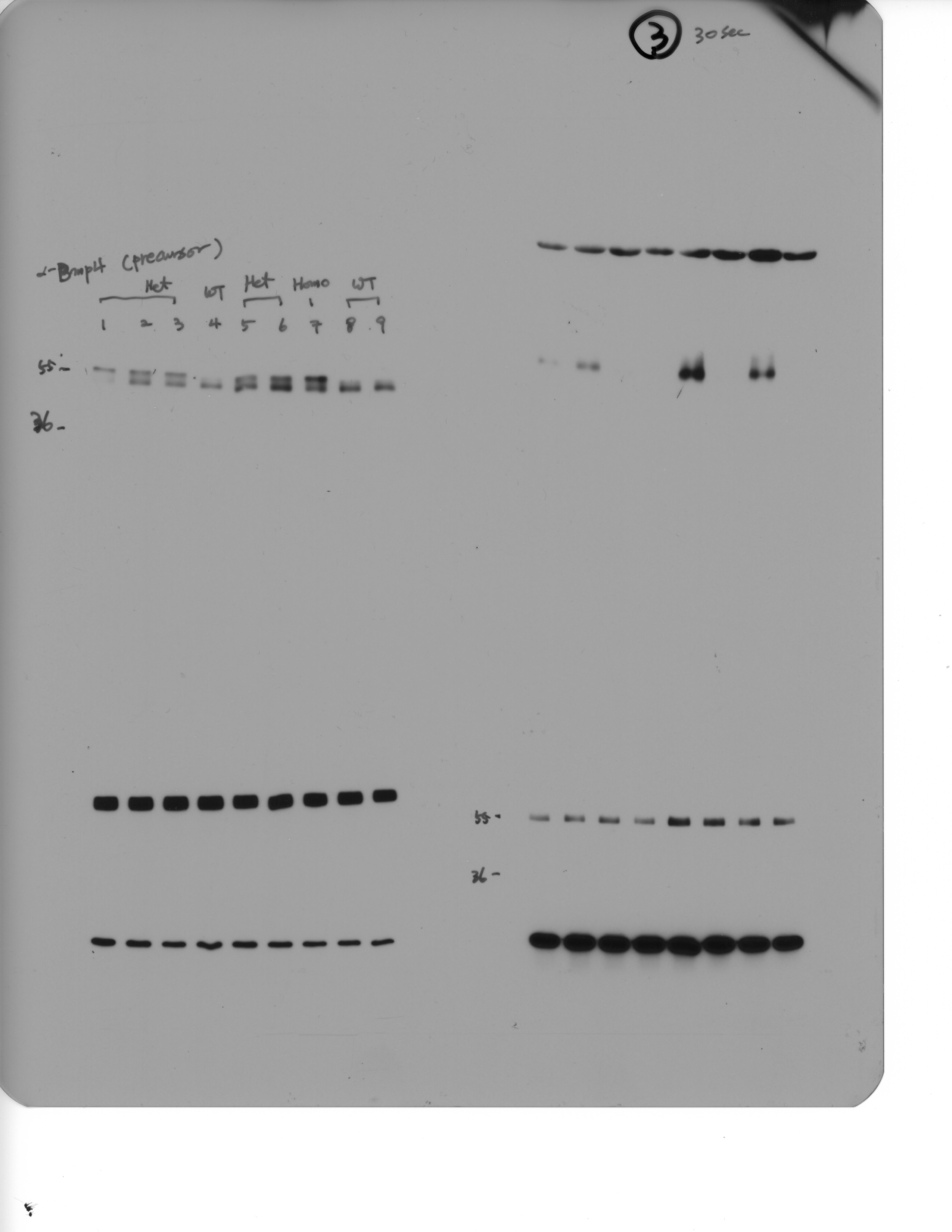

Supplement: Figure 6—source data 2. [file elife-105018-fig6-data2.zip › Figure 4-source data 2/J018_003 actin unmarked.tif]

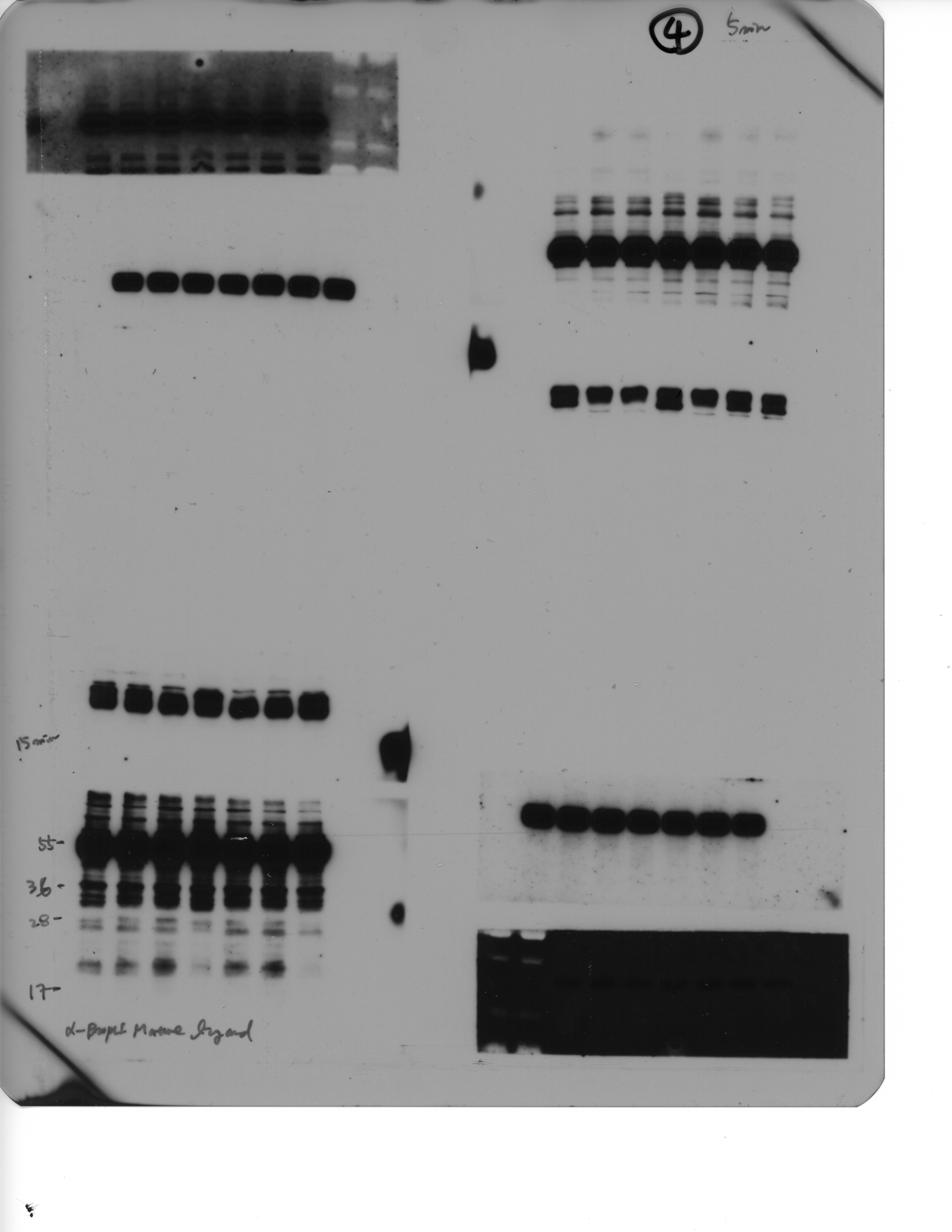

Supplement: Figure 6—source data 2. [file elife-105018-fig6-data2.zip › Figure 4-source data 2/J017_004 BMP4 ligand unmarked.tif]

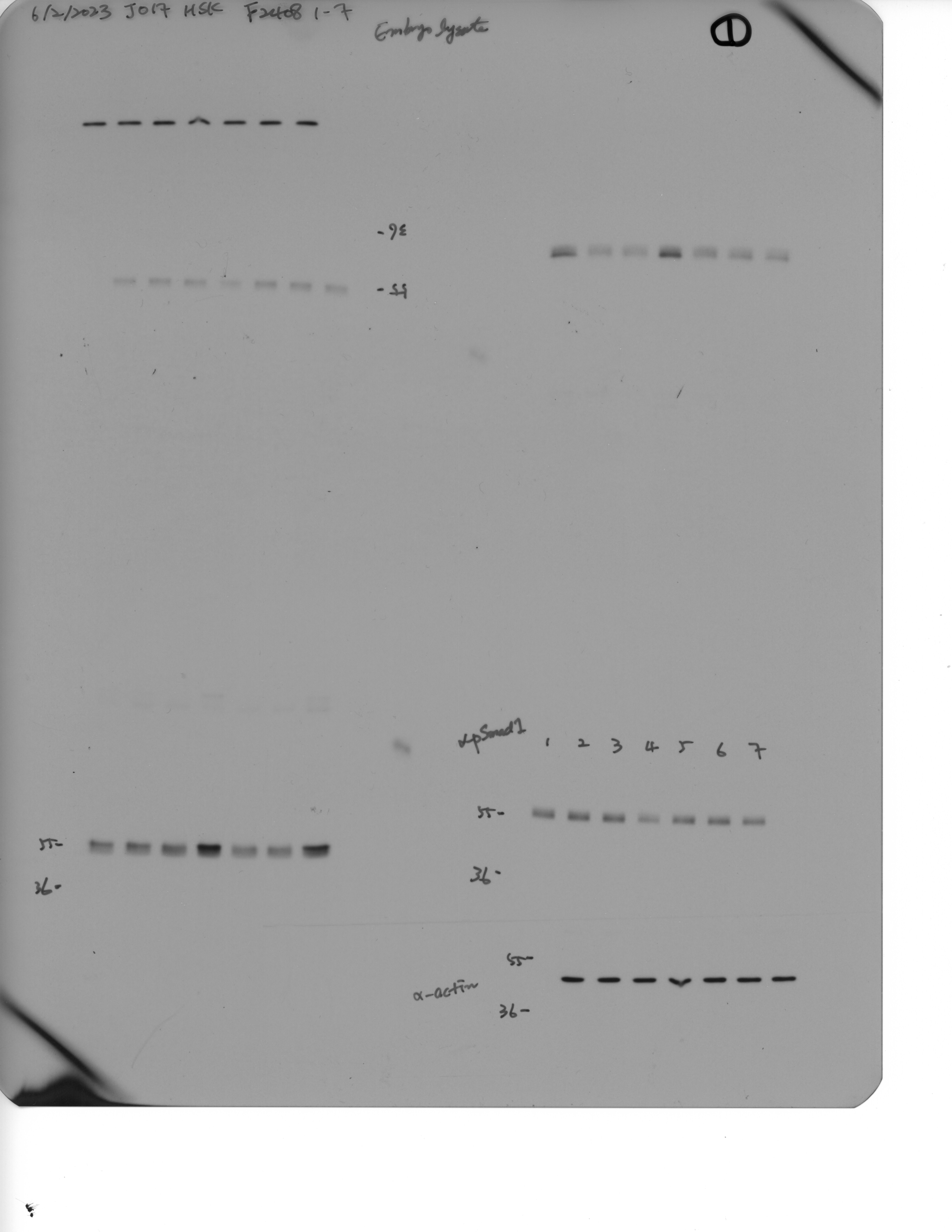

Supplement: Figure 6—source data 2. [file elife-105018-fig6-data2.zip › Figure 4-source data 2/J017_001 pSmad1 unmarked.tif]

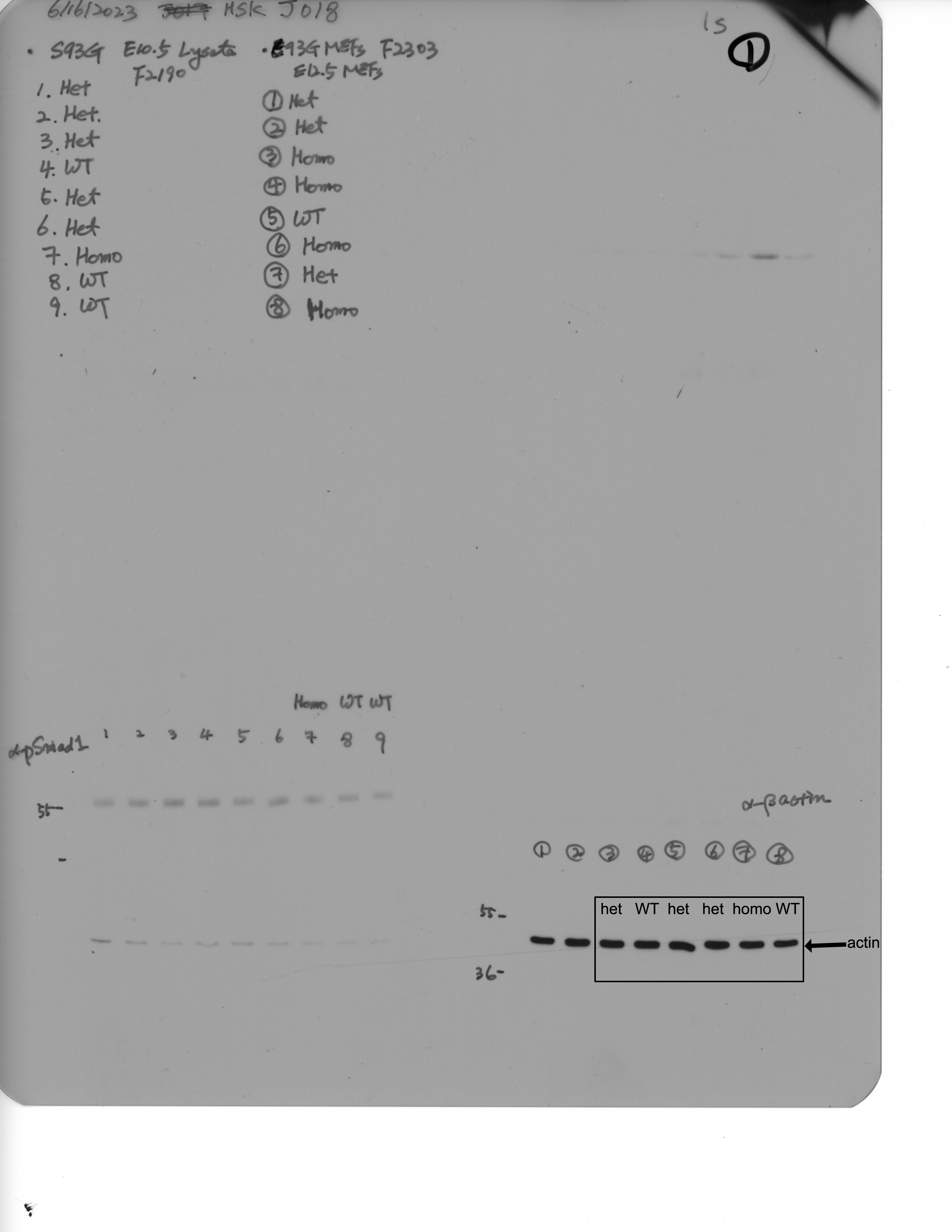

Supplement: Figure 6—source data 3. [file elife-105018-fig6-data3.zip › Figure 4-source data 3/E93G E10.5 actin marked .tif]

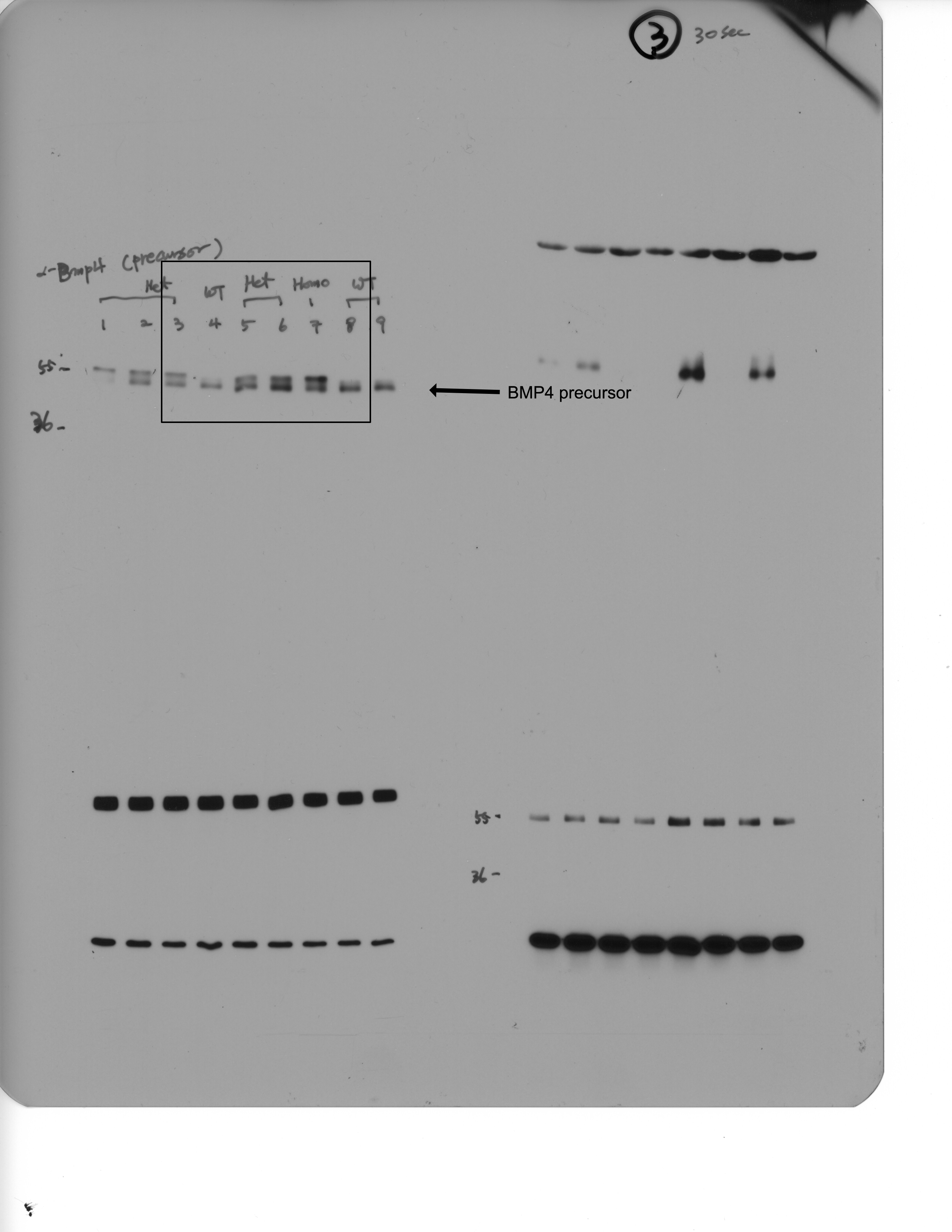

Supplement: Figure 6—source data 3. [file elife-105018-fig6-data3.zip › Figure 4-source data 3/J018_003 precursor marked .tif]

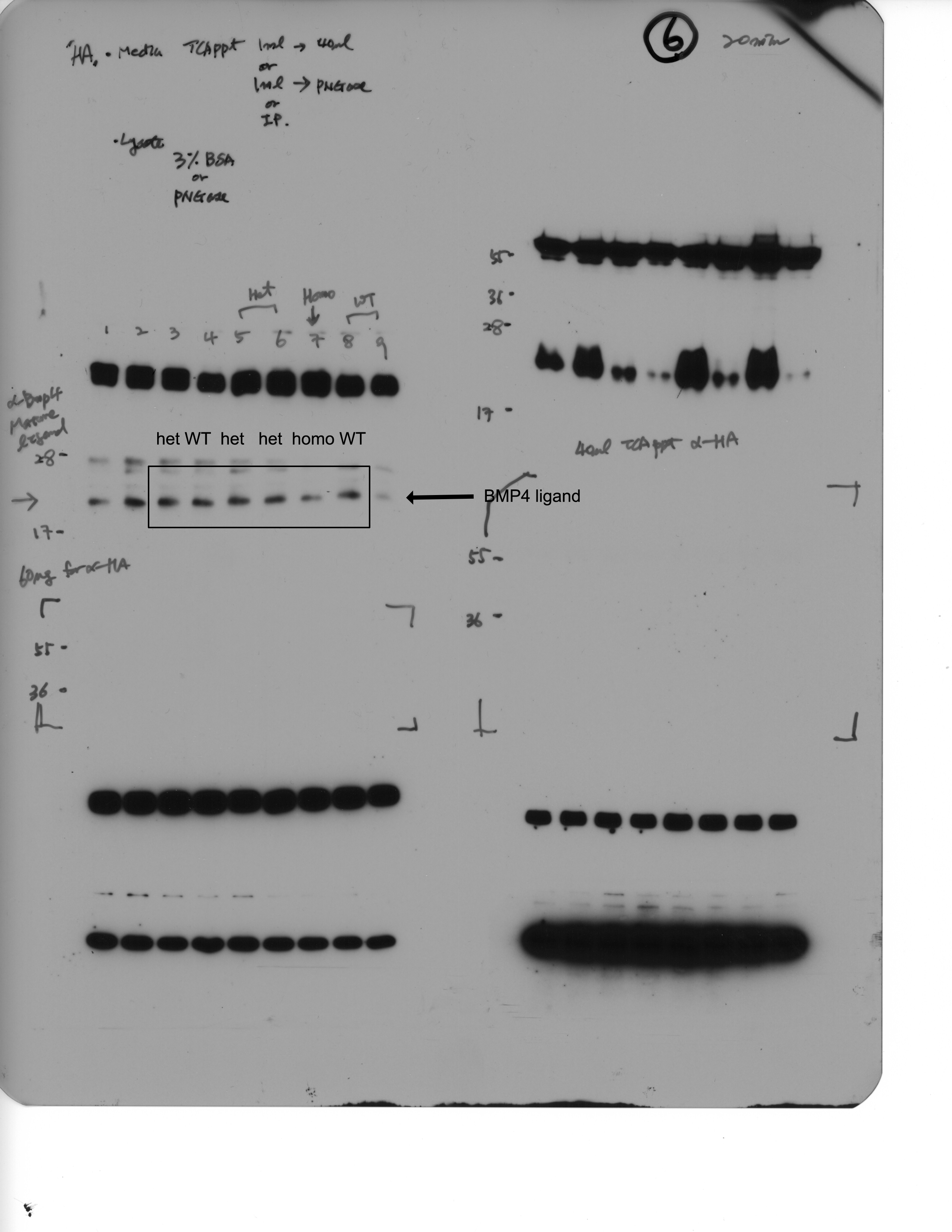

Supplement: Figure 6—source data 3. [file elife-105018-fig6-data3.zip › Figure 4-source data 3/J018_006 ligand marked.tif]

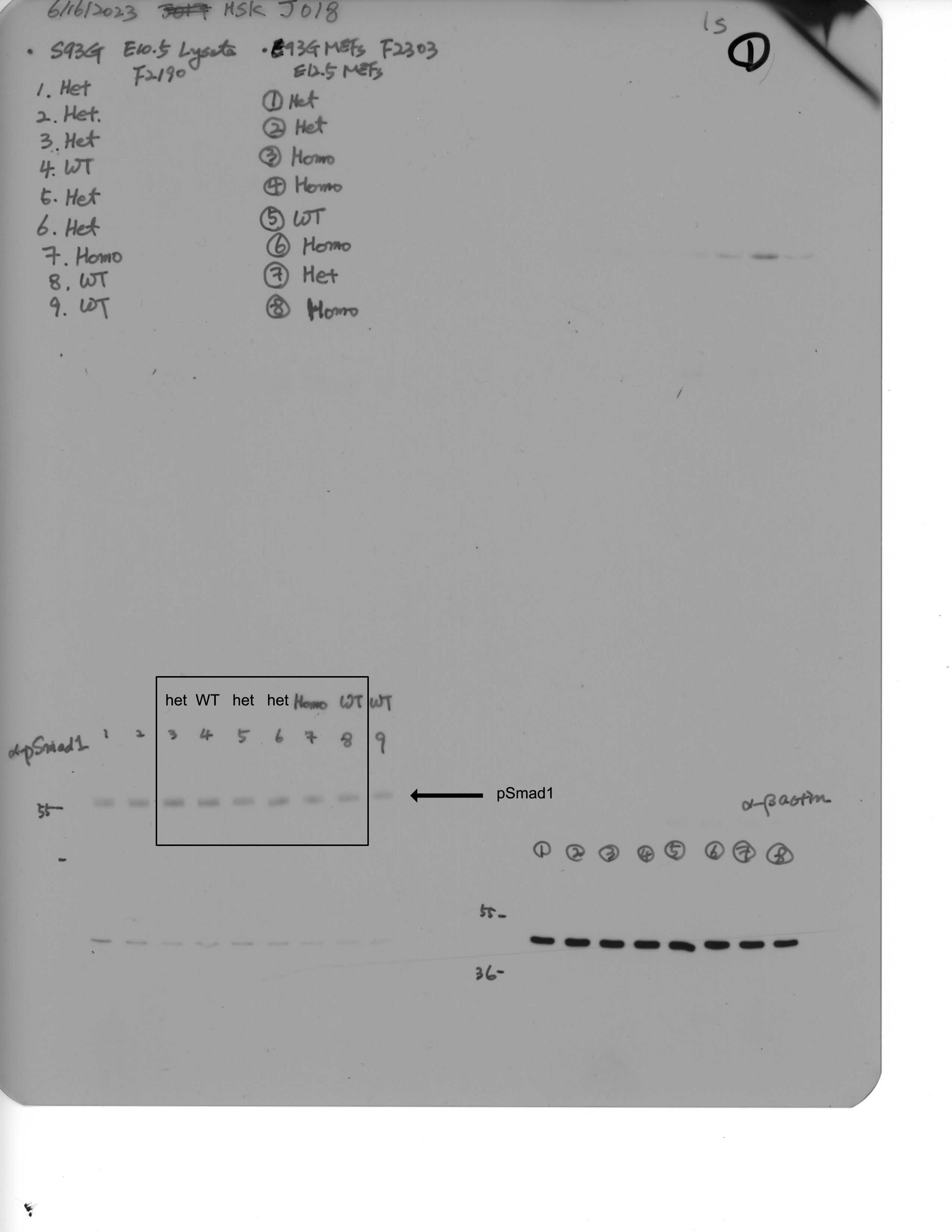

Supplement: Figure 6—source data 3. [file elife-105018-fig6-data3.zip › Figure 4-source data 3/E93G E10.5 pSmad1 marked (J018_1).tif]

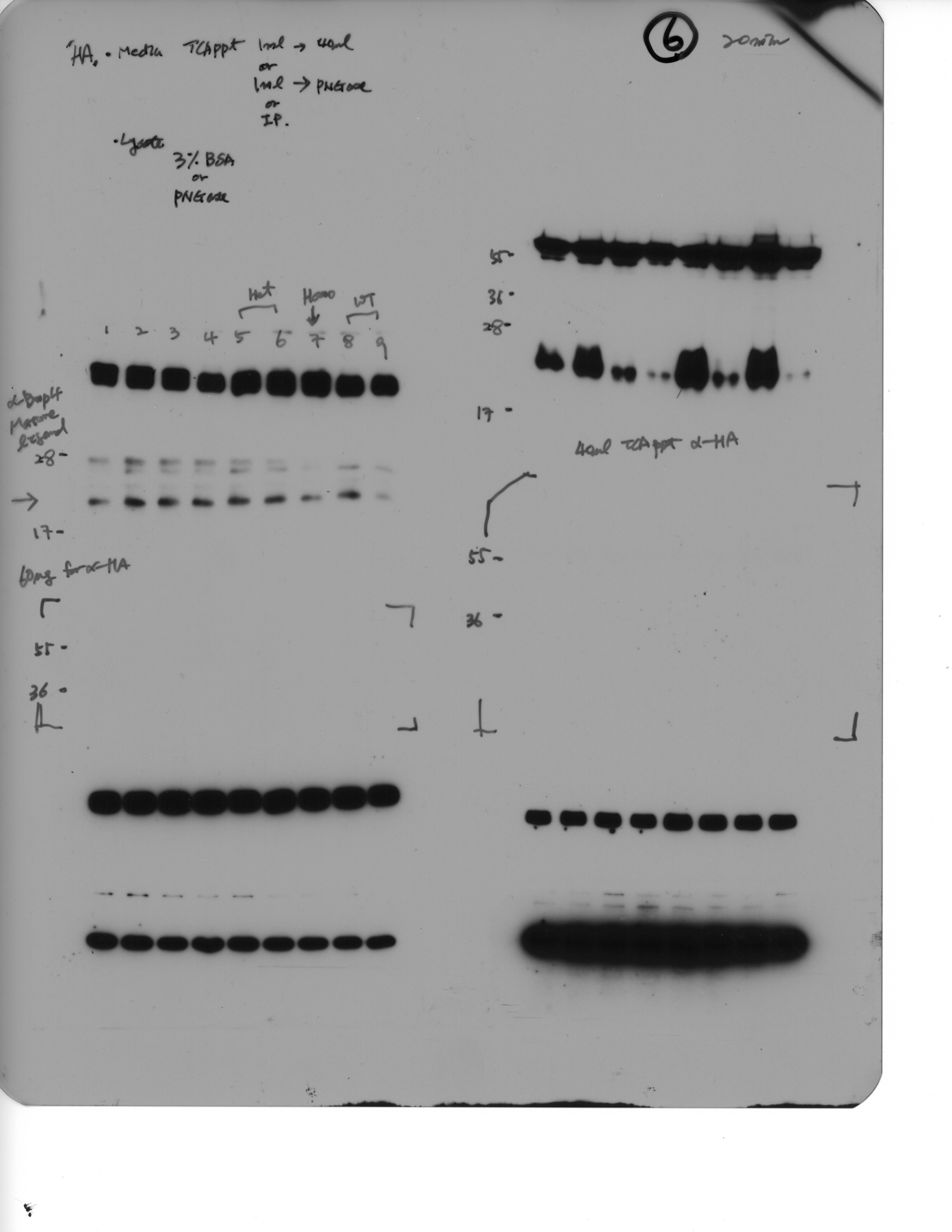

Supplement: Figure 6—source data 4. [file elife-105018-fig6-data4.zip › Figure 4-source data 4/J018_006 ligand unmarked.tif]

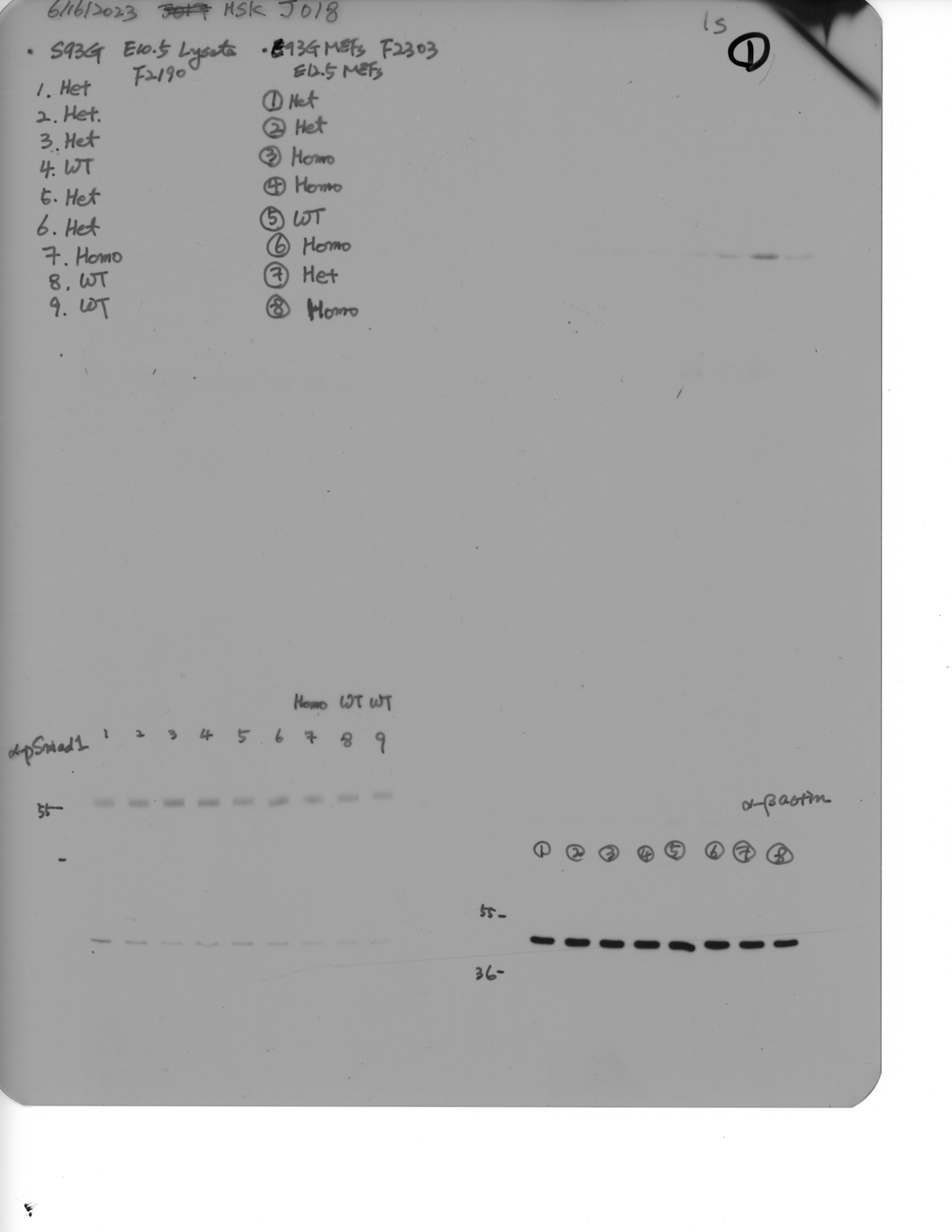

Supplement: Figure 6—source data 4. [file elife-105018-fig6-data4.zip › Figure 4-source data 4/E93G E10.5 pSmad1 unmarked (J018_1).tif]

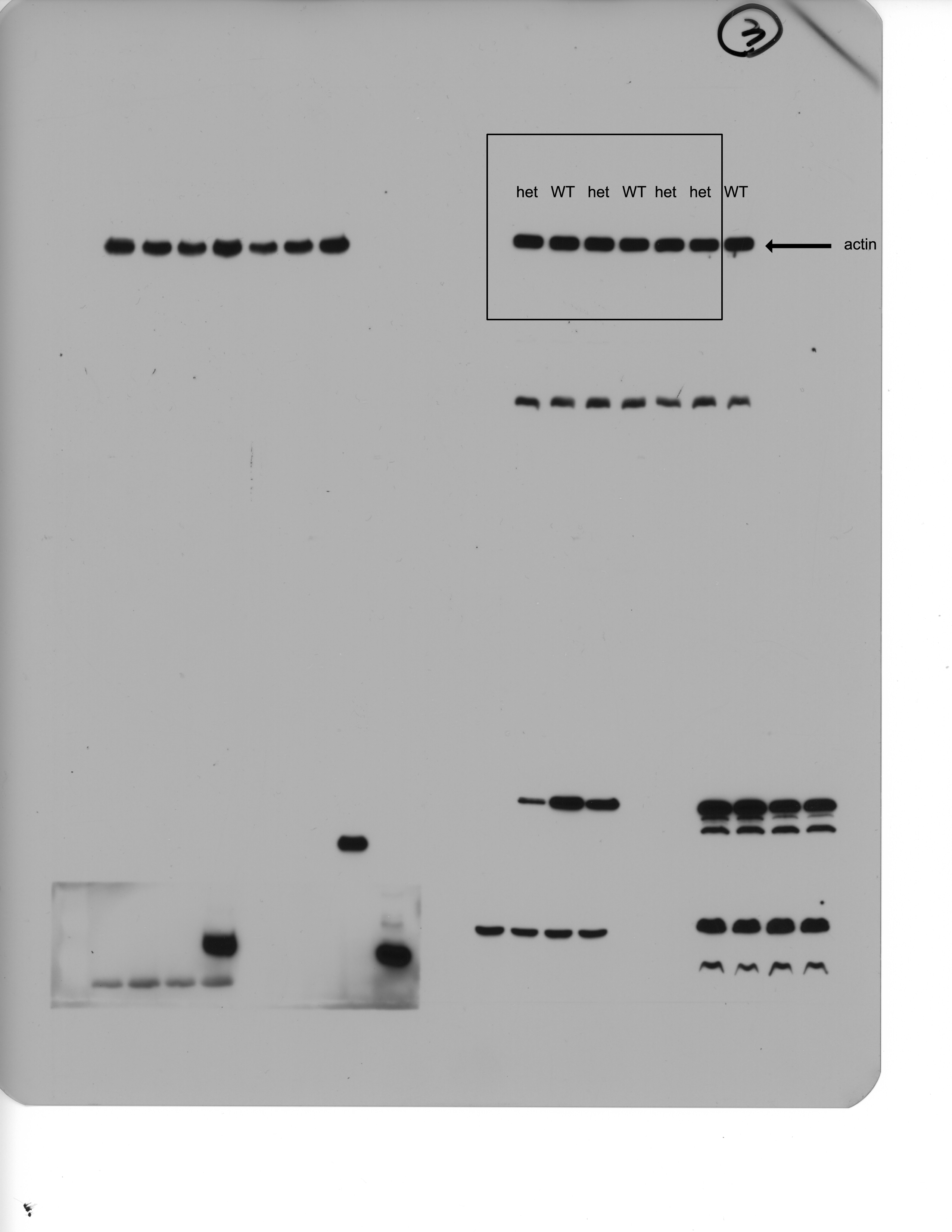

Supplement: Figure 6—source data 5. [file elife-105018-fig6-data5.zip › Figure 4-source data 5/4I actin_marked.tif]

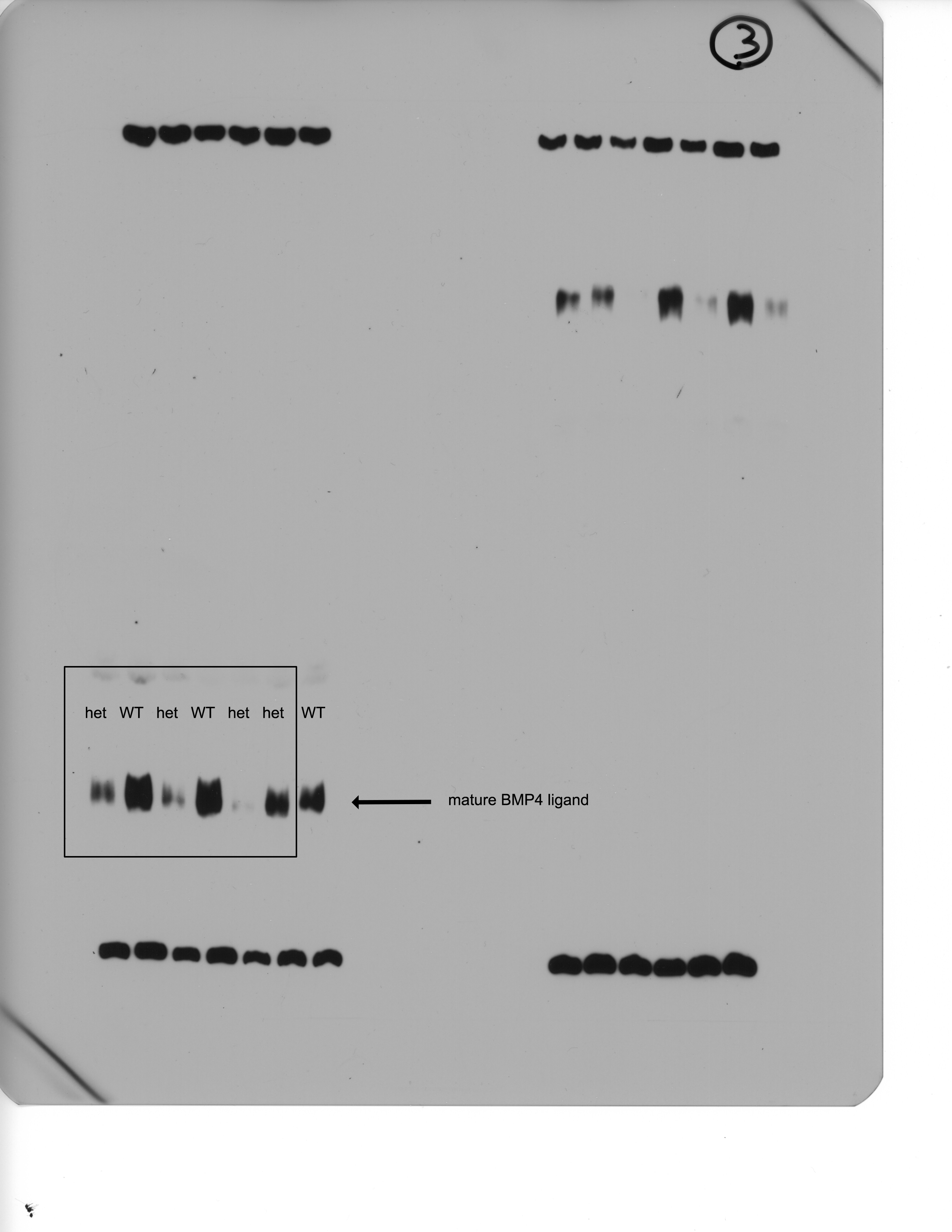

Supplement: Figure 6—source data 5. [file elife-105018-fig6-data5.zip › Figure 4-source data 5/Fig. 4I ligand_marked.tif]

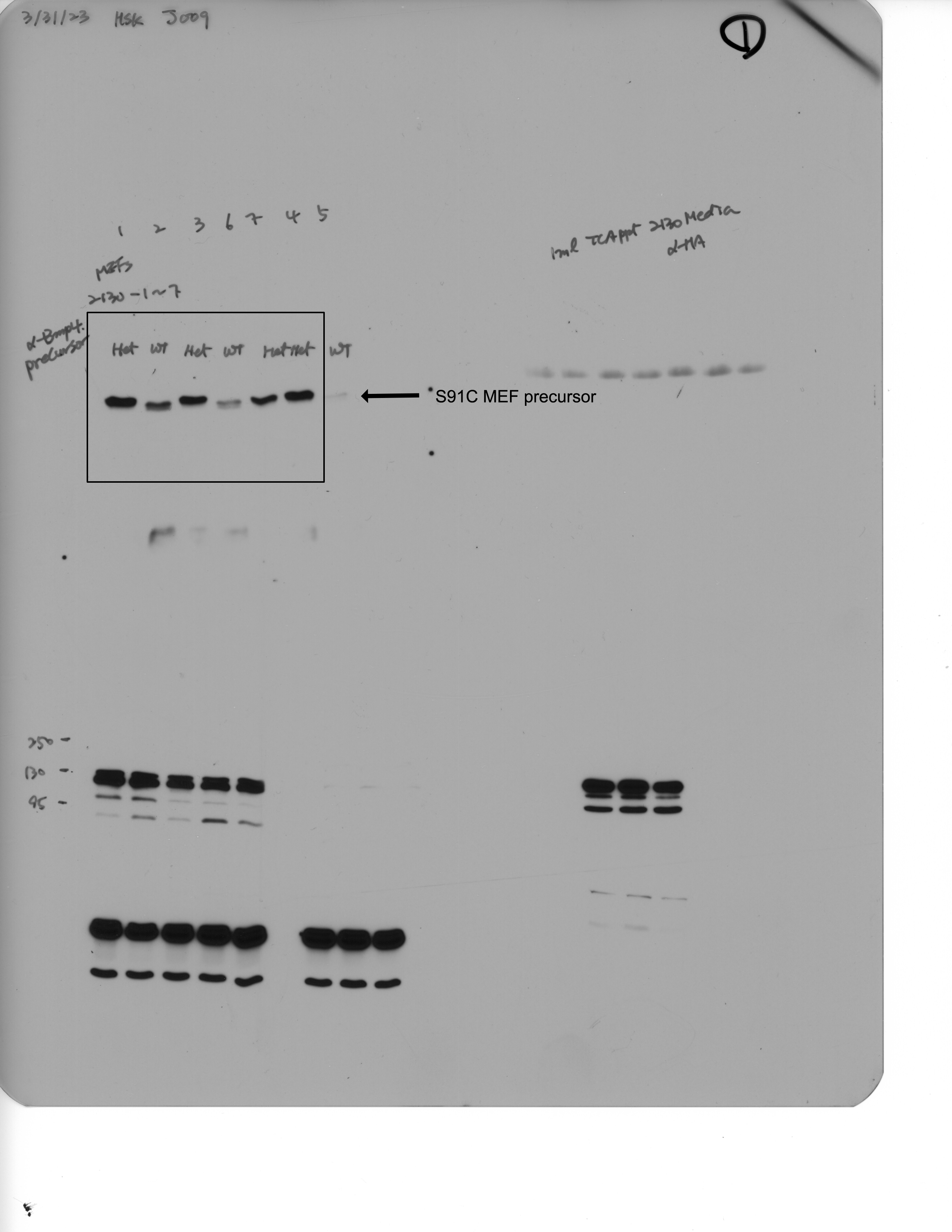

Supplement: Figure 6—source data 5. [file elife-105018-fig6-data5.zip › Figure 4-source data 5/Fig.4I precursor_marked.tif]

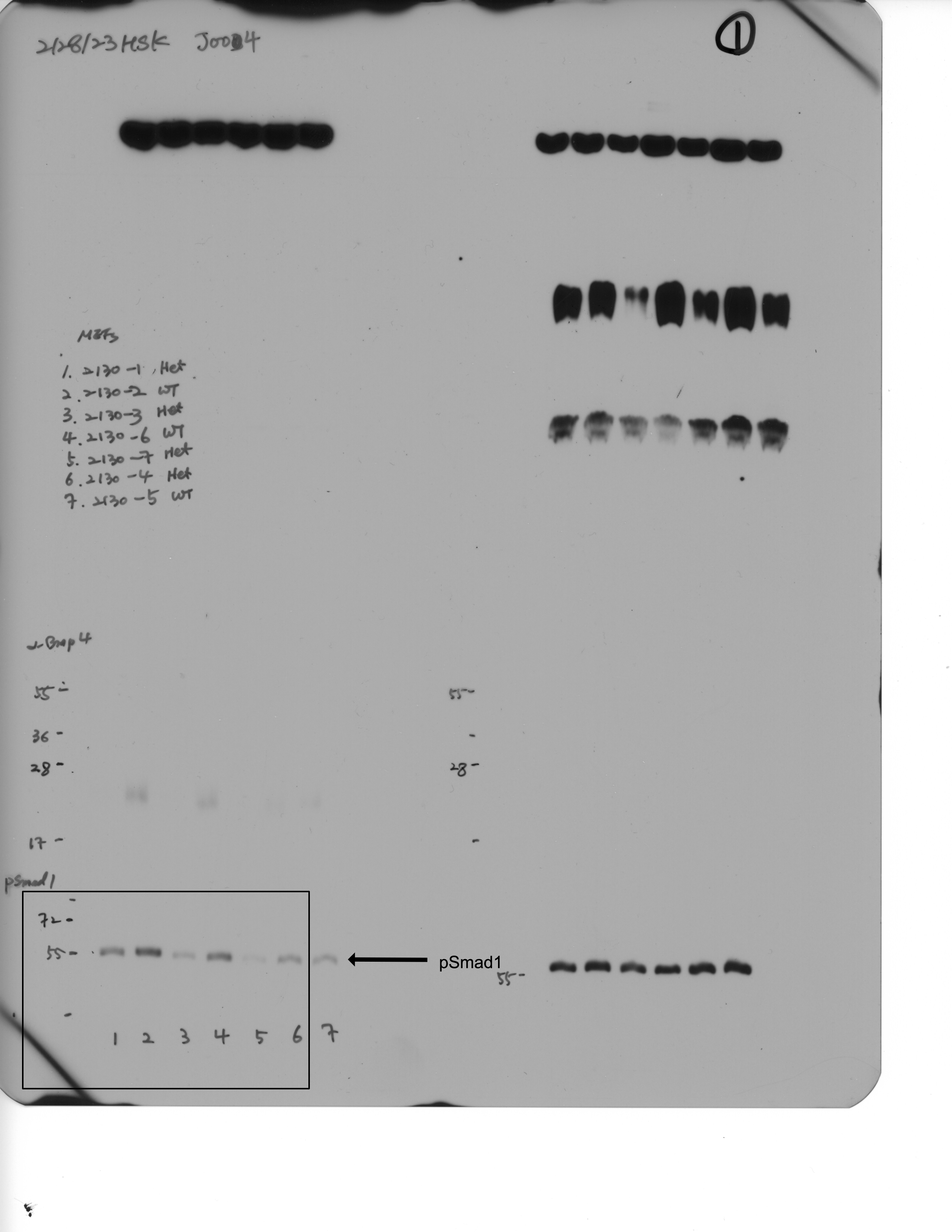

Supplement: Figure 6—source data 5. [file elife-105018-fig6-data5.zip › Figure 4-source data 5/Fig. 4I pSmad1_marked.tif]

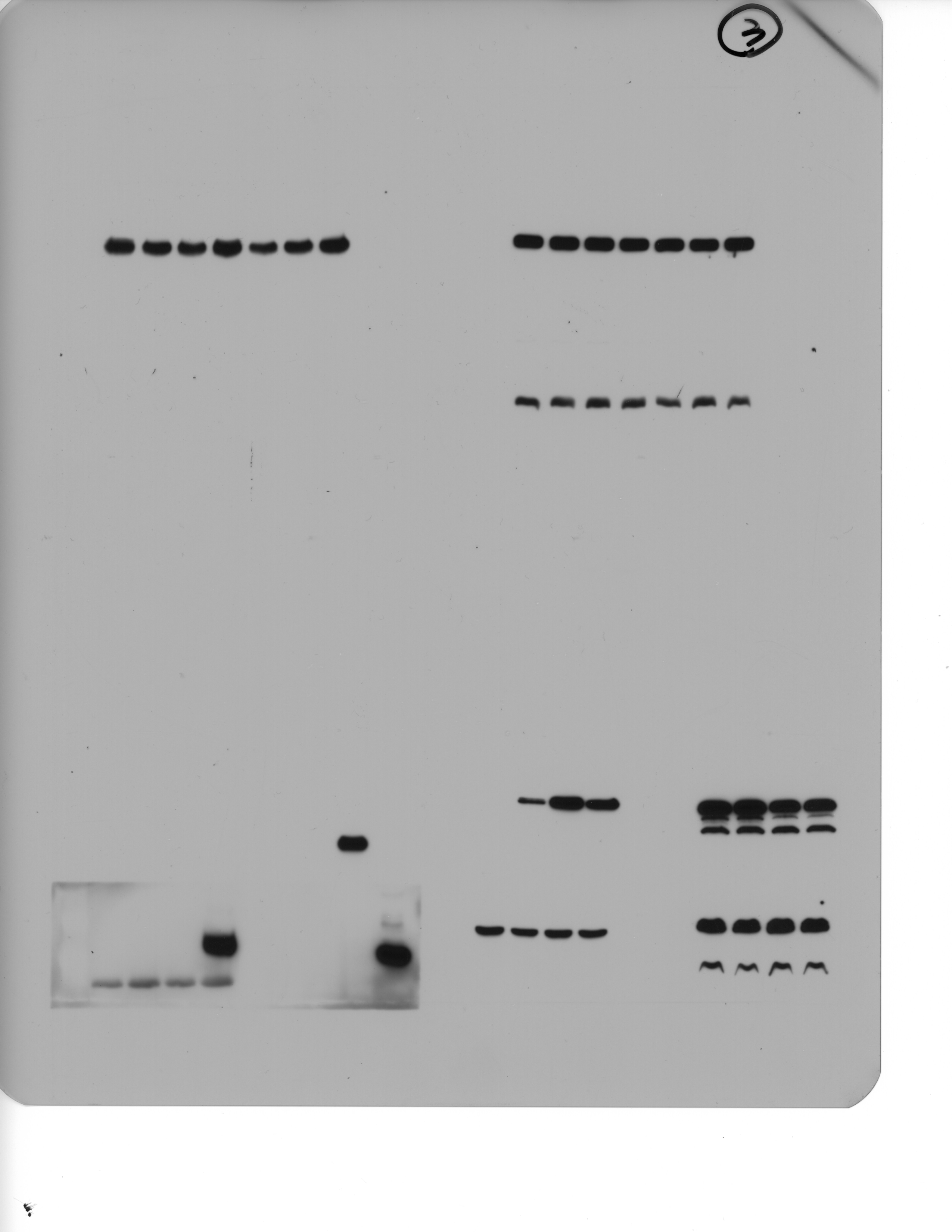

Supplement: Figure 6—source data 6. [file elife-105018-fig6-data6.zip › Figure 4-source data 6/4I actin_unmarked.tif]

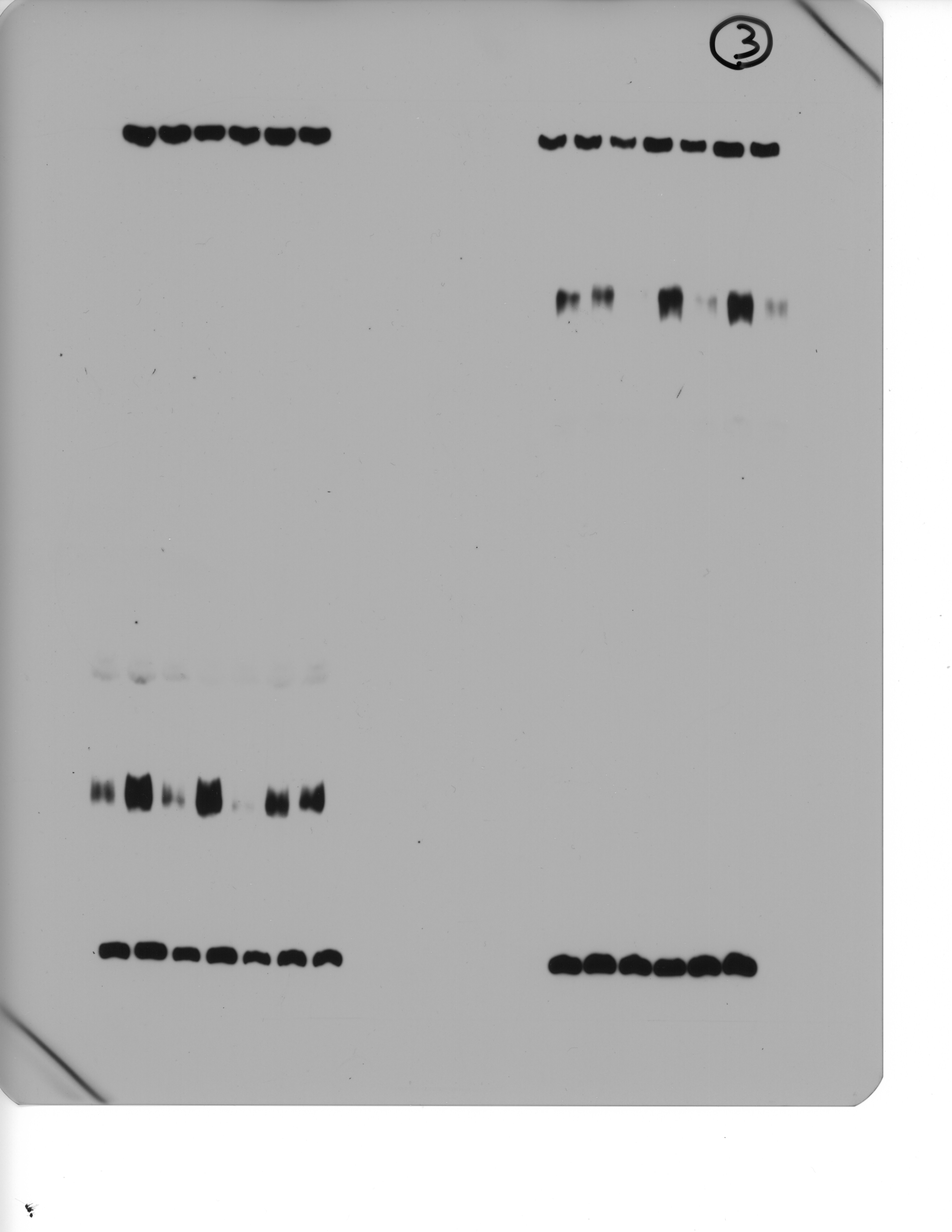

Supplement: Figure 6—source data 6. [file elife-105018-fig6-data6.zip › Figure 4-source data 6/Fig. 4I ligand_unmarked.tif]

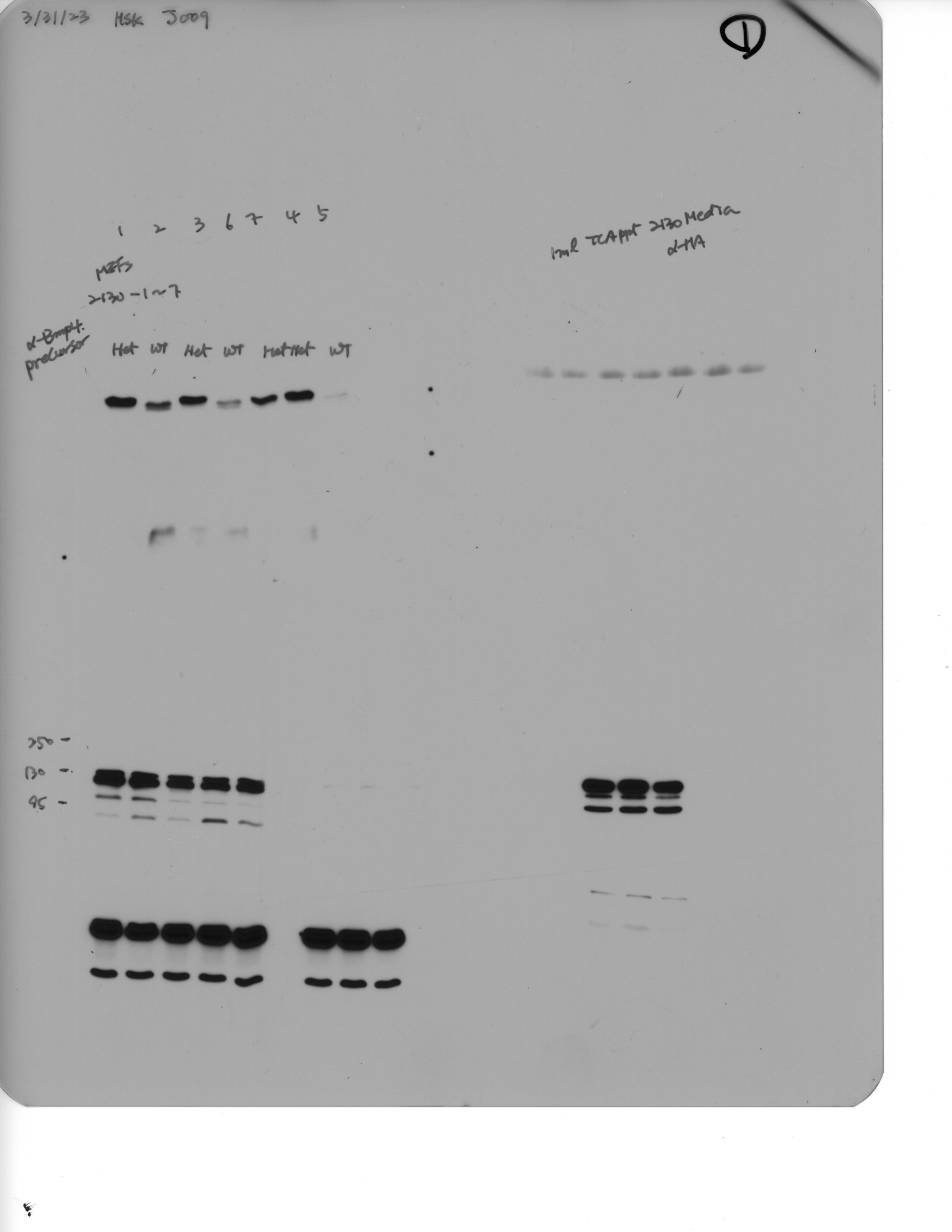

Supplement: Figure 6—source data 6. [file elife-105018-fig6-data6.zip › Figure 4-source data 6/Fig.4I precursor, unmarked.png]

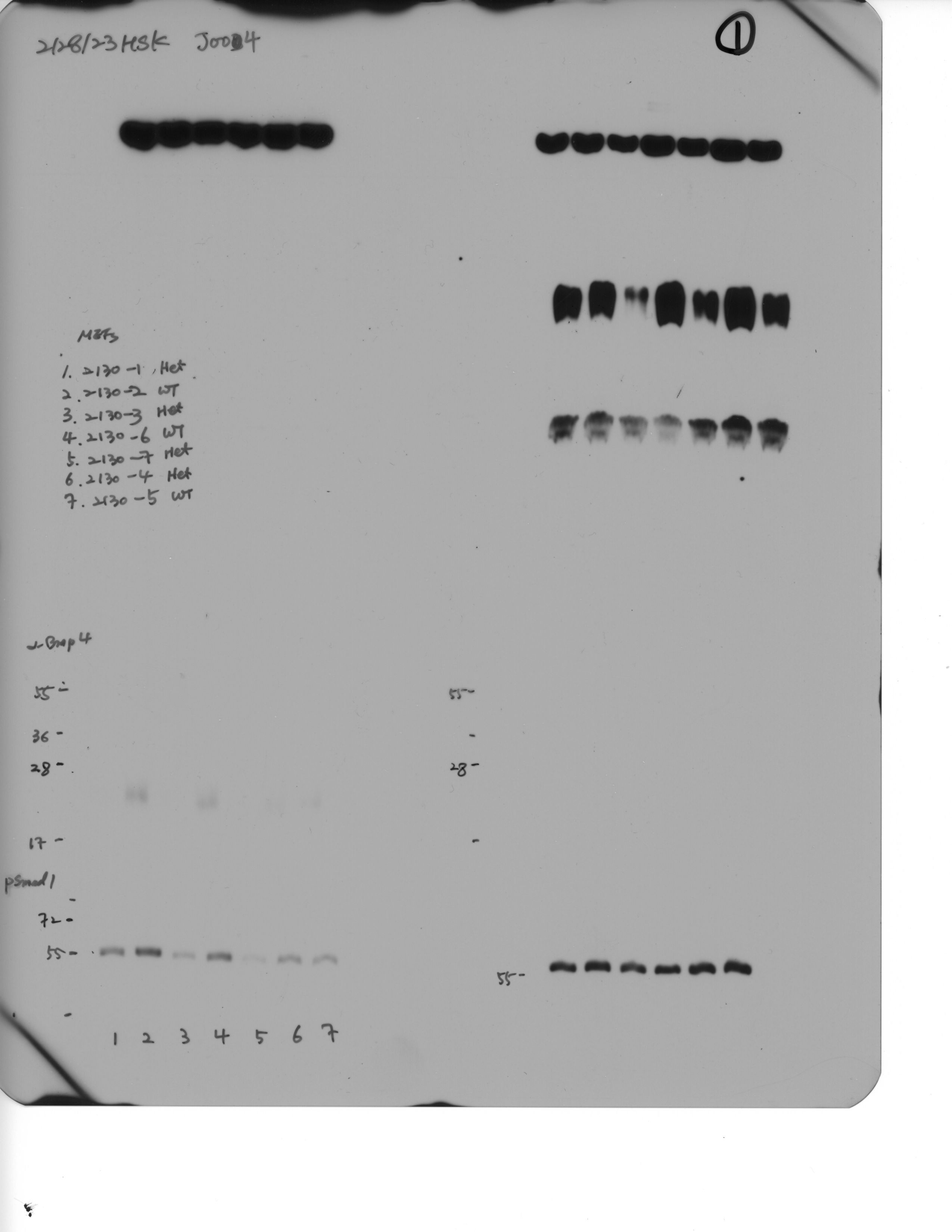

Supplement: Figure 6—source data 6. [file elife-105018-fig6-data6.zip › Figure 4-source data 6/Fig. 4I pSmad1unmarked.tif]

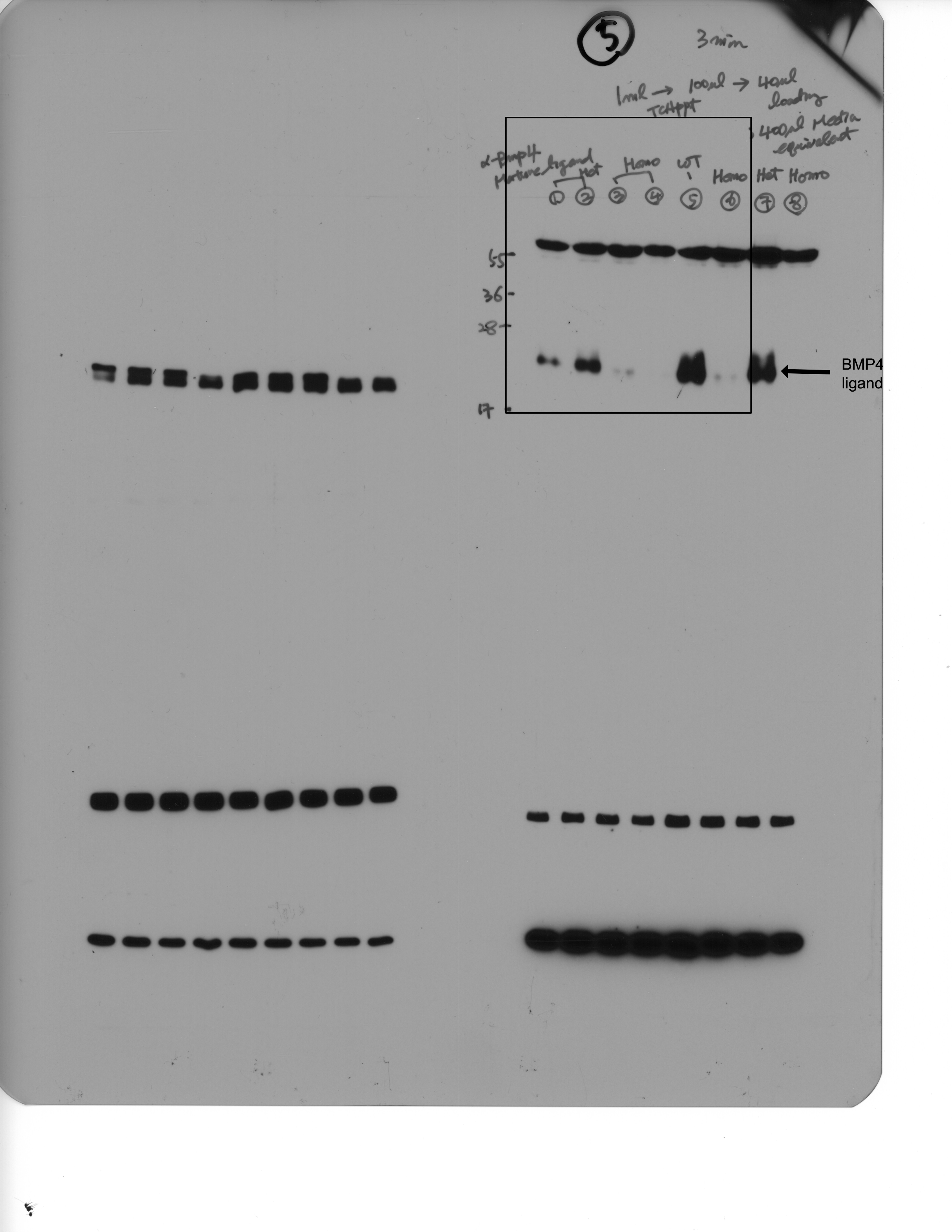

Supplement: Figure 6—source data 7. [file elife-105018-fig6-data7.zip › Figure4-source data 7/J018_005 ligand marked.tif]

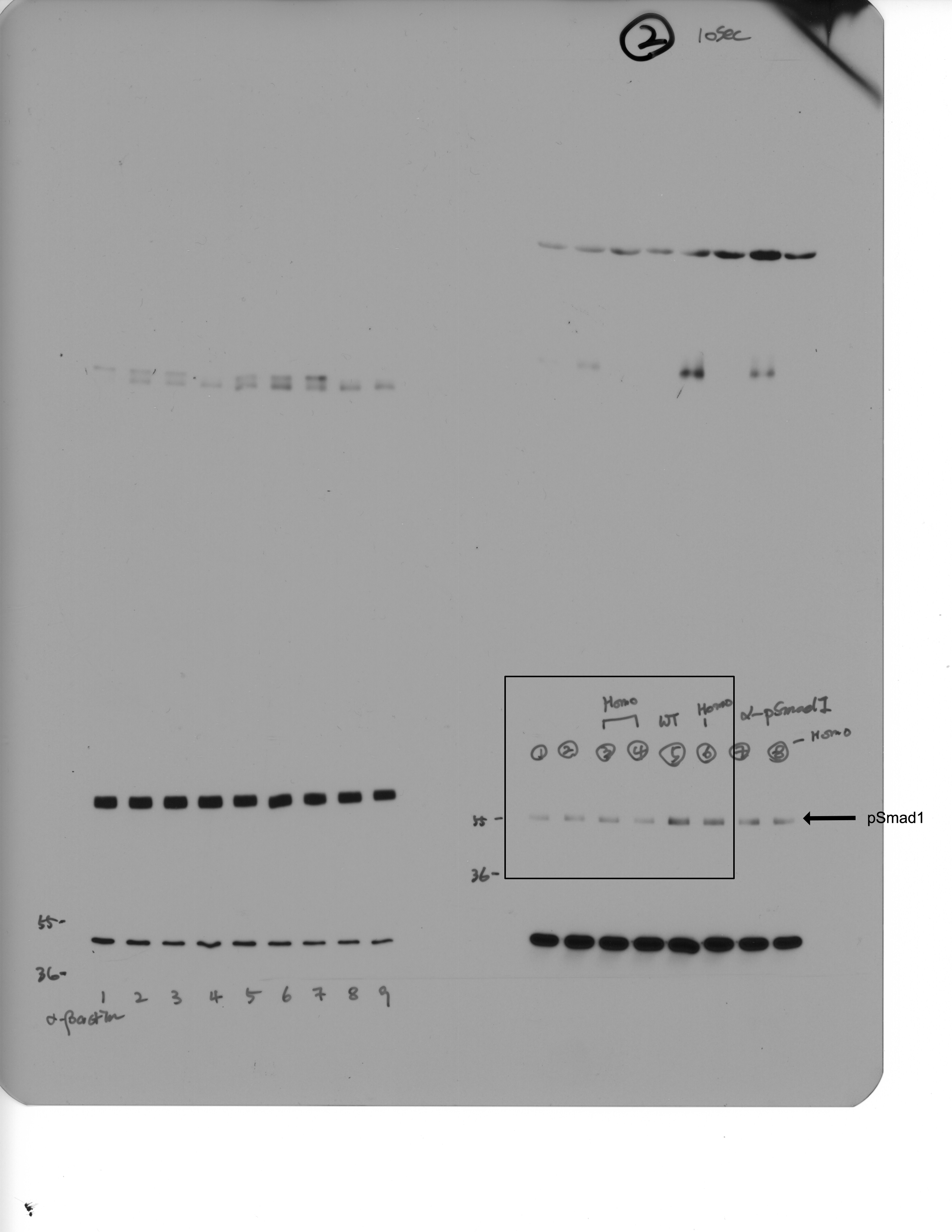

Supplement: Figure 6—source data 7. [file elife-105018-fig6-data7.zip › Figure4-source data 7/J018_4 E93G MEF pSmad1 marked.tif]

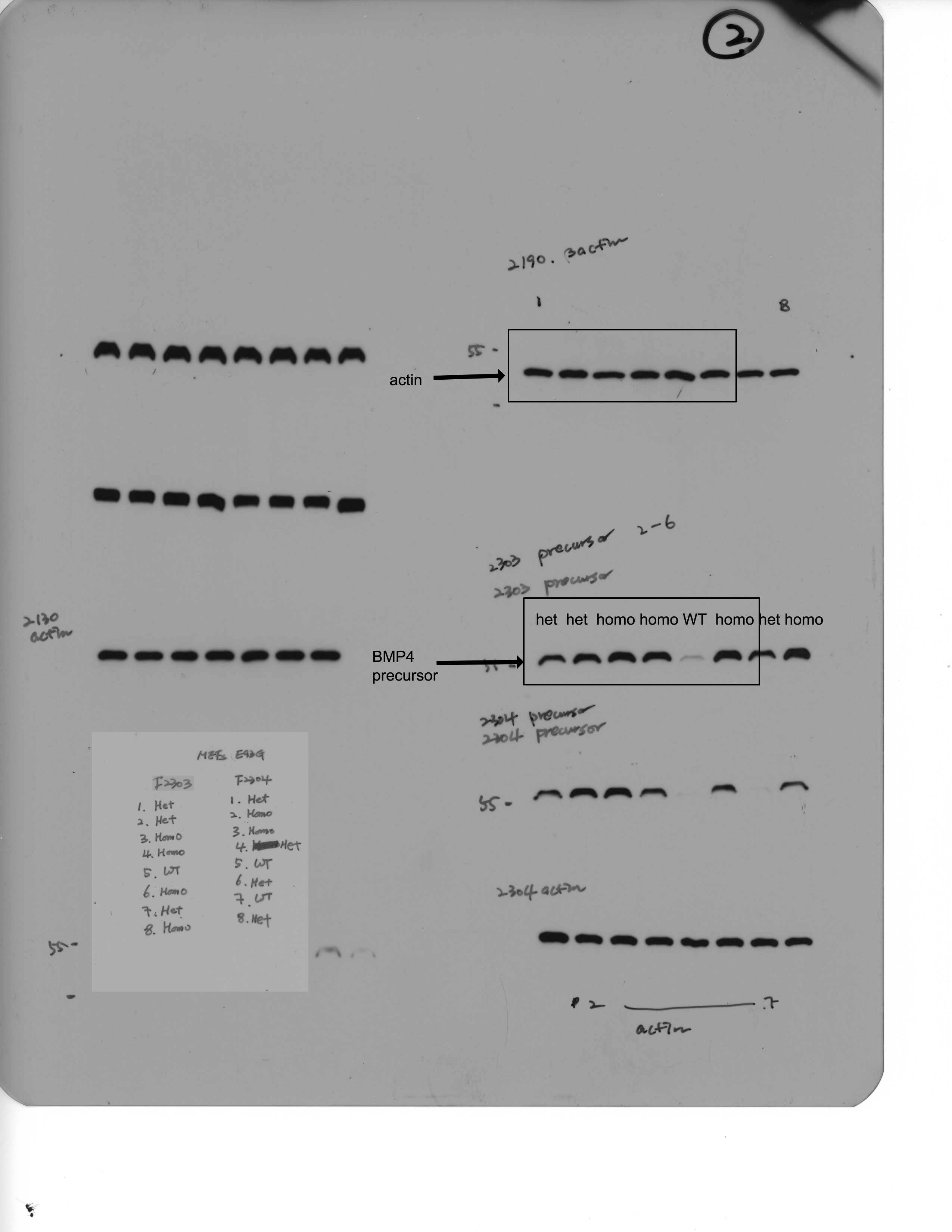

Supplement: Figure 6—source data 7. [file elife-105018-fig6-data7.zip › Figure4-source data 7/J021_002 precursor actin marked.tif]

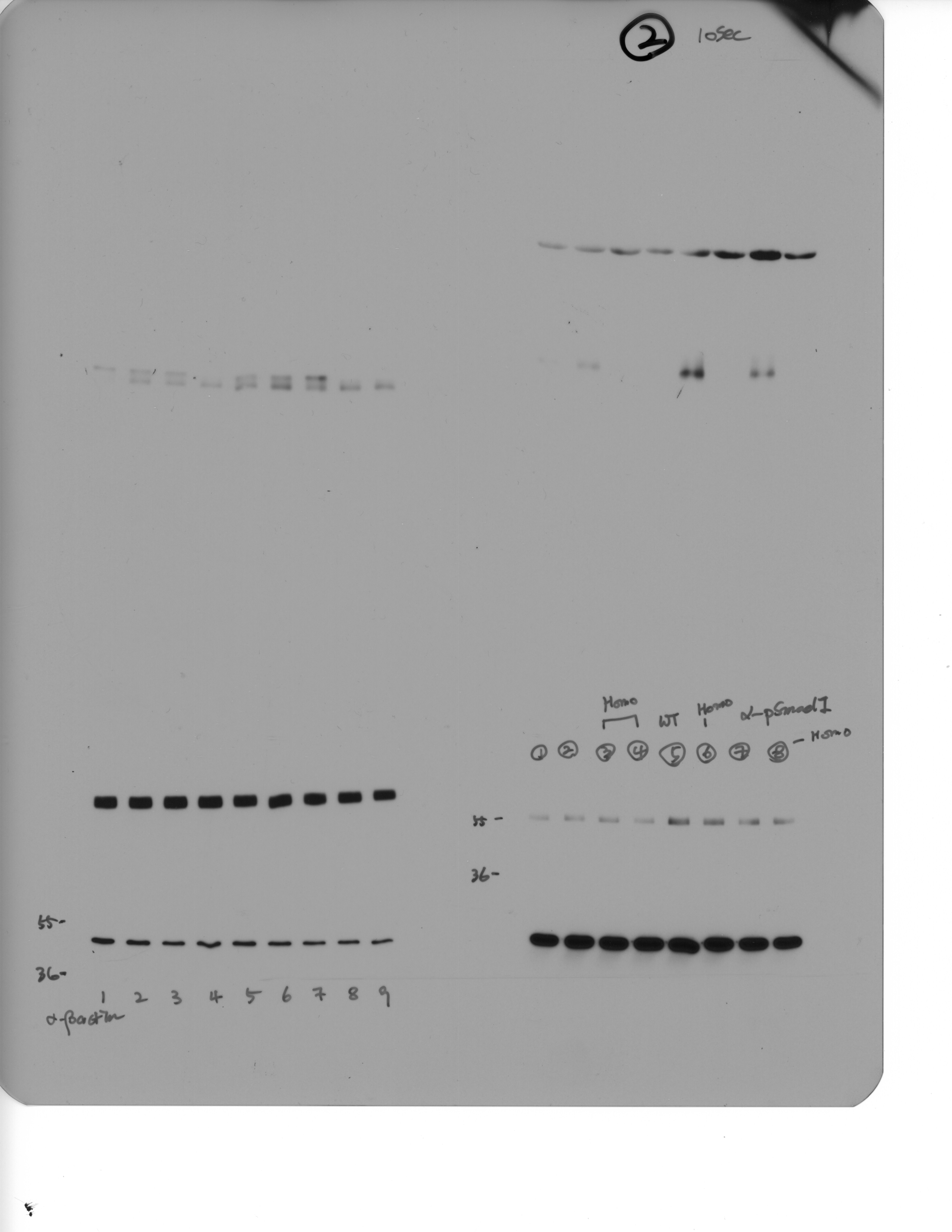

Supplement: Figure 6—source data 8. [file elife-105018-fig6-data8.zip › Figure 4-source data 8/J018_4 E93G MEF pSmad1 unmarked.tif]

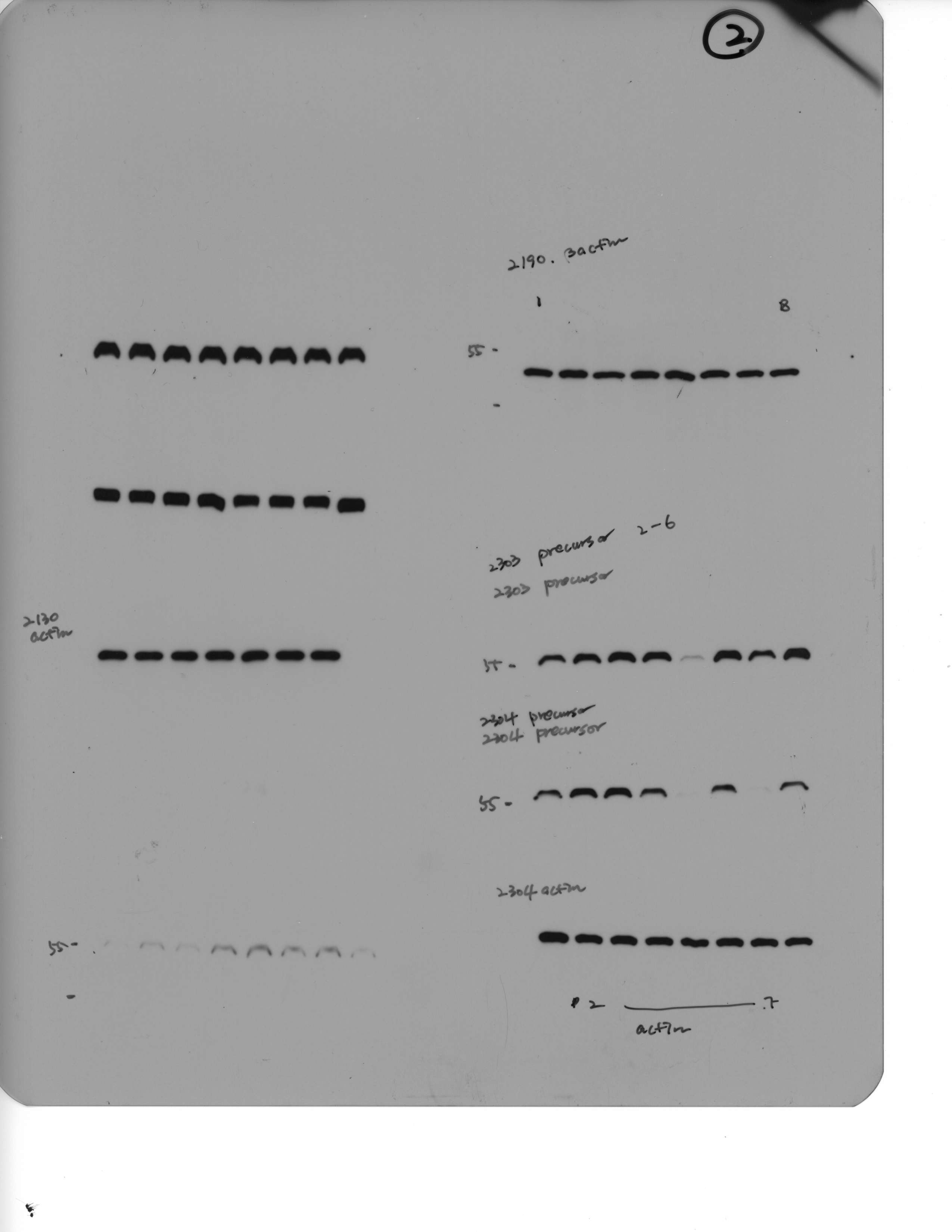

Supplement: Figure 6—source data 8. [file elife-105018-fig6-data8.zip › Figure 4-source data 8/J021_002 precursor actin unmarked.tif]

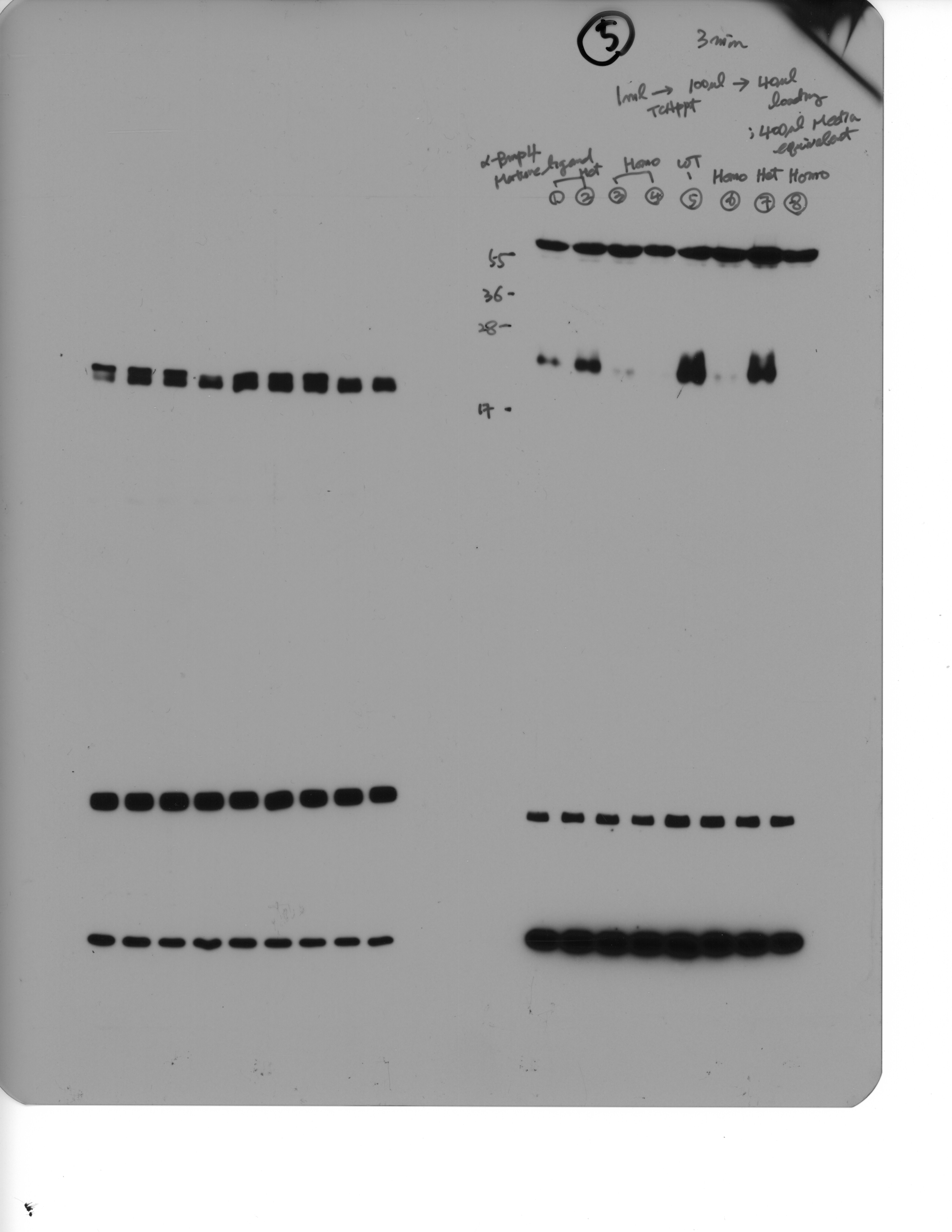

Supplement: Figure 6—source data 8. [file elife-105018-fig6-data8.zip › Figure 4-source data 8/J018_005 ligand unmarked.tif]

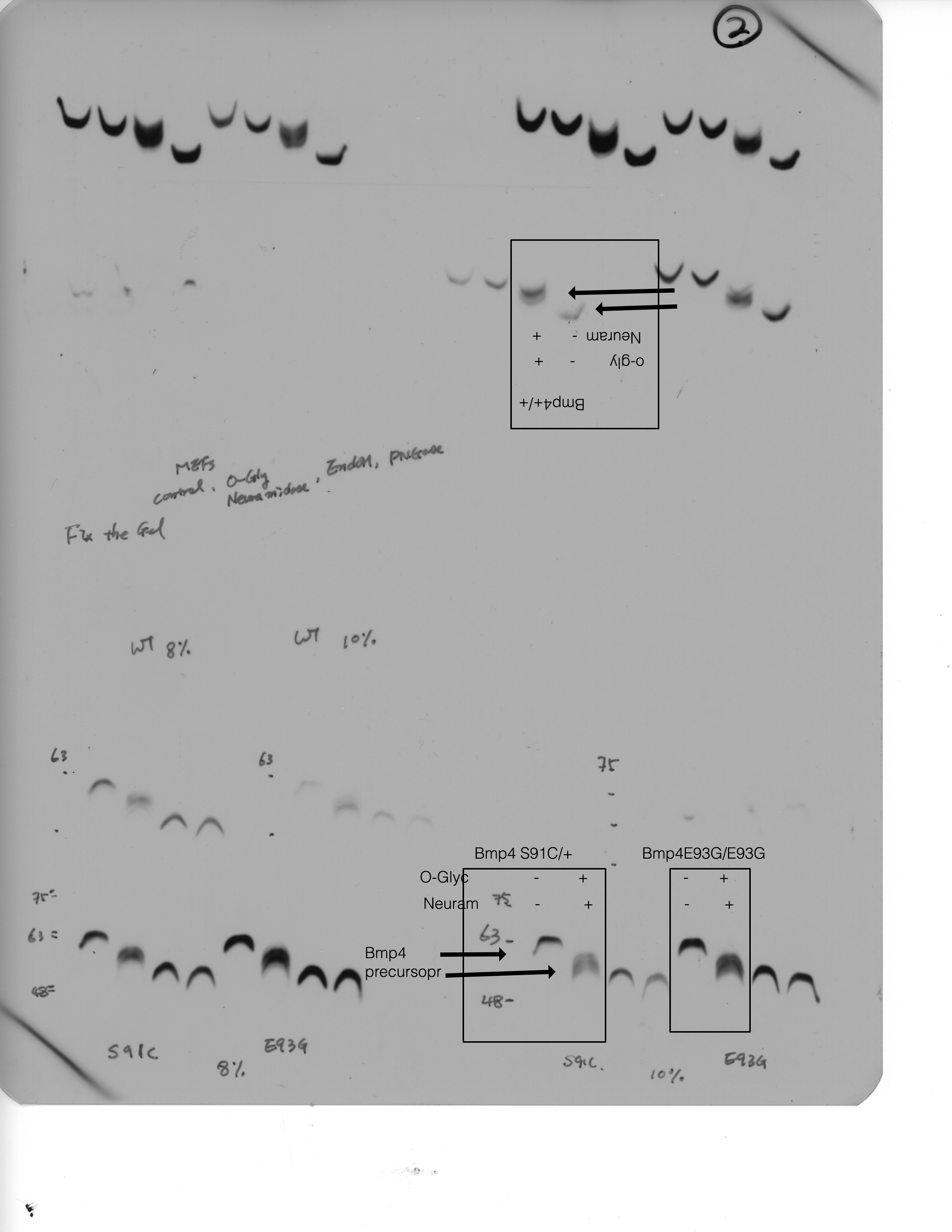

Supplement: Figure 7—source data 1. [file elife-105018-fig7-data1.zip › Figure 5-source data 1/Fig. 5A marked.tif]

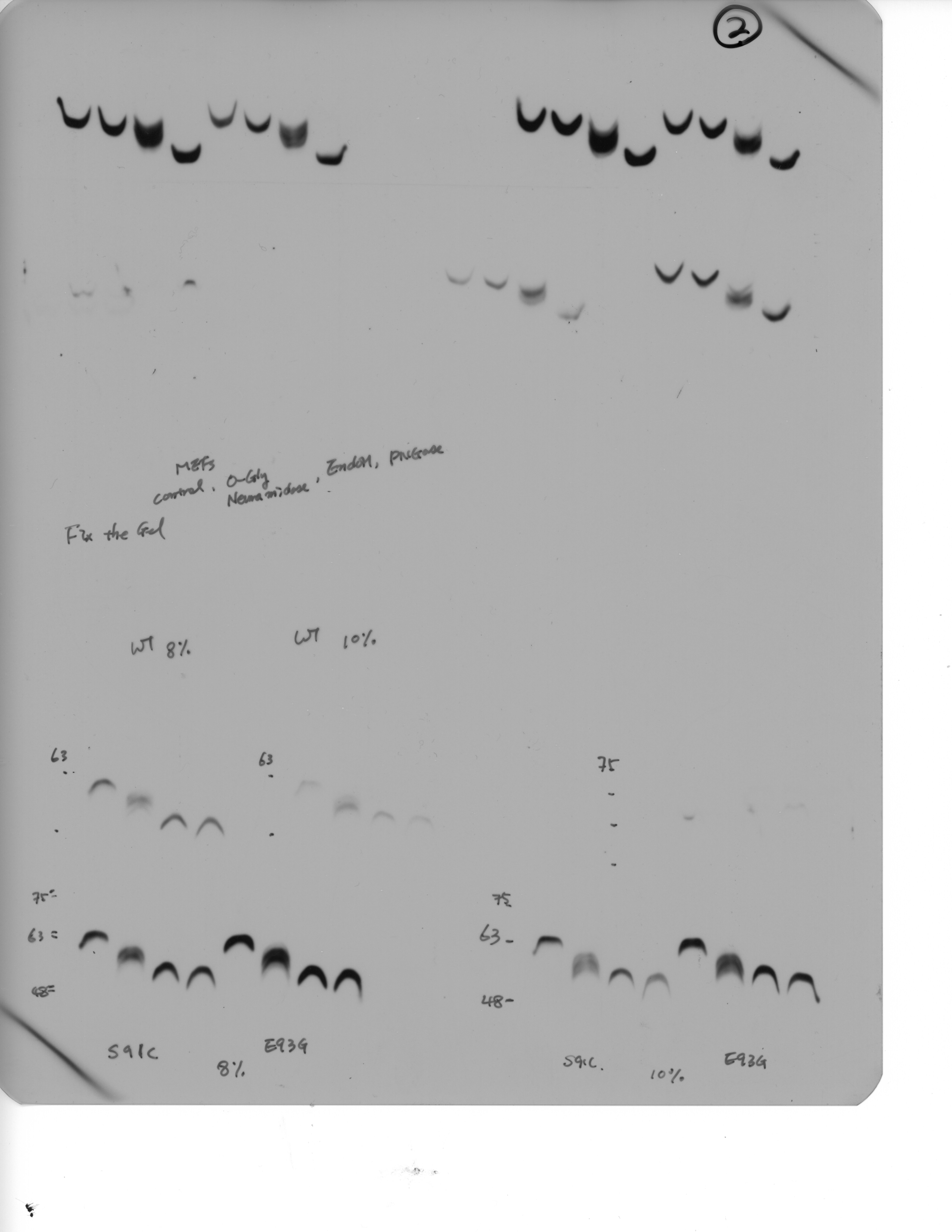

Supplement: Figure 7—source data 2. [file elife-105018-fig7-data2.zip › Figure 5-source data 2/J055_002 Fig. 5A unmarked.tif]

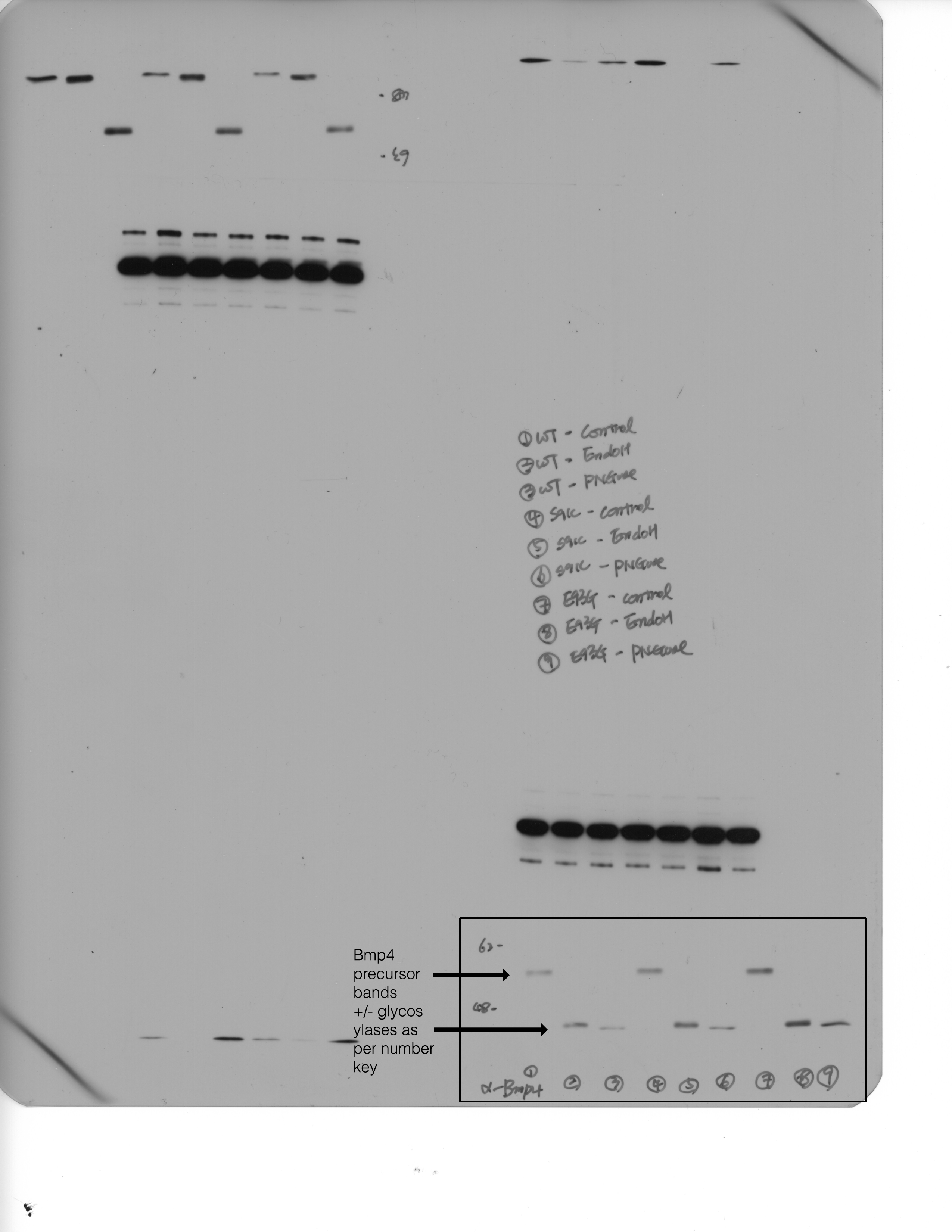

Supplement: Figure 7—source data 3. [file elife-105018-fig7-data3.zip › Figure 5-source data 3/J068_002 Fig. 5B source data marked.tif]

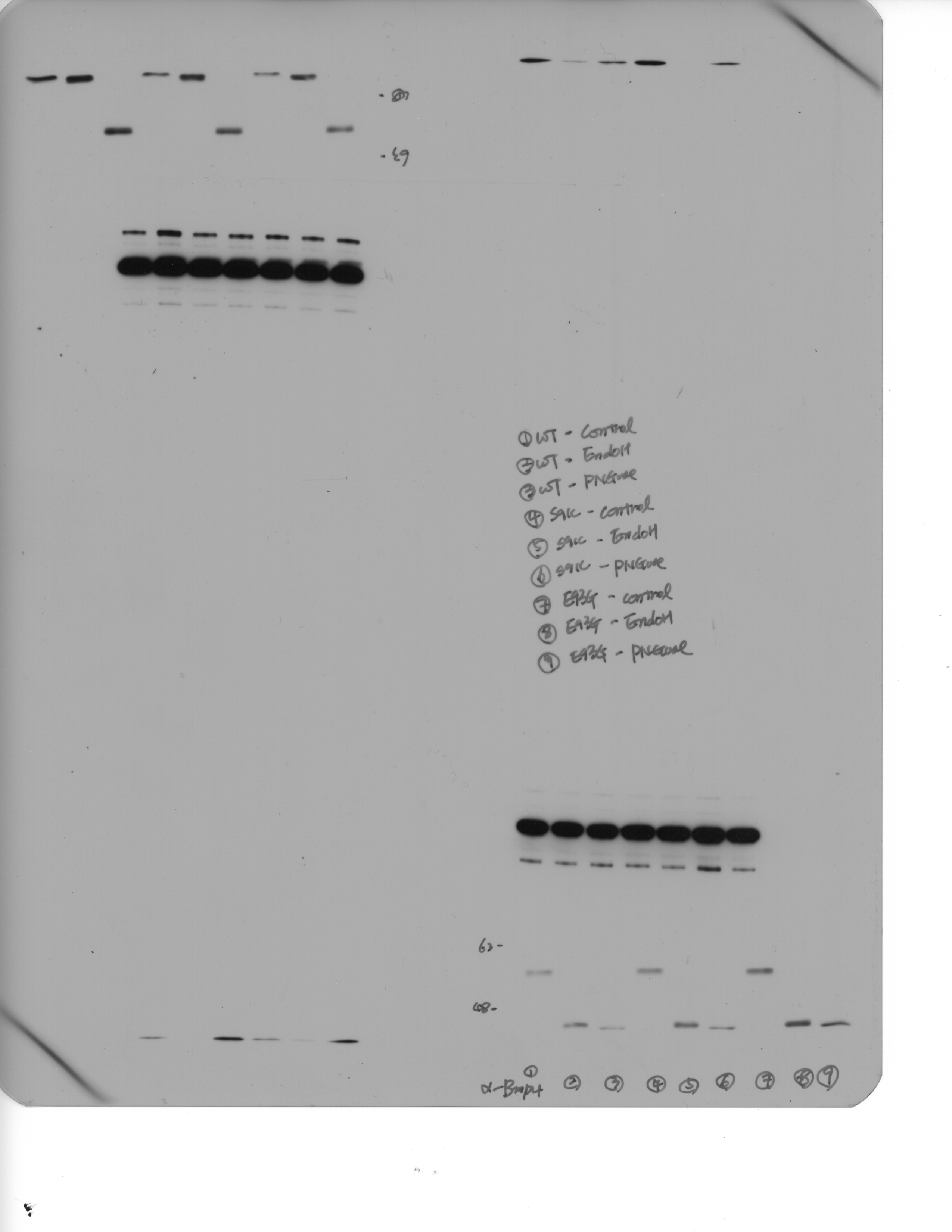

Supplement: Figure 7—source data 4. [file elife-105018-fig7-data4.zip › Figure 5-source data 4/J068_002 Fig. 5B source data unmarked.tif]
