# Supplementary material for: Mutations that prevent phosphorylation of the BMP4 prodomain impair proteolytic maturation of homodimers leading to lethality in mice
Source: eLife. 2025 May 29;14:RP105018. doi: 10.7554/eLife.105018 (PMC12122004; doi:10.7554/eLife.105018)
Supplement: Supplementary file 1. [file elife-105018-supp1.docx]

**Supplementary File 1; Supplementary Table 1. Progeny from *Bmp4^S91C/+^*  intercrosses at embryonic stages by sex.**

**A. Progeny from *Bmp4^S91C/+^* intercrosses at E13.5**

| **Sex** | ***Bmp4^+/+^*** | ***Bmp4^S91C/+^*** | ***Bmp4^S91C/S91C^*** | ***n*** | ***p*** |
| --- | --- | --- | --- | --- | --- |
| Both | 22 (19) | 55 (38) | 0 (19) | 77 | 0.00001 |
| Male | 12 (11) | 32 (22) | 0 (11) | 44 | 0.00015 |
| Female | 10 (8) | 23 (17) | 0 (8) | 33 | 0.00131 |

**B. Progeny from *Bmp4^S91C/+^* intercrosses at E11.5**

| **Sex** | ***Bmp4^+/+^*** | ***Bmp4^S91C/+^*** | ***Bmp4^S91C/S91C^*** | ***n*** | ***p*** |
| --- | --- | --- | --- | --- | --- |
| Both | 23 (16) | 39 (31) | 0 (16) | 62 | 0.00001 |
| Male | 15 (9) | 22 (19) | 0 (9) | 37 | 0.00118 |
| Female | 8 (6) | 17 (13) | 0 (6) | 25 | 0.01530 |

**C. Progeny from *Bmp4^S91C/+^* intercrosses at E10.5**

| **Sex** | ***Bmp4^+/+^*** | ***Bmp4^S91C/+^*** | ***Bmp4^S91C/S91C^*** | ***n*** | ***p*** |
| --- | --- | --- | --- | --- | --- |
| Both | 58 (60) | 135 (121) | 46 (60) | 239 | 0.072 |
| Male | 34 (33) | 69 (67) | 28 (33) | 131 | 0.630 |
| Female | 24 (27) | 66 (54) | 18 (27) | 108 | 0.065 |

**D. Progeny from *Bmp4^S91C/+^* intercrosses at E9.5**

| **Sex** | ***Bmp4^+/+^*** | ***Bmp4^S91C/+^*** | ***Bmp4^S91C/S91C^*** | ***n*** | ***p*** |
| --- | --- | --- | --- | --- | --- |
| Both | 7 (12) | 31 (24) | 9 (12) | 47 | 0.084 |
| Male | 4 (7) | 21 (15) | 4 (7) | 29 | 0.054 |
| Female | 3 (5) | 10 (9) | 5 (9) | 18 | 0.72 |

(A-D) Numbers of observed and expected (in parenthesis) embryos of each genotype listed in the top row are indicated. The p value is based on X2 test.
