# Supplementary material for: Mutations that prevent phosphorylation of the BMP4 prodomain impair proteolytic maturation of homodimers leading to lethality in mice
Source: eLife. 2025 May 29;14:RP105018. doi: 10.7554/eLife.105018 (PMC12122004; doi:10.7554/eLife.105018)
Supplement: Supplementary file 2. [file elife-105018-supp2.docx]

**Supplementary File 2, Supplementary Table 2. Progeny from *Bmp4^E93G/+^* x *Bmp4^-/+^*** **crosses at embryonic stages by sex.**

**A. Progeny from *Bmp4^E93G/+^* x *Bmp4^-/+^*** **crosses by sex at E13.5-14.5**

| **Sex** | ***Bmp4^+/+^*** | ***Bmp4^E93G/+^*** | ***Bmp4 ^-/+^*** | ***Bmp4^-/E93G^*** | ***n*** | ***p*** |
| --- | --- | --- | --- | --- | --- | --- |
| Both | 17 (17) | 13 (17) | 19 (17) | 17 (17) | 66 | 0.765 |
| Male | 10 (9) | 6 (9) | 9 (9) | 10 (9) | 35 | 0.746 |
| Female | 7 (8) | 7 (8) | 10 (8) | 7 (8) | 31 | 0.832 |

**B. Progeny from *Bmp4^E93G/+^* x *Bmp4^-/+^*** **crosses by sex at E11.5-12.5**

| **Sex** | ***Bmp4^+/+^*** | ***Bmp4^E93G/+^*** | ***Bmp4 ^-/+^*** | ***Bmp4^-/E93G^*** | ***n*** | ***p*** |
| --- | --- | --- | --- | --- | --- | --- |
| Both | 11 (10) | 4 (10) | 12 (10) | 14 (10) | 41 | 0.624 |
| Male | 6 (6) | 2 (6) | 8 (6) | 7 (6) | 23 | 0.307 |
| Female | 5 (5) | 2 (5) | 4 (5) | 7 (5) | 18 | 0.409 |

**C. Progeny from *Bmp4^E93G/+^* x *Bmp4^-/+^*** **crosses by sex at E10.5**

| **Sex** | ***Bmp4^+/+^*** | ***Bmp4^E93G/+^*** | ***Bmp4 ^-/+^*** | ***Bmp4^-/E93G^*** | ***n*** | ***p*** |
| --- | --- | --- | --- | --- | --- | --- |
| Both | 7 (7) | 10 (7) | 7 (7) | 5 (7) | 29 | 0.136 |
| Male | 3 (3) | 4 (3) | 4 (3) | 2 (3) | 13 | 0.838 |
| Female | 4 (4) | 6 (4) | 3 (4) | 3 (4) | 16 | 0.682 |

(A-C) Numbers of observed and expected (in parenthesis) embryos of each genotype listed in the top row are indicated. The p value is based on X2 test.
