# Supplementary material for: Mutations that prevent phosphorylation of the BMP4 prodomain impair proteolytic maturation of homodimers leading to lethality in mice
Source: eLife. 2025 May 29;14:RP105018. doi: 10.7554/eLife.105018 (PMC12122004; doi:10.7554/eLife.105018)
Supplement: Supplementary file 3. [file elife-105018-supp3.docx]

**Table S3. Primers used for PCR**

| **Primer Name** | **Sequence** |
| --- | --- |
| tbxt F | TTC TGA AGG TGA GCA TGT CG |
| tbxt R | GTT TGA CTT TGC TAA AAG AGA CAG G |
| odc F | TGC AGA GCC TGG GAG ATA CT |
| odc R | CAT TGG CAG CAT CTT CTT CA |
