# Supplementary material for: Mutations that prevent phosphorylation of the BMP4 prodomain impair proteolytic maturation of homodimers leading to lethality in mice
Source: eLife. 2025 May 29;14:RP105018. doi: 10.7554/eLife.105018 (PMC12122004; doi:10.7554/eLife.105018)
Supplement: MDAR checklist [file elife-105018-mdarchecklist1.docx]

**Materials:**

| Newly Created materials | Where Provided: |
| --- | --- |
| The manuscript includes a dedicated "materials availability statement" providing transparent disclosure about availability of newly created materials including details on how materials can be accessed and describing any restrictions on access. | Materials and Methods section |
| DNA and RNA sequences | **Where Provided:** |
| Primers used for RT-PCR | Table S3 |
| Primers used for Genotyping Mice | Materials and Methods |
| sgRNAs and single stranded donor DNA repair template oligonucleotides | Material and Methods |
| Cell Materials | **Where Provided:** |
| Cell Lines | N/A |
| Primary embryo fibroblast cultures from *Bmp4^HAMyc^*, *Bmp4^S91C^*, *Bmp4^E93G^*, and wild type littermate C57 Bl6 mice of both sexes | Materials and Methods |
| Experimental Animals | **Where Provided:** |
| Genetically modified and wild type C57 Bl6 *M. musculus* of both sexes | Materials and Methods, Key Resources Table |
| *Xenopus laevis* | Xenopus 1, Materials and Methods |
| Plants and microbes | **N/A** |
| Human Research Participants | **N/A** |

**Design:**

| Study Protocol | N/A |
| --- | --- |
| Laboratory Protocol | **Where Provided:** |
| Laboratory protocols | Materials and Methods |
| Experimental study design (statistics details) | |
| In vivo studies: | **Where Provided:** |
| Sample size determination | Yes-Figure legends; Fig. 2, 3, Fig. S3, S4, S5 and Methods section entitled “Statistics” |
| Randomization | N/A |
| Blinding | Yes-see Methods section entitled “In situ hybridization and ß-galactosidase staining “ |
| Inclusion/exclusion criteria | N/A |
| Sample definition and in-laboratory replication | **Where Provided:** |
| All experiments were conducted in a minimum of three biological replicates | Sample sizes are shown in Tables and Figure legends and represent biological replicates (individual samples or animals as indicated) |
| Ethics | **Where Provided:** |
| Studies involving human participants | N/A |
| Studies involving specimen and field samples | N/A |
| Dual use of Research Concern | **N/A** |

**Analysis:**

| Attrition | Where Provided: |
| --- | --- |
| Sample or data point exclusion | All data sets generated or analyzed are included in the figures. Litters of mouse embryos stained for BRE-LacZ activity in which either wild type or mutant littermates were found to be absent upon genotyping were excluded from reporting due to lack of relevant comparisons. |
| Statistics | **Where Provided:** |
| A student’s *t-*test was used to compare differences in gene expression or protein levels between two groups. | See Methods section entitled “Statistics” |
| Data Availability | **Where provided** |
| Data | All data associated with this study are present in the paper, Source data or the Supplementary Materials |
| Code availability | **N/A** |
| Adherence to community standards | **Where provided** |
| ARRIVE guidelines were used for reporting strain, sex, age, and genetic modifications of all mice; controls (littermates) and minimization of bias by blinding. | Materials and Methods |
